# Survival of Migrating Salmon Smolts in Large Rivers With and Without Dams 

**DOI:** 10.1371/journal.pbio.0060265

**Published:** 2008-10-28

**Authors:** David W Welch, Erin L Rechisky, Michael C Melnychuk, Aswea D Porter, Carl J Walters, Shaun Clements, Benjamin J Clemens, R. Scott McKinley, Carl Schreck

**Affiliations:** 1 Kintama Research, Nanaimo, British Columbia, Canada; 2 Fisheries Center, University of British Columbia, Vancouver, British Columbia, Canada; 3 United States Geological Survey, Oregon Cooperative Fish and Wildlife Research Unit, Department of Fisheries and Wildlife, Oregon State University, Corvallis, Oregon, United States of America; 4 Centre for Aquaculture and the Environment, University of British Columbia, West Vancouver, British Columbia, Canada; University of California Santa Barbara, United States of America

## Abstract

The mortality of salmon smolts during their migration out of freshwater and into the ocean has been difficult to measure. In the Columbia River, which has an extensive network of hydroelectric dams, the decline in abundance of adult salmon returning from the ocean since the late 1970s has been ascribed in large measure to the presence of the dams, although the completion of the hydropower system occurred at the same time as large-scale shifts in ocean climate, as measured by climate indices such as the Pacific Decadal Oscillation. We measured the survival of salmon smolts during their migration to sea using elements of the large-scale acoustic telemetry system, the Pacific Ocean Shelf Tracking (POST) array. Survival measurements using acoustic tags were comparable to those obtained independently using the Passive Integrated Transponder (PIT) tag system, which is operational at Columbia and Snake River dams. Because the technology underlying the POST array works in both freshwater and the ocean, it is therefore possible to extend the measurement of survival to large rivers lacking dams, such as the Fraser, and to also extend the measurement of survival to the lower Columbia River and estuary, where there are no dams. Of particular note, survival during the downstream migration of at least some endangered Columbia and Snake River Chinook and steelhead stocks appears to be as high or higher than that of the same species migrating out of the Fraser River in Canada, which lacks dams. Equally surprising, smolt survival during migration through the hydrosystem, when scaled by either the time or distance migrated, is higher than in the lower Columbia River and estuary where dams are absent. Our results raise important questions regarding the factors that are preventing the recovery of salmon stocks in the Columbia and the future health of stocks in the Fraser River.

## Author Summary

Miniature electronic technologies now allow researchers to track a salmon's migration from its birthplace in a river's headwaters in the Rocky Mountains to the North Pacific, opening a window on the mysteries of migration and survival. Surprisingly, outward migrating salmon (smolts) have similar survival during migration down dammed and undammed rivers, challenging widely held notions about factors affecting salmon abundance. Elements of the large-scale POST (Pacific Ocean Shelf Tracking) acoustic telemetry array revealed the migrations. Although salmon smolt survival to the Pacific Ocean was comparable in both the dammed Columbia and undammed Fraser rivers, it was higher in the Columbia once distance or travel time was taken into account—and higher within the hydropower system than below the dammed section. There is not yet enough evidence to determine whether (1) the Fraser has a problem that reduces salmon survival to that of a heavily dammed river or (2) factors other than dams play a larger, unsuspected role in salmon survival. Wherever future research leads on those questions, the new fish tagging technology has demonstrated itself as a useful tool for obtaining objective scientific data with important value in a number of public policy arenas.

## Abstract 3

Electronic tagging of two species of salmon smolts reveals similar survival rates during migration down the dammed Columbia and undammed Fraser Rivers, challenging popular notions about factors affecting salmon abundance.

## Introduction

Many Columbia River salmon stocks are listed as threatened or endangered [1,2], a result often attributed to the construction and operation of the Columbia River dams [3–5]. Here, we examine one phase of the lifecycle of Columbia River and Fraser River salmon stocks by comparing the freshwater survival of freely migrating salmon smolts down the extensively dammed Snake-Columbia River system with that of the same species migrating down the Thompson-Fraser River system, which lacks dams, using components of a large-scale acoustic telemetry system, the Pacific Ocean Shelf Tracking (POST) array.

The Fraser and Columbia are the two largest rivers on the west coast of North America and have, or formerly had, some of the world's major salmon runs [6]. Concurrent with the start of construction of the Federal Columbia River Power System in 1938, and especially following the completion of the last dam in the Snake River in 1975, major declines in abundance of adult salmon returning to the Columbia have occurred [2,7].

Much of the salmon decline from historic abundance occurred as a result of overfishing and habitat loss before 1938, when Bonneville, the first federal dam, became operational. However, continued sharp declines in abundance, particularly after 1977, when the last of the Snake River dams was completed, have focused much attention on the operation of the dams [4,8]*.* A total of 13 salmon stocks in the Columbia system are now listed as threatened or endangered, with Snake River spring/summer Chinook (Oncorhynchus tshawytscha) and steelhead (O. mykiss) formally listed as threatened in 1992 and 1997, respectively [1]. The poor adult return to the Snake River is variously ascribed to mortality on salmon smolts migrating to sea caused by the eight hydropower dams [1,2,9], habitat disruption [2,10], interactions with hatchery fish [11–13], and changes in ocean climate affecting salmon survival after the smolts leave the river [14–16].

The Fraser River lacks dams and lies directly north of the Columbia River; within these two watersheds, the Thompson and Snake rivers form major tributaries and are located in similar climatic zones. At the end of the last ice age, salmon colonized the upper Fraser watershed (including the Thompson River) from the upper Columbia River, thus providing a relatively recent genetic linkage [17]. There are thus broad similarities between the two river systems, making for an interesting comparison of salmon survival during the freshwater phase of the juvenile outmigration in rivers with and without dams.

Here, we compare the survival of two species of salmonid smolts in these rivers using acoustic and Passive Integrated Transponder (PIT) tags to measure survival from the upper reaches to the river mouth. Although identified as an important source of uncertainty [18,19], an objective measure of freshwater survival has only recently become available with the construction of PIT tag detectors at dams on the Columbia and the advent of miniature radio and acoustic transmitters that can be implanted into migrating smolts. Beginning in the 1990s, the survival of migrating smolts between dams in the Snake-Columbia River watershed was measured using PIT tag technology [20–22], a very short-range radio-frequency tag whose use is feasible because the dams channel smolts into close proximity (≤1 m) to the PIT tag detectors. Prior to the development of miniature acoustic and radio tags with much greater range, it was not possible to measure the survival of migrating smolts in large rivers lacking dams, such as the Fraser, because there was no way to channel tagged fish into close proximity to receivers to detect their arrival.

The POST array is a recently developed continental-scale acoustic tracking array that allows the movements and survival of individual fish to be measured directly [23–25]. Because it is based on an acoustic frequency that works in both seawater and freshwater, the technology allows tracking of fish as small as migrating salmon smolts (≥125 mm) out to sea. We measured the survival of freely migrating hatchery-reared spring Chinook and wild steelhead smolts migrating out of tributaries of the undammed Thompson-Fraser River system in spring 2004–2006 by surgically implanting them with individually identifiable acoustic tags [26] and detecting the subsequent arrival of each surviving animal at the Fraser River mouth and then in the ocean (Table 1; Video S1). In all years, animals selected for tagging showed evidence of undergoing smolting, a suite of physiological changes associated with migration to sea [27]: skin color was changing to silver, and the behavior of hatchery-reared smolts showed evidence of searching for an exit from the tanks, with individuals repeatedly probing the tank walls. Median migration times after release were rapid, and smolts arrived at the Fraser River mouth, some 340 km distant, within a period of 3–17 d. Estimated survival, using the Cormack-Jolly-Seber (CJS) mark-recapture framework, ranged from 4%–67% [28].

**Table 1:**
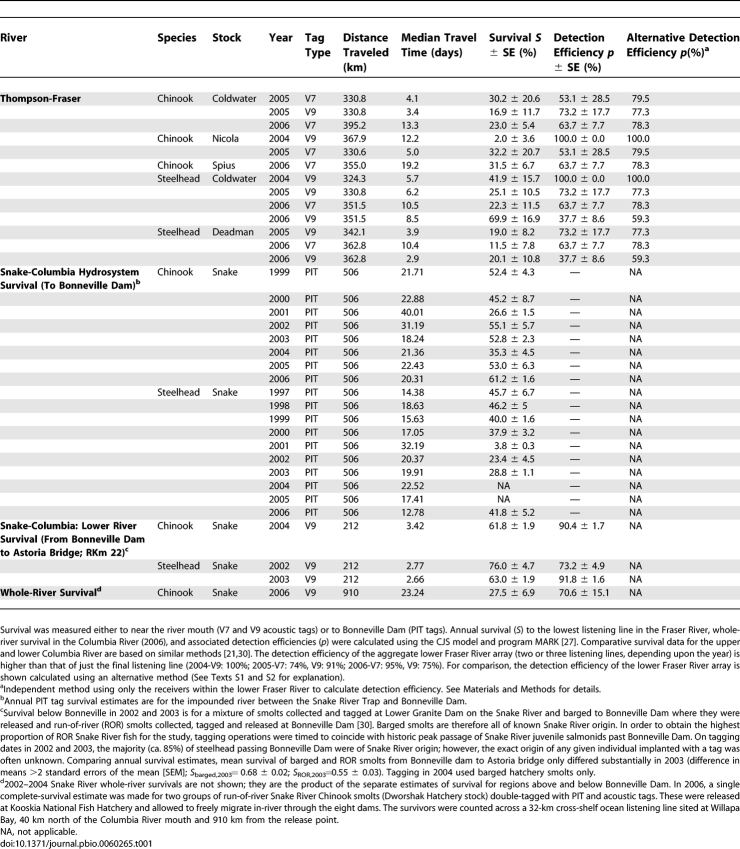
Estimated Survival of Snake and Thompson River Smolts

**Figure 1:**
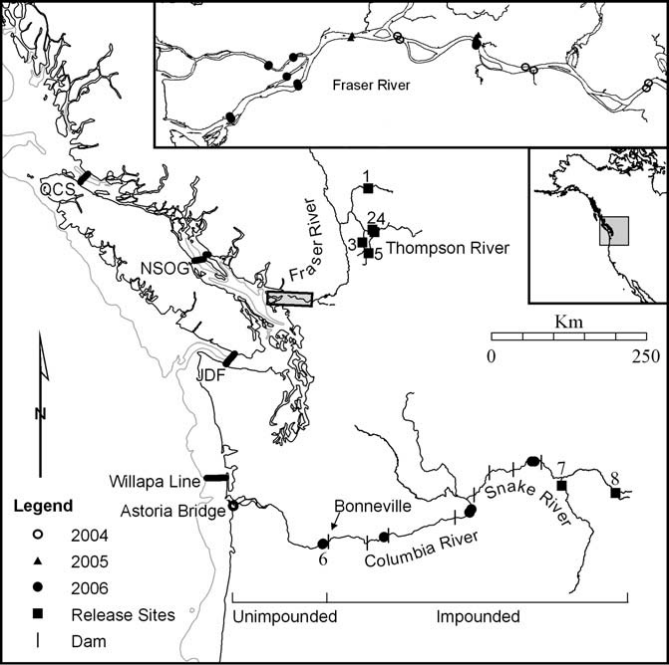
Geographic Location of Part of the POST Acoustic Array and Smolt Release Sites The edge of the continental shelf (200-m depth contour) is shown, as well as acoustic listening lines located in the two rivers and in the Strait of Juan de Fuca (JdF) and northern Strait of Georgia (NSOG). Position of the 2002–2004 lower Columbia River array at Astoria is described in [30]. Release sites, marked with a filled square are as follows: 1: Deadman River; 2: Nicola River; 3: Spius Creek; 4: Coldwater River; 5: Coldwater River; 6: Bonneville Dam; 7: Snake River trap; and 8: Kooskia National Fish Hatchery. Receiver locations marked with open circles indicate 2004; filled triangles 2005; and filled circles 2006.

The freshwater survival estimates for Thompson River smolts can be compared with two different measurements of survival of Snake River steelhead and Chinook smolts migrating down parts of the highly altered Snake-Columbia River system, which has eight major dams sited along the migration path (Figure 1). First, extensive measurements are available since 1997 of annual survival of PIT-tagged smolts migrating 516 km through the impounded section of the river from a release site in the Snake River at Lewiston, Idaho, through seven dams to the eighth and final dam at Bonneville on the Columbia River (river Km [RKm] 223) [21]. Second, survival in the unimpounded lower river and estuary from Bonneville Dam to Astoria Bridge (RKm 22) was measured in 2002–2004 using the same acoustic tag technology used in the Fraser River [29], providing an estimate of survival for the final, free-flowing section of the river, and which is consistent with radio tag estimates. Radio telemetry cannot be used to measure survival in the estuary, where saltwater is present. However, our survival estimates using radio telemetry for the region above the estuary, but below Bonneville dam, for the 3-y period 2002–2004 are similar to the survival estimates reported here that were obtained using acoustic telemetry [29,30].

Finally, a whole-river estimate of survival was derived by multiplying the PIT tag estimates of survival in the impounded upper river by the acoustic tag estimates of survival for the lower free-flowing river, providing a combined estimate of survival covering the entire river to Astoria, Oregon, for Snake River steelhead in 2002 and 2003 and for Snake River spring Chinook in 2004.

Because the importance of the results described below partly depends on the relative performance of the PIT and acoustic tag methodologies for measuring survival, we also used the same acoustic tag technology in 2006 to provide a single measurement of survival of hatchery-reared Snake River spring Chinook salmon migrating the entire 910-km length of the Snake-Columbia River system from release at the Kooskia National Fish Hatchery to a listening line situated in the ocean across the full width of the continental shelf at Willapa Bay, 40 km north of the Columbia River mouth [24]. The 2006 experiment thus provides a directly comparable whole-river survival estimate to those made in the free-flowing Thompson-Fraser system using identical acoustic tags and surgical protocols, as well as allowing a direct comparison of the survival of acoustic-tagged smolts with independent studies of the survival of PIT-tagged Snake River Chinook in the impounded section of the river that were made in the same year ([21]; see Video S1 for a comparison).

## Results

**Figure 2:**
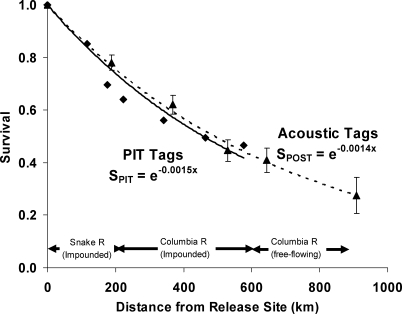
Comparison of 2006 Dworshak (Snake River) Spring Chinook Smolt Survival Estimates Using Acoustic Tags Measured with the POST Array with Published Data Using PIT tags Accoustic array data are shown as means ± 1 standard error (SE). The PIT tag data are from Tables 19 and 41 of [21]. The last two PIT tag data points are an aggregate of all Snake River spring Chinook tagged in the Snake River Basin. Regression estimates of the decline in survival with distance are statistically indistinguishable for Dworshak Hatchery smolts tagged with either PIT tags (diamonds) or acoustic tags (triangles) (*p* > 0.05), and are consistent with a single constant mortality rate above and below the hydropower system.

We first compared survival of PIT and acoustically tagged smolts in the impounded section of the Snake and Columbia rivers to assess survival of animals implanted with these different-sized tags in 2006 (Figure 2; [21]). Survival of acoustically tagged Snake River spring Chinook smolts from the Dworshak Hatchery stock (tagged and released at Kooskia Hatchery) was statistically indistinguishable from the estimated survival of PIT-tagged Dworshak Hatchery Chinook in 2006 (*p* > 0.05), demonstrating that the PIT and acoustic tag methodologies provide similar survival estimates for freely migrating smolts in the impounded section of the river. Of note, the decline in smolt survival with distance, evident for the POST data, suggests that a simple model of a constant freshwater mortality rate in the Columbia may be appropriate, irrespective of location in the river. However, we can not rule out the possibility that the decline in survival with distance may be punctuated, rather than smooth, at finer spatial scales not resolved by the current POST array.

**Figure 3:**
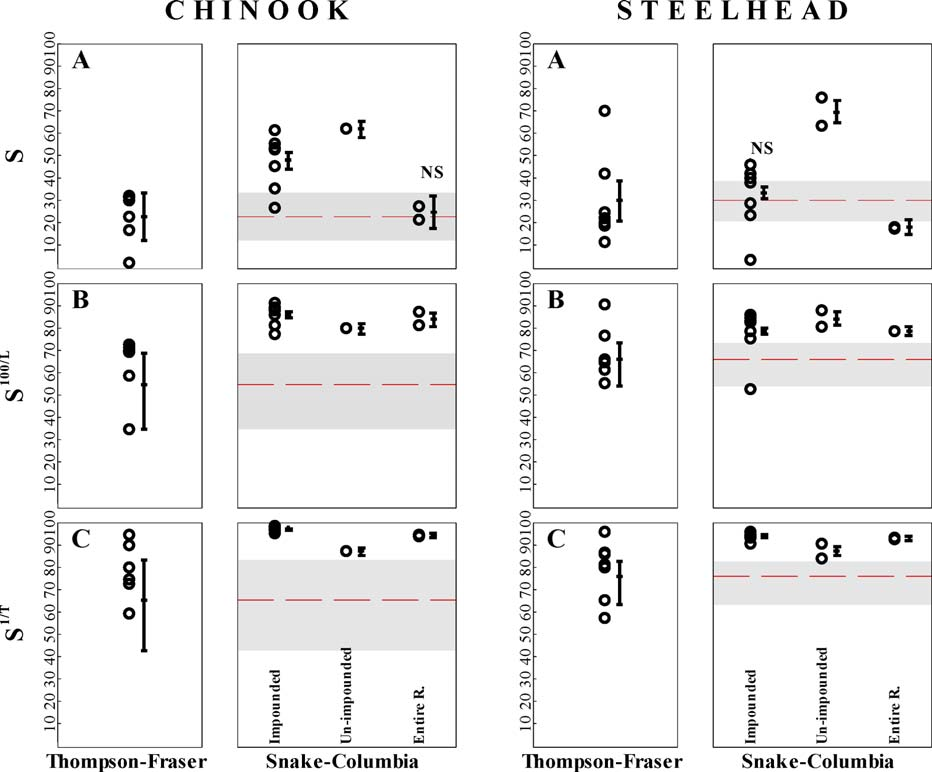
Annual Survival Estimates (%) for Thompson and Snake River Spring Chinook and Steelhead (A) Estimated survival. (B) Survival scaled per 100 kilometers traveled, *S* ^100/*L*^. (C) Survival scaled per migration day, *S* ^1/*T*^. For each species, the left panel shows the survival of different Thompson River stocks released to migrate down to the Fraser River mouth; the right panel shows the survival of Snake River stocks migrating down three sections of the Columbia River hydropower system: Impounded (upper river; eight dams), Unimpounded (lower river; undammed), and Entire river. The cross to the right of each group of individual survival estimates (open circles) shows the average survival and 95% confidence interval for the group, averaged across all available data (see Text S3 for details). For ease of comparison, the average Fraser River survival and 95% confidence limits are also drawn as a band across the Columbia River results. For Snake River stocks, impounded refers to survival measured using PIT tags from the Snake River trap to the last (Bonneville) dam (see Table 1). Unimpounded refers to survival measured from Bonneville Dam to near the river mouth (Astoria) using acoustic tags. The Chinook survival value for “Entire River” is based on the 2006 study using acoustically tagged Dworshak Hatchery smolts (tagged and released at Kooskia National Fish Hatchery) and whose survival was measured at an ocean listening line at Willapa Bay, 920 km distant. A single whole-river estimate is not available for steelhead, but a synthetic value can be obtained for 2002 and 2003 by multiplying up-river PIT tag survival by lower river acoustic tag survival, and scaling by total travel distance or time as appropriate. A similar combined estimate of survival for Chinook can be calculated from the 2004 data, and is also shown for comparison. In all comparisons of average river survival, Snake-Columbia River estimates were significantly different than Thompson-Fraser estimates at the 95% level unless indicated by “NS.”

Comparing survival between river systems, survival of smolts migrating the entire length of the river was either statistically indistinguishable (spring Chinook) between the undammed Thompson-Fraser River and the heavily impounded (eight dam) Snake-Columbia River system or slightly better in the Thompson-Fraser River (steelhead; Figure 3A). When considered separately by river section, survival of Snake River smolts through the eight dams comprising the impounded section of the river down to Bonneville Dam was higher (Chinook) or statistically indistinguishable (steelhead) from the survival for the entire Fraser River. For both species, survival in the free-flowing lower section of the Columbia River was higher than the entire-river estimate for the Fraser River.

These comparisons do not consider the distances and time that smolts must migrate to reach the location of the listening arrays in the two rivers; both values are substantially greater for Snake River smolts (Table 1). When scaled by either the migration distance or the median time for fish to reach the river mouth (Figure 3B and 3C), average survival rates of spring Chinook and steelhead are significantly higher for Snake River smolts than in the undammed Thompson-Fraser river system for all comparisons (*p* < 0.05). In fact, all annual survival estimates for Snake River spring Chinook in either the dammed or undammed sections of the river exceed the average survival of spring Chinook in the Thompson-Fraser system, and all but one annual survival estimate for Snake River steelhead exceeds the average survival for the Thompson-Fraser. It is also notable that within the Columbia, in most comparisons, survival scaled by distance or time was higher for the upstream impounded section of the river than in the lower, free-flowing, section of the river. It is striking that our main finding, that survival is not worse in the Columbia despite the presence of an extensive network of dams, remains the same no matter how the data are analyzed.

## Discussion

Comparable survival estimates to the river mouth of the Columbia (and higher survival rate when scaled by distance or time) appear at odds with the conventional view that the hydropower system is one of the major current limitations to salmon recovery and that a return to more normative, pre-dam conditions will aid in recovering salmon populations [31]. The conclusion that whole-river survival in both river systems is similar partly depends on the assumption that smolts migrate similarly after tagging, that detection at the mouth of the Fraser River was not seriously underestimated, and that the tagged animals are representative of the two whole populations in each river.

Several lines of evidence suggest that survival estimates in the Fraser-Thompson system are reasonable. First, migration to the river mouth of surviving smolts was rapid, with little evidence of delayed downstream movement, indicating a strongly directed migration. Second, systematic variation in the location and geometry of the lower Fraser River detection array over the 3-y period 2004–2006 always yielded detection efficiencies too high to alter the conclusion that survival rates scaled by either migration time or distance are higher on average for the Snake River populations. Because of the importance of this survival comparison to the public policy question concerning the impact of the dams on Columbia River salmon conservation, we made a particular effort in 2006 to build an extensive detection subarray consisting of six lines of paired acoustic receivers spaced a few kilometers apart within the lower Fraser River. This was done to ensure accurate detection estimates at the Fraser River mouth, as overestimation of the detection rate could potentially be incorrectly interpreted as poorer survival in the undammed river (fewer fish reaching the mouth of the river than was actually the case). This alternative approach to calculating detection efficiency at the Fraser River mouth (Table 1) also indicated a high detection efficiency, demonstrating that it is unlikely that survival in the Fraser was substantially underestimated.

There is a paucity of data concerning interannual variation in smolt survival in either system we studied. Although we can speculate that yearly variation in survival is probably quite substantial, we do not have additional data upon which to judge variation.

Third, in both rivers, the size of acoustic tags available limited our study to smolts from approximately the upper half of the size spectrum (V7 tags: smolts ≥125 mm; V9 tags: smolts ≥140-mm fork length). The size of the tagged smolts whose survival we compared was therefore roughly comparable between the two rivers, but the average size of the source populations from which our tagged animals were selected was smaller in the Thompson River. As larger salmon are generally found to have better survival [32,33], it seems likely that our current inability to tag the entire size range of migrating animals should be more likely to bias estimated survival upwards to a greater degree for the Thompson than the Snake River populations. Countering this empirical observation of higher survival in larger animals (which is based on a much wider range of sizes than is representative of just-migrating salmon smolts), we note that our measured survival in 2006 using acoustic tags for Snake River smolts was almost identical to that obtained in the same year using PIT tags, which cover the entire size range of migrating smolts (Figure 2). Finally, a detailed examination of how Snake River survival varied in 2006 for successive 5-mm size groupings of acoustically tagged smolts [24] found no consistent relationship between smolt size and survival. Our tentative conclusion, therefore, is that survival rates of similar-sized animals are lower in the Thompson-Fraser system and that any dependence of survival on size would likely strengthen this conclusion when extended to the entire population. However, as technology develops further and reduces the size of acoustic tags, it would clearly be desirable to tag the entire range of smolt sizes and directly test this question.

Evidence for stable rates of survival prior to smolt migration both before and after hydropower system completion [34] and the relatively high survival rates reported in this paper for migration down through the hydropower system and to the ocean (20%–30%) sharply contrast with the very poor survival until the adults return from the ocean (as little as 0.5% in some years [1,2,35]). The available data, therefore, indicate that although 25%–60% of smolts survive through the entire hydropower system, few return from the ocean. Thus, much of the mortality lies beyond the hydropower system, consistent with recent evidence that extensive effort put into freshwater habitat restoration may be insufficient by itself to conserve salmon populations [36]*.*

Dam operation clearly had large impacts on the mortality of migrating salmon smolts in the 1960s and 1970s [3,37], and changes to the hydropower system since then have improved survival substantially [22,38]. Our results suggest that survival through the hydropower system has now increased to levels similar to those experienced in both the undammed lower Columbia River and in the Fraser River, an important finding that was not technically possible before the development of the POST array. It remains unclear whether the similar rates of survival we measured result from past efforts to improve hydropower operations and reduce predators in the Columbia [39,40] or from unidentified problems in the Fraser River. Thompson River spring Chinook abundance has been stable or increasing since 1980, whereas Thompson River steelhead are classified as of “Extreme Conservation Concern” [41]. However, as with many other steelhead populations located in southern British Columbia, the available evidence suggests the conservation status of Thompson River steelhead is primarily caused by poor marine survival after passage out of the river [42–44]. Poor smolt-to-adult survival is also observed for both species of Snake River smolts after migration out of the hydropower system; therefore, a common effect of ocean conditions on survival of salmon in both river systems seems likely.

Modifications to dam design and operation have increased Columbia River smolt survival in the past 20 y [5,39,45,46]. Our initial results from the use of large-scale acoustic arrays over 5 y together with PIT tag data suggest that the overall migratory survival of salmon smolts in the Columbia and Fraser systems is now similar. This result is surprising, given that dams are often implicated as major barriers to recovery in the Columbia. However, our data do not address whether the possible delayed effects of hydropower system passage subsequently affects mortality after the fish leave the river for the ocean [9,48], currently a contentious issue, nor is it clear whether survival in the Fraser River has changed during the last 100 y, as prior baseline measurements of survival are absent. There are several opposing inferences that can be made from our findings regarding the role of dams in preventing the recovery of salmon. We suggest that conservation efforts in the Columbia may be better directed towards understanding the effects of hydropower system passage on ocean survival, in addition to the extraction of small gains in survival at the dams.

## Materials and Methods

Detailed surgical protocols are described elsewhere [49]. Briefly, individually identifiable Vemco (http://www.vemco.com/products/transmitters/index_coded.php) V9-6L acoustic tags (9-mm diameter, 20-mm long) were surgically implanted into the abdominal cavities of smolts ≥140 mm in both the Thompson and Snake river stocks. Vemco V7-2L acoustic tags (7 mm in diameter, 22-mm long) were surgically implanted into some groups of Thompson River smolts in the 125–140-mm size range; these groups are identified in Table 1. Surgical procedures were annually reviewed and approved by institutional animal care committees.

Elements of the POST acoustic array were used to measure the survival of the acoustically tagged smolts. POST is a large-scale passive acoustic telemetry system that sits on the sea floor and in sections of the Columbia and Fraser rivers (http://www.postcoml.org). POST was designed [23] to provide a precise spatial geometry for a multitude of individually low-cost acoustic receivers that records the time of detection of individual acoustic tags; the programming of the acoustic tags was chosen to complement this geometry and to provide both high tag detection efficiencies and very long life for the tags. The full spatial scale of the array currently extends 2,500 km from Oregon to Alaska, and is described elsewhere [24]; data on the position of the entire POST array, including the Fraser River subarray positions, are reported in the POST database (http://www.postcoml.org/page.php?section=database), as are the detection histories of all Thompson River tagged smolts and the Snake River smolts tagged in 2006. An animation of the movements of some of the tagged smolts on which this paper is based is shown in Video S1. Summary data on the surgical procedures and receiver arrays used for Columbia River smolts in years prior to 2006 are similar and are described in [30].

Detection efficiencies at each line of acoustic receivers within the Fraser River were estimated for each type of tag and year, as V7 tags have a lower acoustic power output than V9 tags (136 versus 149 dB re: 1 μPa at 1 m) and the geometry of the Fraser River mouth array varied between years. Aggregate detection efficiency of the ocean listening lines (all years combined) was estimated as 89.5% for V9 tags and 71.4% for V7 tags, thus providing a good estimate of total tagged smolts migrating out of the Fraser River. All Columbia River tagging used V9-6L tags; data and protocols for years prior to 2006 are described in [30]; in 2006, the array was extended upstream as far as the Snake River and out into the ocean (these data are available from the POST database).

Dworshak Hatchery spring Chinook, a Snake River stock, were transferred to Kooskia National Fish Hatchery in the spring of 2006 and held until surgical implantation with acoustic tags and subsequent release at Kooskia. Snake River smolts were double-tagged with a PIT tag in 2006 to ensure that they were not inadvertently collected at the dams for transportation in barges and were thus forced to migrate the full length of the river. We compare their measured survival using the acoustic array with the survival of the Dworshak stock of Snake River spring Chinook smolts independently measured using the PIT tag system in the same year [21].

Survival estimates in the Columbia River measured using PIT and POST acoustic tags were regressed against distance from release site, *L*, after log-transformation using a fixed intercept, *S*(*L*) = exp(−*zL*), yielding an estimate of the survival rate per river kilometer. PIT tag estimates of survival were measured at the dams; acoustic tag survival estimates were derived from the four in-river detection subarrays extending from the Snake River to just below Bonneville Dam plus the ocean listening line at Willapa Bay (see Figure 1). Regression coefficients of the survival rate of Dworshak Hatchery smolts were statistically indistinguishable between PIT and acoustically tagged smolts (*p* > 0.05).

More extensive descriptions of the statistical measurement of survival using the acoustic array and the performance of the array is available in Text S1–S3. The MatLAB code used to generate the Monte Carlo statistical comparisons, and the frequency histograms of the generated data are reported in Text S4.

## Supporting Information

Text S1 Methods for Calculating Detection Efficiency in the Lower Fraser River, 2006 (108 KB PDF)

Text S2 Alternative Detection Efficiency Calculation Using Individual Listening Lines within the Fraser River (91 KB PDF)

Text S3 Tests of Statistical Significance for Figure 3
(101 KB PDF)

Text S4 MatLAB Scripts, Data, and Frequency Histograms Showing Results from the Monte Carlo Analyses (351 KB PDF)

Video S1 Movement of Tagged Snake River Spring Chinook (2006) and Thompson River Spring Chinook and Steelhead (2005–2006) over the POST Array The array is shown in magenta. In some cases, movements have been specified as straight lines because of uncertainty as to the exact path chosen. This animation has been created using the XVid open source codec; the codec can be uploaded from http://www.xvidmovies.com/codec/ if the animation is not visible.
(1.98 MB AVI)

## Abbreviations

PIT: Passive Integrated Transponder
POST: Pacific Ocean Shelf Tracking
RKm: river Km
