# Correction: Survival of Migrating Salmon Smolts in Large Rivers With and Without Dams

**DOI:** 10.1371/journal.pbio.0060314

**Published:** 2008-12-23

**Authors:** David W Welch, Erin L Rechisky, Michael C Melnychuk, Aswea D Porter, Carl J Walters, Shaun Clements, Benjamin J Clemens, R. Scott McKinley, Carl Schreck

Correction for:

Welch DW, Rechisky EL, Melnychuk MC, Porter AD, Walters CJ, et al. (2008) Survival of migrating salmon smolts in large rivers with and without dams. PLoS Biol 6(10): e265 doi:10.1371/journal.pbio.0060265

In the legend below Table 1, the reference given at the end of the second sentence is incorrect. The sentence should read:

“Annual survival (S) to the lowest listening line in the Fraser River, whole-river survival in the Columbia River (2006), and associated detection efficiencies (p) were calculated using the CJS model and program MARK [28]”

