# Supplementary material for: Survival of Migrating Salmon Smolts in Large Rivers With and Without Dams
Source: PLoS Biol. 2008 Oct 28;6(10):e265. doi: 10.1371/journal.pbio.0060265 (PMC2573937; doi:10.1371/journal.pbio.0060265)
Supplement: Text S1 — (108 KB PDF) [file pbio.0060265.sd001.pdf]

Survival of Migrating Salmon Smolts in Large Rivers With and Without Dams

PLoS Biology (2008) 6(10):e265. doi:10.1371/journal.pbio.0060265

S.1 Methods for calculating detection efficiency in the lower Fraser River 2006

We used the Cormack-Jolly-Seber (CJS) recaptures-only model in Program MARK to simultaneously estimate survival and detection probabilities. First, we estimated a variance inflation factor ($\hat{c}$) to compensate for extra-binomial variation in estimated probabilities [i] using MARK's goodness-of-fit bootstrapping routine. We used the general CJS model, $\phi_{(time*group)}p_{(time*group)}$, where the "time" factor represents re-capture locations at receiver lines and the group factor represents possible combinations of species, stock, and tag type in a given year. Values of $\hat{c}$ were similar in the Fraser (2004: 3.12; 2005: 1.70; 2006: 1.56) and Columbia (2006: 1.87) rivers.

We modeled detection histories of individual fish at sequential receiver (i.e., "re-capture") lines within rivers. To reduce the number of estimated parameters, all detections on ocean lines of receivers were lumped together as a single digit representing the final capture history. In the Columbia River, we used a fully time-varying model in survival and detection probabilities for the single population in 2006 (all fish had V9 tags). In the Fraser River, with multiple populations released each year, we used a fully time and group varying model in survival and a time-varying model in detection efficiencies with common tag types aggregated together: $\phi_{(time*group)}p_{(time+tag\ type)}$. This model for p is additive, such that in logit space the difference between detection efficiencies of the two tag types remains constant across all receiver lines. A fully

interacting model allowing for separate differences in detection efficiency between tag types at receiver lines, $p_{(time*tag\ type)}$, yielded similar estimates of detection and thus survival probabilities.

We calculated the product of segment-specific survival probabilities within rivers to generate a full-river estimate of survival from release until the last in-river receiver line. We used the Delta method to calculate the variance of this product.

---

[i] K. P. Burnham, D. R. Anderson, G. C. White, C. Brownie, K. H. Pollock. *Design and analysis methods for fish survival experiments based on release-recapture*. (American Fisheries Society Monograph 5, Bethesda, MD, 1987).
