# Supplementary material for: Survival of Migrating Salmon Smolts in Large Rivers With and Without Dams
Source: PLoS Biol. 2008 Oct 28;6(10):e265. doi: 10.1371/journal.pbio.0060265 (PMC2573937; doi:10.1371/journal.pbio.0060265)
Supplement: Text S2 — (91 KB PDF) [file pbio.0060265.sd002.pdf]

Survival of Migrating Salmon Smolts in Large Rivers With and Without Dams

PLoS Biology (2008) 6(10):e265. doi:10.1371/journal.pbio.0060265

S.2 Alternate Detection Efficiency Calculation using Individual Listening Lines within the River

In the lower Fraser River in 2006 we built an extensive sub-array of multiple closely spaced listening lines (12 receivers; 6 listening lines) as a check on the detection efficiency estimates that were calculated using the ocean listening lines. We first estimated the detection efficiency of each individual river line by tag type, species, and stock. For the Derby Reach line (sited well-upstream), detection efficiencies were estimated as the percentage of fish detected on all downstream lines that were also detected on the Derby Reach line. For the South and North Arm upstream lines, detection efficiencies were estimated as the percentage of fish detected on their respective downstream lines that were also detected on the upstream lines. For the downstream lines, this calculation was reversed and detection efficiencies were estimated as the percentage of fish detected on the respective upstream lines that were also detected on the downstream lines. This reverse calculation may result in a slight underestimate of detection efficiency because some of the fish migrating downstream may have died between the upstream and downstream lines. However, the lines are spaced 4.3 & 9.6 km apart, which is small relative to the 320 km migration path.

The detection efficiency for the lower Fraser River as a whole was calculated as the probability of a fish not being detected on any of the listening lines along its migration route within the lower river. All tagged Fraser fish first passed by the Derby Reach

listening line in 2006, but they could then exit to the ocean via either the South or the North Arms of the Fraser. We accounted for these two exit routes by first calculating the detection efficiency of each Arm as the probability of being missed by both the upstream and downstream listening lines of each arm (all lines were composed of a pair of receivers sited on opposing sides of the river; the detections from these pairs of receivers were treated as one detection line), and then averaging these "arm-specific" probabilities by weighting by the number of fish observed exiting via that route. We estimated the total number of fish exiting via each arm by dividing the number of fish recorded by the detection efficiency for that arm. We then multiplied the probability of a fish failing to be detected on the Derby Reach line by the weighted probability of being missed on either the South or North Arm lines to estimate the detection efficiency of the lower Fraser River as a whole. A similar procedure was used in 2004 and 2005, but in this case the array was formed of 3 pairs of receivers on the Fraser main stem. Additional receivers were used in 2006 to ensure that a very high detection efficiency was obtained in 2006, as the key question is whether a substantial number of tagged smolts could survive to leave the Fraser R undetected, and thus unduly compromise the conclusion that survival is similar to that measured in the Columbia hydropower system. These alternate detection efficiencies were then compared with the conventional CJS estimates, as a check on the conventional estimation procedure.
