# Supplementary material for: Survival of Migrating Salmon Smolts in Large Rivers With and Without Dams
Source: PLoS Biol. 2008 Oct 28;6(10):e265. doi: 10.1371/journal.pbio.0060265 (PMC2573937; doi:10.1371/journal.pbio.0060265)
Supplement: Text S3 — (101 KB PDF) [file pbio.0060265.sd003.pdf]

Survival of Migrating Salmon Smolts in Large Rivers With and Without Dams

PLoS Biology (2008) 6(10):e265. doi:10.1371/journal.pbio.0060265

S.3 Tests of Statistical Significance for Figure 3.

To calculate the average survival and 95% confidence interval for Fraser R Chinook and steelhead shown in Fig. 3 we used the following Monte Carlo procedure: For the j=1,…,J annual survival estimates available for each species (for example, $J_{Chinook}$=6; $J_{Steelhead}$=7 in the Thompson), we calculated the average of J survival estimates by drawing from component normalized binomial distributions $S_j \sim \frac{1}{N_j^*} B(\overline{S}_j, N_j^*)$ that describe the expected probability density function of each survival observation. This was repeated 10,000 times to provide a set of results, each describing the outcome from calculating the average across J observations, with the individual survival observations weighted by their relative uncertainty.

Here $S_j \sim \frac{1}{N_j^*} B(\overline{S}_j, N_j^*)$ is the normalized binomial distribution for the jth observation of survival with observed survival proportion $\overline{S}_j$ and sample size N_j^* (the latter parameter determines the fatness of the tails). The set of M=10,000 survival estimates was used to calculate the average and the interval defining the upper and lower 2.5 percentiles of the distribution. Here $\overline{S}_j$ is the observed survival for the jth group calculated from the CJS model, but we replace the number of fish released, N_j, by N_j^*, where N_j^* is that value of N that will generate the variance on S estimated by the CJS model. (We use the fact that the standard error on a proportion S_j defined by a binomial distribution can be written as

SE(S_j)=$\sqrt{\frac{S_j(1-S_j)}{N}}$; knowing S_j and its standard error, we can solve for the value of N_j^* that yields this standard error using the binomial distribution, which is $N_j^*=\frac{S_j(1-S_j)}{SE(S_j)^2}$. This is a large sample Gaussian approximation to the binomial appropriate when SN≥5, which is true for all of the annual estimates). This approach was taken because the variance of S when the detection efficiency of the array is unknown is greater than if the detection efficiency is known. In general, this is a conservative test for our purposes because $N^*<N$, and results in sampling from a fatter-tailed distribution. A similar calculation was done to calculate the mean & 95% confidence interval on the average survival estimates reported for the three sections of the Columbia River reported in Fig. 3.

To compare whether survival estimates for Thompson-Fraser fish are different from survival estimates for smolts in the Columbia R, we used a similar procedure, based on calculating the statistical distribution of the difference between pairs of survival estimates for the two rivers. For each of the three Columbia R groups (up-river (impounded), lower-river, or entire river), we calculated a separate set of 10,000 survival estimates formed by averaging over the set of j=1,…, J observations, $S_j \sim \frac{1}{N_j^*}B(\bar{S}_j, N_j^*)$, available, and where the N_j^* values are those needed to yield the reported standard errors on the survival estimates. A separate set of 10,000 survival estimates were drawn using the Fraser R parameters, and the pair-wise set of differences was calculated. If there is no difference in survival between the two rivers, then the difference between these pair-wise

values should be zero on average. We accepted the hypothesis of no difference in survival if the 95% confidence interval on these pair-wise differences included zero. We ran all experiments multiple times to ensure that the conclusions were stable across repeated runs.
