# Supplementary material for: Survival of Migrating Salmon Smolts in Large Rivers With and Without Dams
Source: PLoS Biol. 2008 Oct 28;6(10):e265. doi: 10.1371/journal.pbio.0060265 (PMC2573937; doi:10.1371/journal.pbio.0060265)
Supplement: Text S4 — (351 KB PDF) [file pbio.0060265.sd004.pdf]

Survival of Migrating Salmon Smolts in Large Rivers With and Without Dams
PLoS Biology (2008) 6(10):e265. doi:10.1371/journal.pbio.0060265

S.4 MatLAB Scripts, Data, & Frequency Histograms Showing Results from the Monte Carlo Analyses

Chinook Results

- SNAKE RIVER CHINOOK IMPOUNDED
- #1SNAKE RIVER CHINOOK IMPOUNDED comparison with FRASER RIVER CHINOOK
- SNAKE RIVER CHINOOK UNIMPOUNDED
- #2SNAKE RIVER CHINOOK UNIMPOUNDED comparison with FRASER RIVER CHINOOK
- COLUMBIA-SNAKE RIVER-ENTIRE CHINOOK 2006
- COLUMBIA-SNAKE RIVER-ENTIRE CHINOOK 2004
- COLUMBIA-SNAKE RIVER-ENTIRE CHINOOK (2006 and 2004 combined)
- #3COLUMBIA-SNAKE RIVER-ENTIRE CHINOOK comparison with FRASER RIVER CHINOOK
- FRASER RIVER CHINOOK scaled by distance (per 100 km)
- SNAKE RIVER CHINOOK IMPOUNDED scaled by distance (per 100 km)
- #4SNAKE RIVER CHINOOK IMPOUNDED comparison with FRASER CHINOOK scaled by distance
- SNAKE RIVER CHINOOK UNIMPOUNDED scaled by distance (per 100 km)
- #5SNAKE RIVER CHINOOK UNIMPOUNDED comparison with FRASER CHINOOK scaled by distance
- COLUMBIA-SNAKE ENTIRE CHINOOK scaled by distance (per 100 km) 2006
- COLUMBIA-SNAKE ENTIRE CHINOOK scaled by distance (per 100 km) 2004
- COLUMBIA-SNAKE ENTIRE CHINOOK scaled by distance (per 100 km) (2006 and 2004)
- #6COLUMBIA-SNAKE ENTIRE CHINOOK comparison with FRASER CHINOOK scaled by distance (per 100 km)
- FRASER RIVER CHINOOK scaled by time (per day)
- SNAKE RIVER CHINOOK IMPOUNDED scaled by time (per day)
- #7SNAKE RIVER CHINOOK IMPOUNDED comparison with FRASER CHINOOK scaled by time (per day)
- SNAKE RIVER CHINOOK UNIMPOUNDED scaled by time (per day)
- #8SNAKE RIVER CHINOOK UNIMPOUNDED comparison with FRASER CHINOOK scaled by time (per day)
- COLUMBIA-SNAKE ENTIRE CHINOOK scaled by time (per day) 2006
- COLUMBIA-SNAKE ENTIRE CHINOOK scaled by time (per day) 2004
- COLUMBIA-SNAKE ENTIRE CHINOOK scaled by time (per day) (2006 and 2004 combined)
- #9COLUMBIA-SNAKE ENTIRE CHINOOK comparison with FRASER CHINOOK scaled by time (per day)

Steelhead Results

- FRASER RIVER STEELHEAD
- SNAKE RIVER STEELHEAD IMPOUNDED
- #1SNAKE RIVER STEELHEAD IMPOUNDED comparison with FRASER RIVER STEELHEAD
- SNAKE RIVER STEELHEAD UNIMPOUNDED
- #2SNAKE RIVER STEELHEAD UNIMPOUNDED comparison with FRASER RIVER STEELHEAD
- COLUMBIA-SNAKE RIVER ENTIRE STEELHEAD 2002
- COLUMBIA-SNAKE RIVER-ENTIRE STEELHEAD 2003
- #3COLUMBIA-SNAKE RIVER ENTIRE STEELHEAD comparison with FRASER RIVER STEELHEAD
- FRASER RIVER STEELHEAD scaled by distance (per 100 km)
- SNAKE RIVER STEELHEAD IMPOUNDED scaled by distance (per 100 km)
- #4SNAKE RIVER STEELHEAD IMPOUNDED comparison with FRASER STEELHEAD scaled by distance (per 100 km)
- SNAKE RIVER STEELHEAD UNIMPOUNDED scaled by distance (per 100 km)
- #5SNAKE RIVER STEELHEAD UNIMPOUNDED comparison with FRASER STEELHEAD scaled by distance (per 100 km)
- COLUMBIA-SNAKE ENTIRE STEELHEAD scaled by distance (per 100 km) 2002
- COLUMBIA-SNAKE ENTIRE STEELHEAD scaled by distance (per 100 km) 2003
- #6COLUMBIA-SNAKE ENTIRE STEELHEAD comparison with FRASER STEELHEAD scaled by distance (per 100 km)
- FRASER RIVER STEELHEAD scaled by time (per day)
- SNAKE RIVER STEELHEAD IMPOUNDED scaled by time (per day)
- #7SNAKE RIVER STEELHEAD IMPOUNDED comparison with FRASER STEELHEAD scaled by time (per day)
- SNAKE RIVER STEELHEAD UNIMPOUNDED scaled by time (per day)
- #8SNAKE RIVER STEELHEAD UNIMPOUNDED comparison with FRASER STEELHEAD scaled by time (per day)
- COLUMBIA-SNAKE ENTIRE STEELHEAD scaled by time (per day) 2002
- COLUMBIA-SNAKE ENTIRE STEELHEAD scaled by time (per day) 2003
- #9COLUMBIA-SNAKE ENTIRE STEELHEAD comparison with FRASER STEELHEAD scaled by time (per day)

Chinook Results

```
%This script will test the hypothesis that the estimated survivals for
%Fraser River and Columbia-Snake River are equal
%We will use the randraw.m script ('EFFICIENT RANDOM VARIATES
GENERATOR'
%from a binomial distribution)
%For the Fraser River Chinook - there are 6 survival estimates
available
%For the Columbia River Chinook - there are 8 PIT tag survival
estimates
%available for the Upper Columbia River, and 1 acoustic tag survival
%estimate for the Lower Columbia River (below Bonneville Dam)
%We assume a binomial distribution for the survival estimates,
%defined as B(S,N*), where S is the estimated survival proportion,
%and N* is the number of fish released (decreased from N to give the
%variance on the survival estimate reported)

%MATLAB Version 7.4.0.287 (R2007a)
```

FRASER RIVER CHINOOK

```
clear all
%survival estimates calculated using the CJS method using Program MARK
FR_Ch_surv = [ .30154, .16864, .23039, .02041, .32154, .31480];%Mike's
data
FR_Ch_SE = [ .20611, .11710, .05447, .03567, .20673, .06658];
%N_star is a calculated number of fish released that results in the
variance
% (standard error) calculated by the CJS method
FR_Ch_N_star = round((FR_Ch_surv.*(1-FR_Ch_surv))./FR_Ch_SE.^2);
V_FR_Ch = zeros(1,10000);
y_FR_Ch = zeros(10000,6);
for kk=1:10000
    for ii=1:length(FR_Ch_surv)
        %for each individual group (ii), generate a random survival
estimate
        %from a binomial distribution B(FR_Ch_surv(ii),FR_Ch_star(ii))
        y_FR_Ch(kk,ii) = randraw('binom', [FR_Ch_N_star(ii),
FR_Ch_surv(ii)], 1)/FR_Ch_N_star(ii);
        %for a set (kk) of six random generated survival estimates,
calculate
        %the average across the six stocks
```

```
        V_FR_Ch(kk)=mean(y_FR_Ch(kk,:));
    end
end
hist(V_FR_Ch,20);h = findobj(gca,'Type','patch');set(h,'FaceColor',[.8
.8 .8]);
title('\bfFraser River Chinook','fontsize',9);
disp('The mean value for FRASER RIVER CHINOOK survival estimates is
');disp(mean(V_FR_Ch))
S=sort(V_FR_Ch);
disp('Lower 95% CI is ');disp(S(250))
disp('Upper 95% CI is ');disp(S(9750))
axis([0 1 0 1.05*max(hist(V_FR_Ch,20))]);
The mean value for FRASER RIVER CHINOOK survival estimates is
    0.2263

Lower 95% CI is
    0.1260

Upper 95% CI is
    0.3388
```

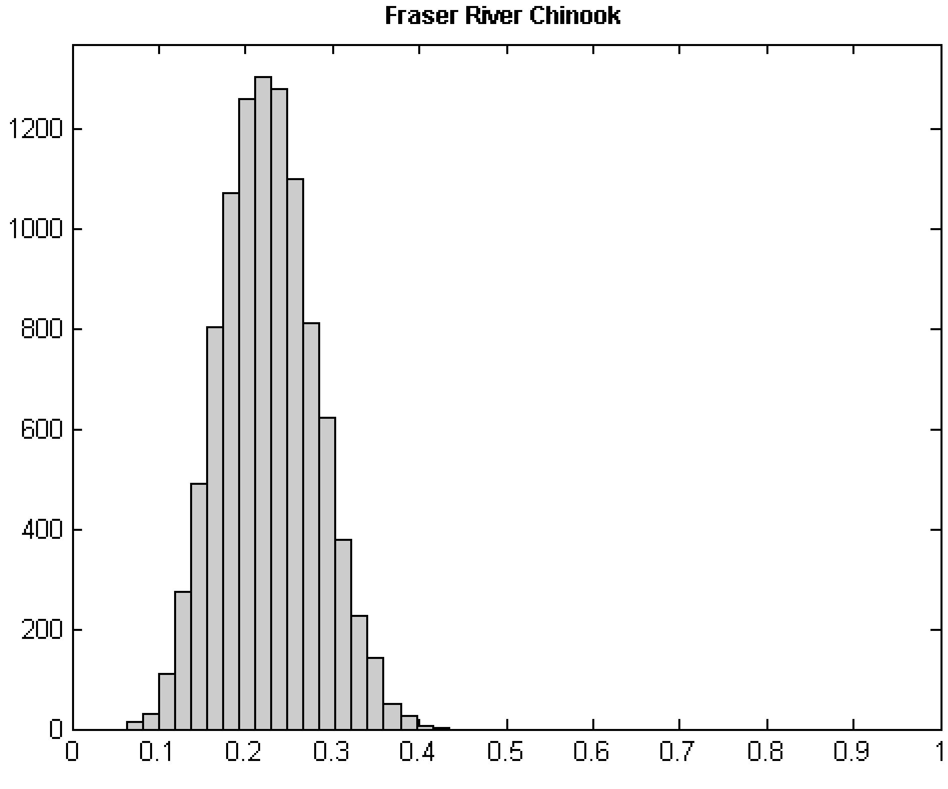

SNAKE RIVER CHINOOK IMPOUNDED

```
%PIT tags survival estimates (NOAA)
CR_Ch_Upper_surv = [.524, .452, .266, .551, .528, .353, .530, .612];
%standard errors for the PIT tags survival estimates (NOAA)
CR_Upper_SE = [.043, .087, .015, .057, .023, .045, .063, .016];
%N_star is calculated to reflect the reported standard errors (NOAA)
CR_Ch_Upper_N_star = round((CR_Ch_Upper_surv.*(1-
CR_Ch_Upper_surv))./CR_Upper_SE.^2);
V_CR_Ch_Upper = zeros(1,10000);
y_CR_Ch_Upper = zeros(10000,8);
for kk=1:10000
    for ii=1:length(CR_Ch_Upper_surv)
        y_CR_Ch_Upper(kk,ii) = randraw('binom',
[CR_Ch_Upper_N_star(ii), CR_Ch_Upper_surv(ii)],
1)/CR_Ch_Upper_N_star(ii);
        V_CR_Ch_Upper(kk)=mean(y_CR_Ch_Upper(kk,:));
    end
end
hist(V_CR_Ch_Upper,20);h =
findobj(gca,'Type','patch');set(h,'FaceColor',[.8 .8 .8]);
title('\bfSnake River Chinook Impounded','fontsize',9);
disp('The mean value for SNAKE RIVER CHINOOK IMPOUNDED survival
estimates is ');disp(mean(V_CR_Ch_Upper))
S=sort(V_CR_Ch_Upper);
disp('Lower 95% CI is ');disp(S(250))
disp('Upper 95% CI is ');disp(S(9750))
axis([0 1 0 1.05*max(hist(V_CR_Ch_Upper,20))]);
The mean value for SNAKE RIVER CHINOOK IMPOUNDED survival estimates is
    0.4770

Lower 95% CI is
    0.4431

Upper 95% CI is
    0.5107
```

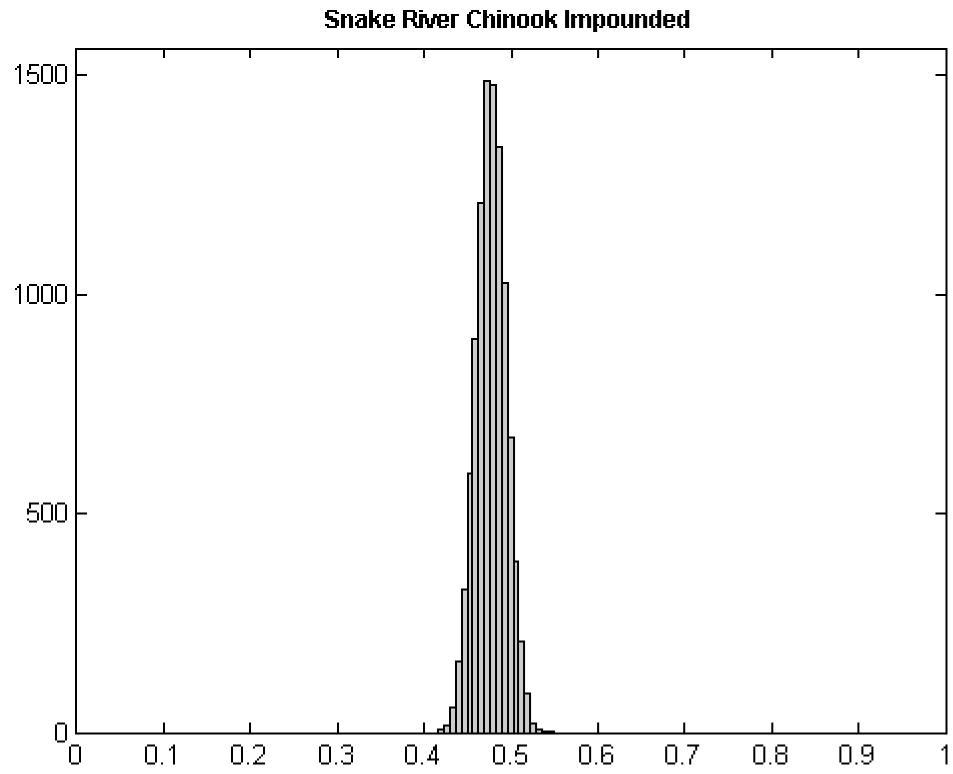

#1SNAKE RIVER CHINOOK IMPOUNDED comparison with FRASER RIVER CHINOOK

```
%differences between pairs (CR avg. surv - FR avg. surv)
V_diff_1 = V_CR_Ch_Upper-V_FR_Ch;
hist(V_diff_1,50);h = findobj(gca,'Type','patch');set(h,'FaceColor',[.8
.8 .8]);
title('\bfDifferences between Snake R Chinook Impounded and
FR','fontsize',9);
S=sort(V_diff_1);
disp('Lower 95% CI is ');disp(S(250))
disp('Upper 95% CI is ');disp(S(9750))
axis([-0.1  max(S)+0.1  0  1.05*max(hist(V_diff_1,50))]);
Lower 95% CI is
    0.1350

Upper 95% CI is
    0.3551
```

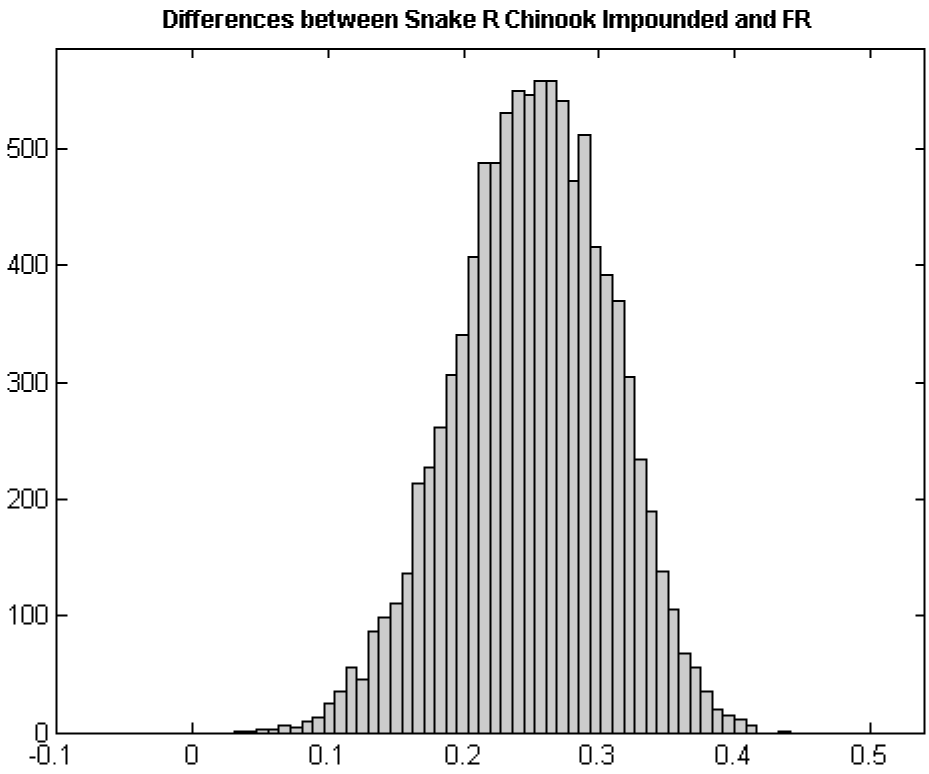

Differences between Snake R Chinook Impounded and FR
500
400
300
200
100
0
-0.1
0
0.1
0.2
0.3
0.4
0.5

SNAKE RIVER CHINOOK UNIMPOUNDED

```
%C. Schreck's 2004 acoustic tags estimated survival
CR_Ch_Lower_surv = [.636];
%number of fish released (C. Schreck)
CR_Ch_Lower_N = [763];
V_CR_Ch_Lower = zeros(1,10000);
y_CR_Ch_Lower = zeros(10000,1);
for kk=1:10000
    for ii=1:length(CR_Ch_Lower_surv)
        y_CR_Ch_Lower(kk,ii) = randraw('binom', [CR_Ch_Lower_N(ii),
CR_Ch_Lower_surv(ii)], 1)/CR_Ch_Lower_N(ii);
        V_CR_Ch_Lower(kk)=mean(y_CR_Ch_Lower(kk,:));
    end
end
hist(V_CR_Ch_Lower,20);h =
findobj(gca,'Type','patch');set(h,'FaceColor',[.8 .8 .8]);
title('\bfSnake River Chinook Unimpounded','fontsize',9);
disp('The mean value for SNAKE RIVER CHINOOK UNIMPOUNDED survival
estimates is ');disp(mean(V_CR_Ch_Lower))
S=sort(V_CR_Ch_Lower);
disp('Lower 95% CI is ');disp(S(250))
disp('Lower 95% CI is ');disp(S(9750))
axis([0 1 0 1.05*max(hist(V_CR_Ch_Lower,20))]);
The mean value for SNAKE RIVER CHINOOK UNIMPOUNDED survival estimates
is
    0.6362

Lower 95% CI is
    0.6016

Lower 95% CI is
    0.6697
```

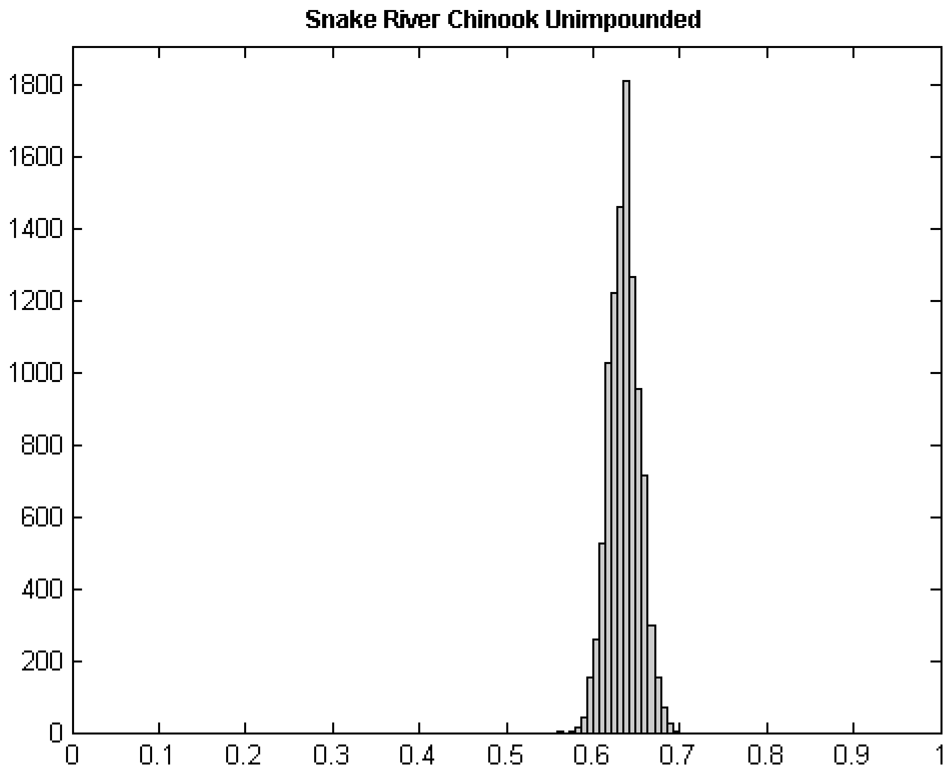
Snake River Chinook Unimpounded
1800
1600
1400
1200
1000
800
600
400
200
0
0
0.1
0.2
0.3
0.4
0.5
0.6
0.7
0.8
0.9
1

#2SNAKE RIVER CHINOOK UNIMPOUNDED comparison with FRASER RIVER CHINOOK

```
%differences between pairs (CR avg. surv - FR avg. surv)
V_diff_2 = V_CR_Ch_Lower-V_FR_Ch;
hist(V_diff_2,50);h = findobj(gca,'Type','patch');set(h,'FaceColor',[.8
.8 .8]);
title('\bfDifferences between Snake R Chinook Unimpounded and
FR','fontsize',9);
S=sort(V_diff_2);
disp('Lower 95% CI is ');disp(S(250))
disp('Upper 95% CI is ');disp(S(9750))
if min(S)>0; axis([-0.1  max(S)+0.1  0
1.05*max(hist(V_diff_2,50))]);end
if max(S)<0; axis([min(S)-0.1  0.1  0
1.05*max(hist(V_diff_2,50))]);end
Lower 95% CI is
    0.2957
Upper 95% CI is
    0.5155
```

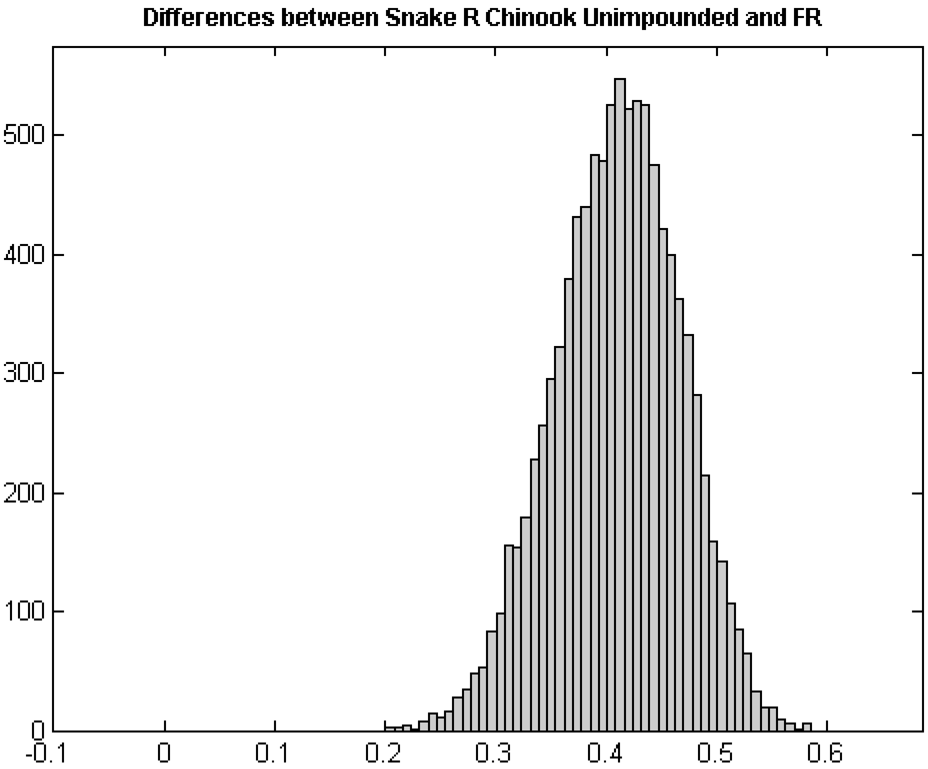

COLUMBIA-SNAKE RIVER-ENTIRE CHINOOK 2006

```
%for this part we have to consider 1) the Kintama results for 2006, and
2)
%the synthetic result for 2004 that was obtained by multiplying the PIT
tag
%results (above Bonneville) with the acoustic results (below
Bonneville)
CR_Ch_Entire_surv = [.27523];%Kintama 2006 results
CR_Ch_Entire_SE = [.06878];
CR_Ch_Entire_N_star = round((CR_Ch_Entire_surv.*(1-
CR_Ch_Entire_surv))./CR_Ch_Entire_SE.^2);
V_CR_Ch_Entire_2006 = zeros(1,10000);
y_CR_Ch_Entire_2006 = zeros(10000,1);
for kk=1:10000
    for ii=1:length(CR_Ch_Entire_surv)
        y_CR_Ch_Entire_2006(kk,ii) = randraw('binom',
[CR_Ch_Entire_N_star(ii), CR_Ch_Entire_surv(ii)],
1)/CR_Ch_Entire_N_star(ii);
        V_CR_Ch_Entire_2006(kk)=mean(y_CR_Ch_Entire_2006(kk,:));
    end
end
hist(V_CR_Ch_Entire_2006,20);h =
findobj(gca,'Type','patch');set(h,'FaceColor',[.8 .8 .8]);
title('\bfEntire Columbia-Snake River Chinook - 2006
results','fontsize',9);
disp('The mean value for COLUMBIA-SNAKE RIVER-ENTIRE CHINOOK 2006
survival estimates is ');disp(mean(V_CR_Ch_Entire_2006))
S=sort(V_CR_Ch_Entire_2006);
disp('Lower 95% CI is ');disp(S(250))
disp('Upper 95% CI is ');disp(S(9750))
axis([0 1 0 1.05*max(hist(V_CR_Ch_Entire_2006,20))]);
The mean value for COLUMBIA-SNAKE RIVER-ENTIRE CHINOOK 2006 survival
estimates is
    0.2748

Lower 95% CI is
    0.1429

Upper 95% CI is
    0.4048
```

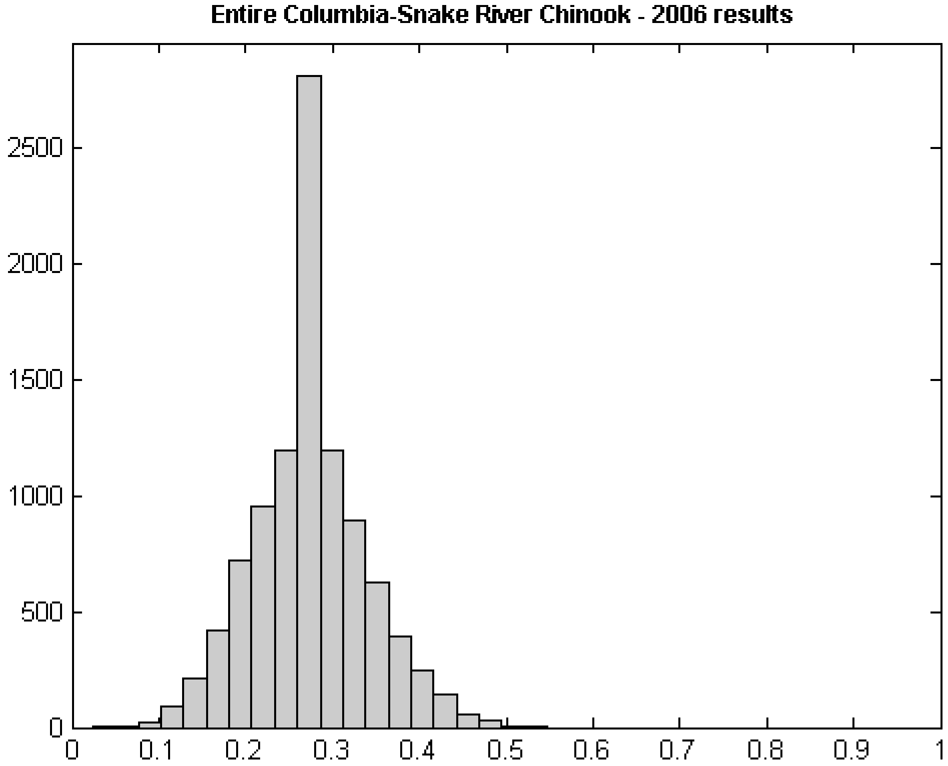

COLUMBIA-SNAKE RIVER-ENTIRE CHINOOK 2004

```
%the 2004 synthetic results
V_CR_Ch_Entire_2004=y_CR_Ch_Lower.*y_CR_Ch_Upper(:,6);
hist(V_CR_Ch_Entire_2004,20);h =
findobj(gca,'Type','patch');set(h,'FaceColor',[.8 .8 .8]);
title('\bfEntire Columbia-Snake River Chinook - 2004
results','fontsize',9);
disp('The mean value for COLUMBIA-SNAKE RIVER-ENTIRE CHINOOK 2004
survival estimates is ');disp(mean(V_CR_Ch_Entire_2004))
S=sort(V_CR_Ch_Entire_2004);
disp('Lower 95% CI is ');disp(S(250))
disp('Upper 95% CI is ');disp(S(9750))
axis([0 1 0 1.05*max(hist(V_CR_Ch_Entire_2004,20))]);
The mean value for COLUMBIA-SNAKE RIVER-ENTIRE CHINOOK 2004 survival
estimates is
    0.2242

Lower 95% CI is
    0.1682

Upper 95% CI is
    0.2818
```

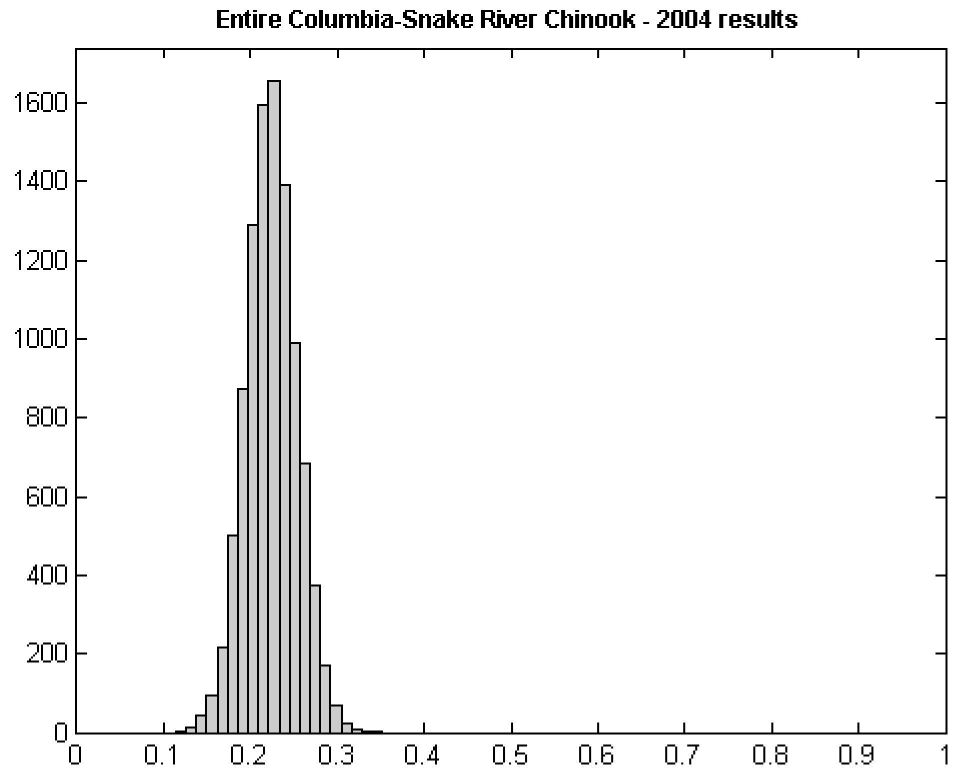

COLUMBIA-SNAKE RIVER-ENTIRE CHINOOK (2006 and 2004 combined)

```
V_CR_Ch_Entire=zeros(1,10000);
for kk=1:10000

V_CR_Ch_Entire(kk)=mean([V_CR_Ch_Entire_2006(kk),V_CR_Ch_Entire_2004(kk
)]);
end
```

```
hist(V_CR_Ch_Entire,20);h =
findobj(gca,'Type','patch');set(h,'FaceColor',[.8 .8 .8]);
title('\bfEntire Columbia-Snake River Chinook','fontsize',9);
disp('The mean value for COLUMBIA-SNAKE RIVER-ENTIRE CHINOOK survival
estimates is ');disp(mean(V_CR_Ch_Entire))
S=sort(V_CR_Ch_Entire);
disp('Lower 95% CI is ');disp(S(250))
disp('Upper 95% CI is ');disp(S(9750))
axis([0 1 0 1.05*max(hist(V_CR_Ch_Entire,20))]);
The mean value for COLUMBIA-SNAKE RIVER-ENTIRE CHINOOK survival
estimates is
    0.2495

Lower 95% CI is
    0.1796

Upper 95% CI is
    0.3241
```

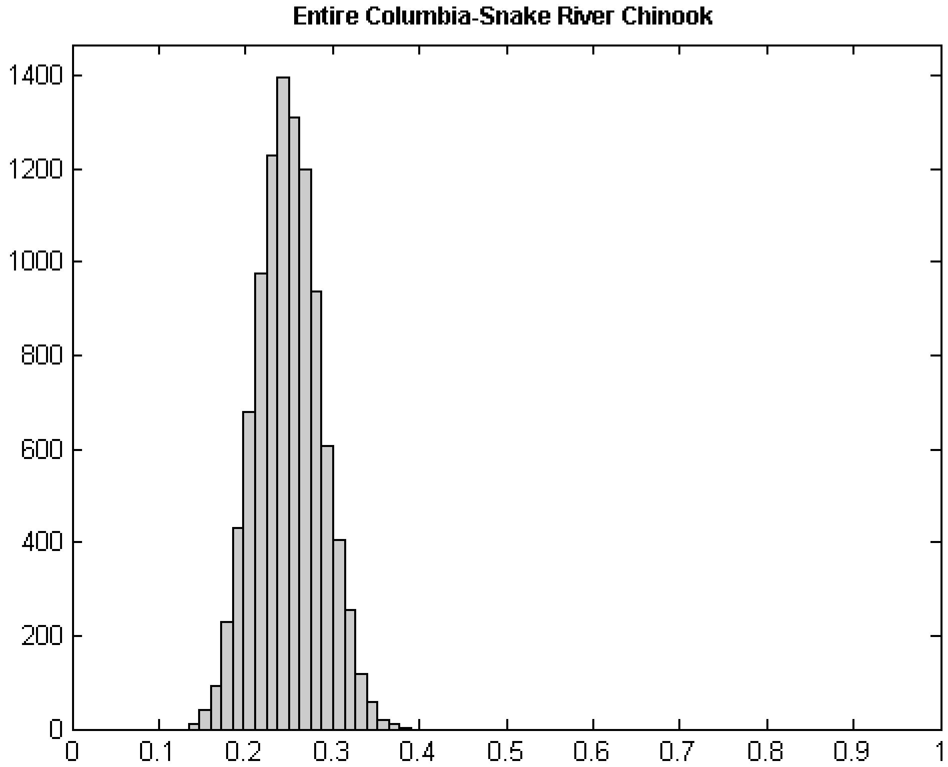

#3COLUMBIA-SNAKE RIVER-ENTIRE CHINOOK comparison with FRASER RIVER CHINOOK

```
%differences between pairs (CR avg. surv - FR avg. surv)
V_diff_3 = V_CR_Ch_Entire-V_FR_Ch;
hist(V_diff_3,50);h = findobj(gca,'Type','patch');set(h,'FaceColor',[.8
.8 .8]);
title('\bfDifferences between Entire Columbis-Snake R and FR
','fontsize',9);
S=sort(V_diff_3);
disp('Lower 95% CI is ');disp(S(250))
disp('Upper 95% CI is ');disp(S(9750))
if min(S)>0; axis([-0.1  max(S)+0.1  0
1.05*max(hist(V_diff_3,50))]);end
if max(S)<0; axis([min(S)-0.1  0.1  0
1.05*max(hist(V_diff_3,50))]);end
Lower 95% CI is
   -0.1079

Upper 95% CI is
    0.1486
```

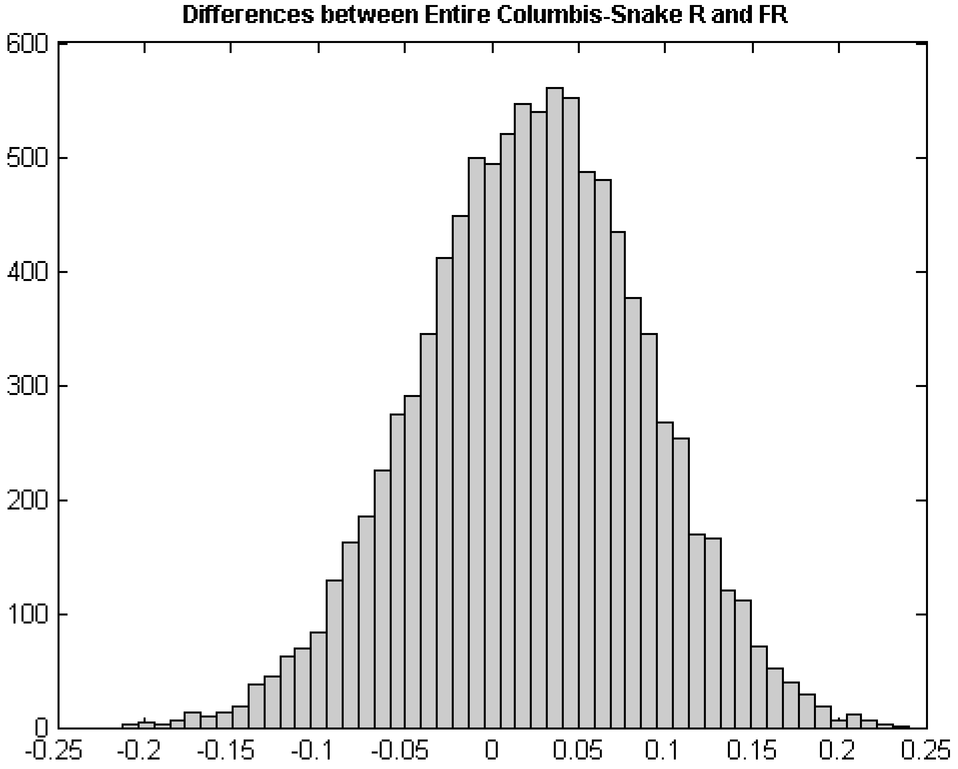

FRASER RIVER CHINOOK scaled by distance (per 100 km)

```
FR_Ch_dist = [330.8,330.8,395.22,367.93,330.6,355.02];%Kintama results
V_FR_Ch_dist = zeros(1,10000);
y_FR_Ch_dist = zeros(10000,6);
for kk=1:10000
    for ii=1:length(FR_Ch_surv)
```

```
        y_FR_Ch_dist(kk,ii) = (y_FR_Ch(kk,ii))^(100/FR_Ch_dist(ii));
        V_FR_Ch_dist(kk)=mean(y_FR_Ch_dist(kk,:));
    end
end
hist(V_FR_Ch_dist,20);h =
findobj(gca,'Type','patch');set(h,'FaceColor',[.8 .8 .8]);
title('\bfFraser River Chinook scaled by distance (per 100
km)','fontsize',9);
disp('The mean value for FRASER RIVER CHINOOK survival estimates scaled
by distance (per 100 km) is ');disp(mean(V_FR_Ch_dist))
S=sort(V_FR_Ch_dist);
disp('Lower 95% CI is ');disp(S(250))
disp('Upper 95% CI is ');disp(S(9750))
axis([0 1 0 1.05*max(hist(V_FR_Ch_dist,20))]);
The mean value for FRASER RIVER CHINOOK survival estimates scaled by
distance (per 100 km) is
    0.5447

Lower 95% CI is
    0.3457

Upper 95% CI is
    0.6882
```

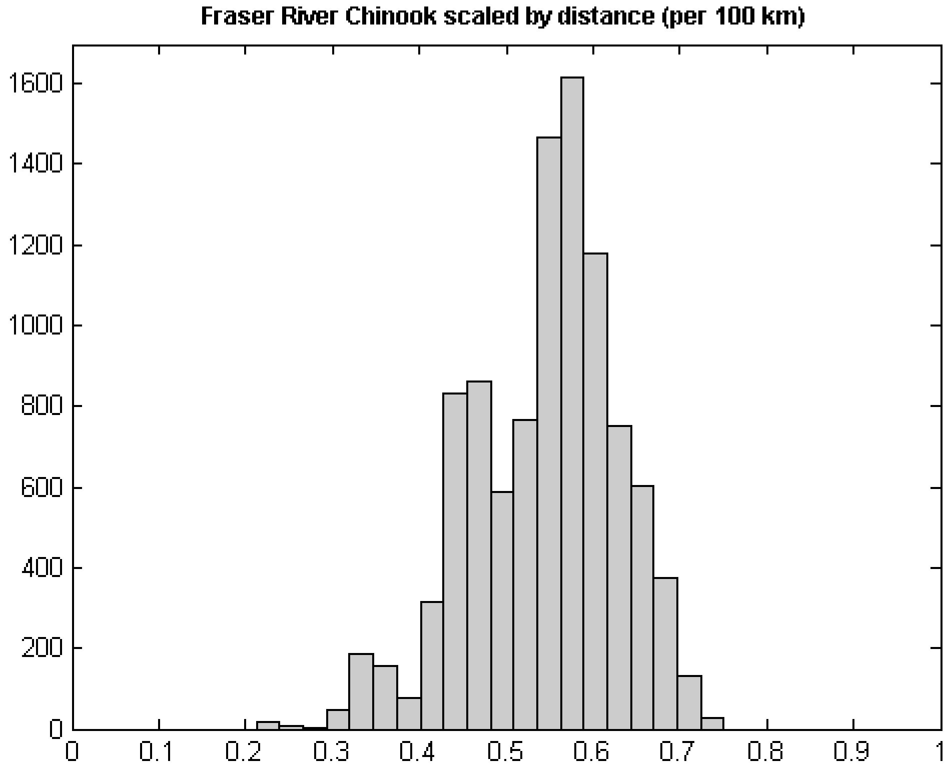

SNAKE RIVER CHINOOK IMPOUNDED scaled by distance (per 100 km)

```
CR_Ch_Upper_dist = [506];%PIT results
V_CR_Ch_Upper_dist = zeros(1,10000);
y_CR_Ch_Upper_dist = zeros(10000,8);
for kk=1:10000
    for ii=1:length(CR_Ch_Upper_surv)
        y_CR_Ch_Upper_dist(kk,ii) =
(y_CR_Ch_Upper(kk,ii))^(100/CR_Ch_Upper_dist);
        V_CR_Ch_Upper_dist(kk)=mean(y_CR_Ch_Upper_dist(kk,:));
    end
end
hist(V_CR_Ch_Upper_dist,20); h =
findobj(gca,'Type','patch');set(h,'FaceColor',[.8 .8 .8]);
title('\bfSnake River Chinook Impounded scaled by distance (per 100
km)','fontsize',9);
disp('The mean value for SNAKE RIVER CHINOOK IMPOUNDED survival
estimates scaled by distance (per 100 km) is
');disp(mean(V_CR_Ch_Upper_dist))
S=sort(V_CR_Ch_Upper_dist);
disp('Lower 95% CI is ');disp(S(250))
disp('Upper 95% CI is ');disp(S(9750))
axis([0 1 0 1.05*max(hist(V_CR_Ch_Upper_dist,20))]);
The mean value for SNAKE RIVER CHINOOK IMPOUNDED survival estimates
scaled by distance (per 100 km) is
    0.8590

Lower 95% CI is
    0.8458

Upper 95% CI is
    0.8710
```

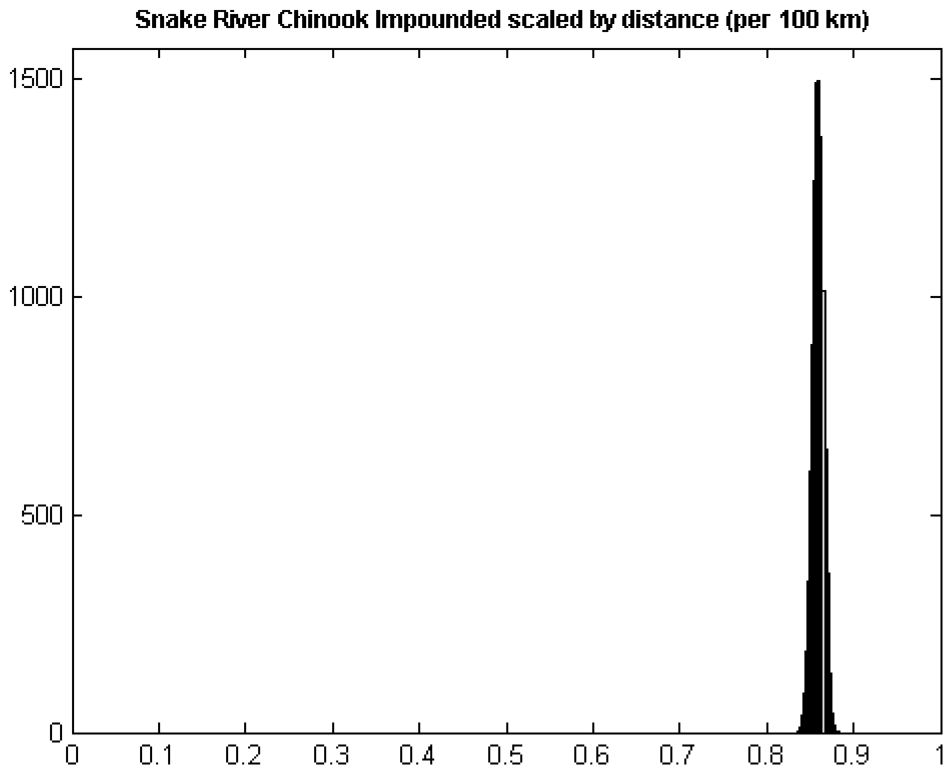

#4SNAKE RIVER CHINOOK IMPOUNDED comparison with FRASER CHINOOK scaled by distance

```
%differences between pairs (CR avg. surv - FR avg. surv)
V_diff_4 = V_CR_Ch_Upper_dist-V_FR_Ch_dist;
hist(V_diff_4,50);h = findobj(gca,'Type','patch');set(h,'FaceColor',[.8
.8 .8]);
title('\bfDifferences between SNAKE R CHINOOK IMPOUNDED and FR scaled
by distance (per 100 km)','fontsize',9);
S=sort(V_diff_4);
disp('Lower 95% CI is ');disp(S(250))
disp('Upper 95% CI is ');disp(S(9750))
if min(S)>0; axis([-0.1  max(S)+0.1  0
1.05*max(hist(V_diff_4,50))]);end
if max(S)<0; axis([min(S)-0.1  0.1  0
1.05*max(hist(V_diff_4,50))]);end
Lower 95% CI is
    0.1703
```

```
Upper 95% CI is
    0.5115
```

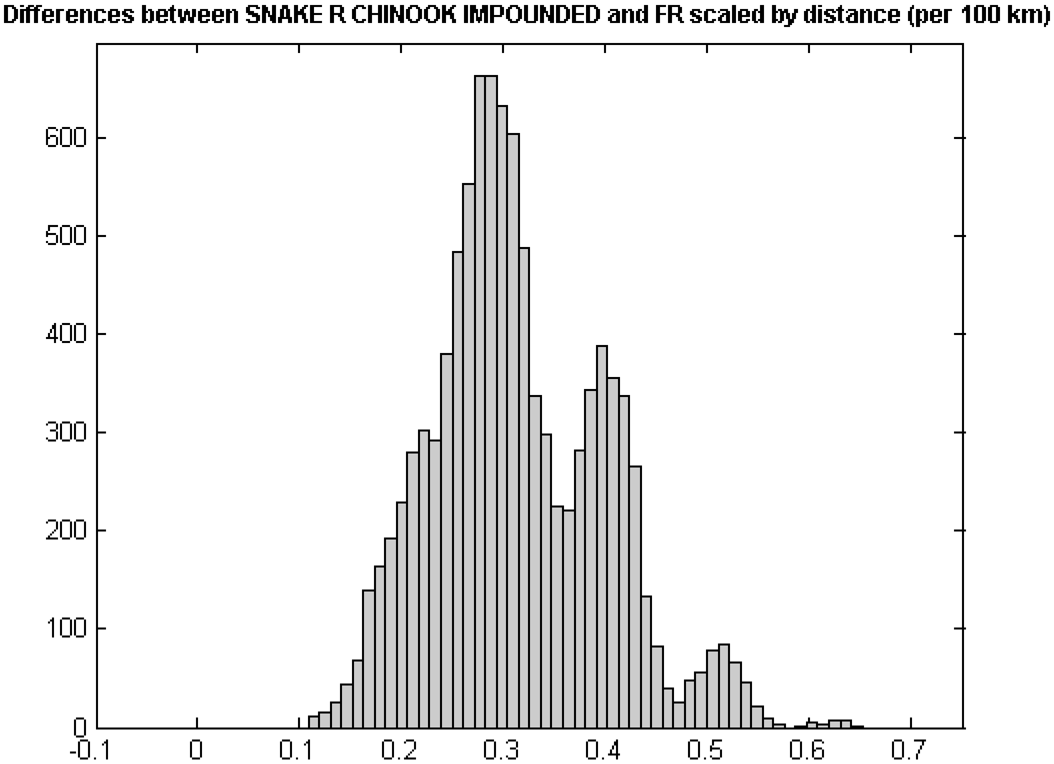

SNAKE RIVER CHINOOK UNIMPOUNDED scaled by distance (per 100 km)

```
CR_Ch_Lower_dist = [212];%C. Schreck's results
V_CR_Ch_Lower_dist = zeros(1,10000);
y_CR_Ch_Lower_dist = zeros(10000,1);
for kk=1:10000
    for ii=1:length(CR_Ch_Lower_surv)
        y_CR_Ch_Lower_dist(kk,ii) =
(y_CR_Ch_Lower(kk,ii))^(100/CR_Ch_Lower_dist);
        V_CR_Ch_Lower_dist(kk)=mean(y_CR_Ch_Lower_dist(kk,:));
    end
end
hist(V_CR_Ch_Lower_dist,20); h =
findobj(gca,'Type','patch');set(h,'FaceColor',[.8 .8 .8]);
title('\bfSnake River Chinook Unimpounded scaled by distance (per 100
km)','fontsize',9);
disp('The mean value for SNAKE RIVER CHINOOK UNIMPOUNDED survival
estimates scaled by distance (per 100 km) is
');disp(mean(V_CR_Ch_Lower_dist))
S=sort(V_CR_Ch_Lower_dist);
disp('Lower 95% CI is ');disp(S(250))
disp('Upper 95% CI is ');disp(S(9750))
axis([0 1 0 1.05*max(hist(V_CR_Ch_Lower_dist,20))]);
The mean value for SNAKE RIVER CHINOOK UNIMPOUNDED survival estimates
scaled by distance (per 100 km) is
    0.8078

Lower 95% CI is
    0.7868

Upper 95% CI is
    0.8277
```

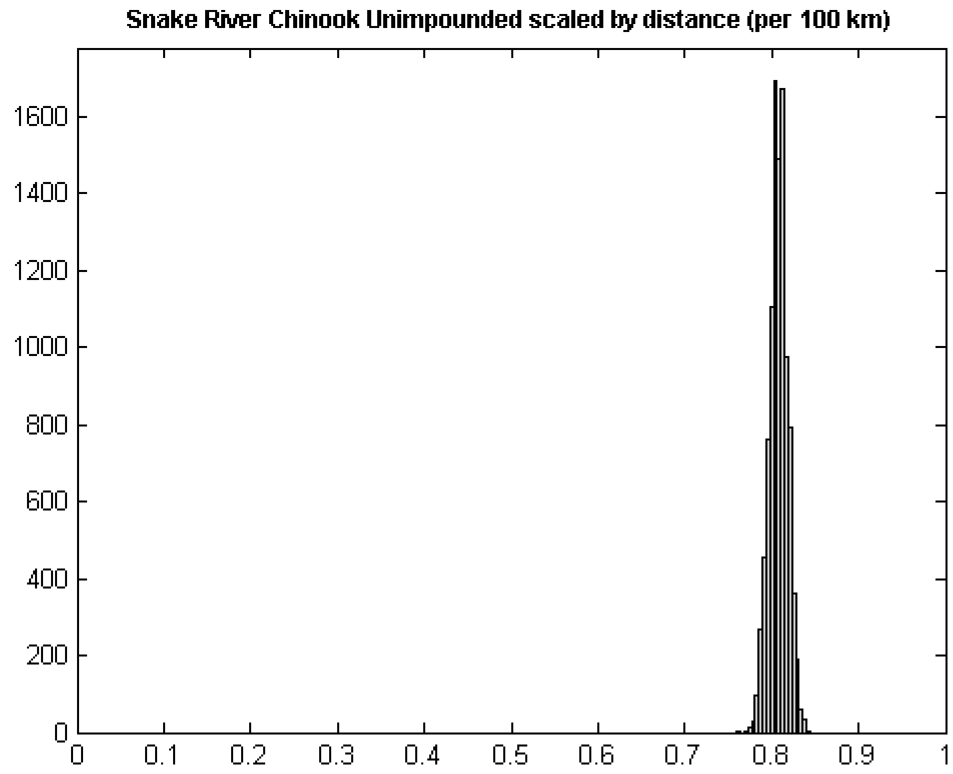

#5SNAKE RIVER CHINOOK UNIMPOUNDED comparison with FRASER CHINOOK scaled by distance

```
%differences between pairs (CR avg. surv - FR avg. surv)
V_diff_5 = V_CR_Ch_Lower_dist-V_FR_Ch_dist;
hist(V_diff_5,50);h = findobj(gca,'Type','patch');set(h,'FaceColor',[.8
.8 .8]);
title('\bfDifferences between Snake R Chinook Unimpounded and FR scaled
by distance (per 100 km)','fontsize',9);
S=sort(V_diff_5);
disp('Lower 95% CI is ');disp(S(250))
disp('Upper 95% CI is ');disp(S(9750))
Lo_V_diff_5 = S(250);Hi_V_diff_5 = S(9750);%axis([-0.3 0.3 0 2500]);
if min(S)>0; axis([-0.1  max(S)+0.1  0
1.05*max(hist(V_diff_5,50))]);end
if max(S)<0; axis([min(S)-0.1  0.1  0
1.05*max(hist(V_diff_5,50))]);end
Lower 95% CI is
```

```
    0.1167

Upper 95% CI is
    0.4601
```

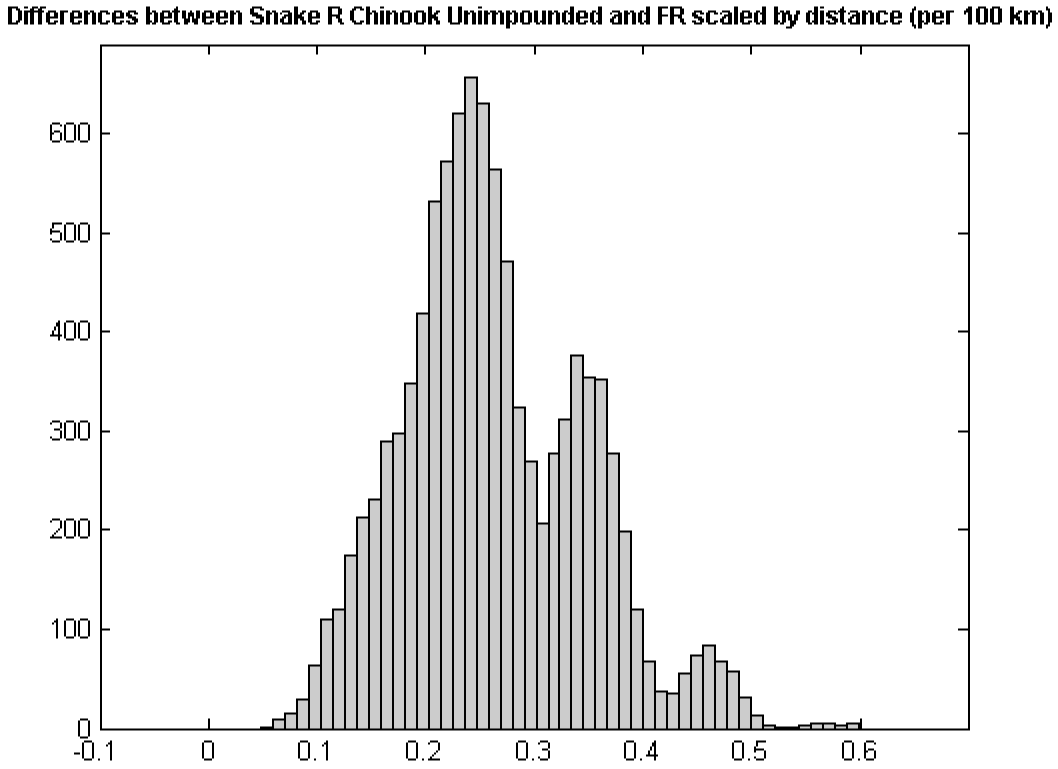

COLUMBIA-SNAKE ENTIRE CHINOOK scaled by distance (per 100 km) 2006

```
CR_Ch_Entire_dist = [910];%Kintama results
V_CR_Ch_Entire_dist_2006 = zeros(1,10000);
y_CR_Ch_Entire_dist = zeros(10000,1);
for kk=1:10000
    for ii=1:length(CR_Ch_Entire_surv)
        y_CR_Ch_Entire_dist(kk,ii) =
(y_CR_Ch_Entire_2006(kk,ii))^(100/CR_Ch_Entire_dist);
        V_CR_Ch_Entire_dist_2006(kk)=mean(y_CR_Ch_Entire_dist(kk,:));
    end
end
hist(V_CR_Ch_Entire_dist_2006,20);h =
findobj(gca,'Type','patch');set(h,'FaceColor',[.8 .8 .8]);
title('\bfEntire Columbia-Snake R Chinook scaled by distance (per 100
km) - 2006 results','fontsize',9);
disp('The mean value for COLUMBIA- SNAKE ENTIRE CHINOOK survival
estimates scaled by distance (per 100 km) 2006 is
');disp(mean(V_CR_Ch_Entire_dist_2006))
S=sort(V_CR_Ch_Entire_dist_2006);
disp('Lower 95% CI is ');disp(S(250))
disp('Upper 95% CI is ');disp(S(9750))
axis([0 1 0 1.05*max(hist(V_CR_Ch_Entire_dist_2006,20))]);
The mean value for COLUMBIA- SNAKE ENTIRE CHINOOK survival estimates
scaled by distance (per 100 km) 2006 is
    0.8649

Lower 95% CI is
    0.8075

Upper 95% CI is
    0.9054
```

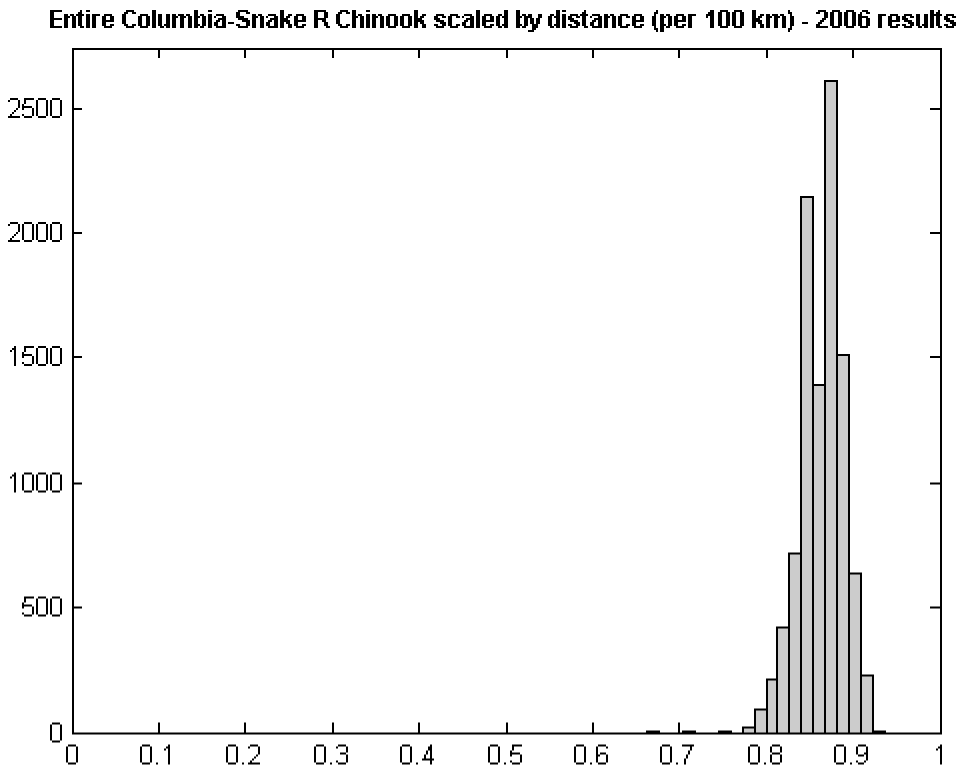

COLUMBIA-SNAKE ENTIRE CHINOOK scaled by distance (per 100 km) 2004

```
%the 2004 synthetic results
V_CR_Ch_Entire_dist_2004=(y_CR_Ch_Lower.*y_CR_Ch_Upper(:,6)).^(100/(506
+212));
hist(V_CR_Ch_Entire_dist_2004,20);h =
findobj(gca,'Type','patch');set(h,'FaceColor',[.8 .8 .8]);
title('\bfEntire Columbia-Snake River Chinook scaled by distance (per
100 km) - 2004 results','fontsize',9);
disp('The mean value for COLUMBIA-SNAKE ENTIRE CHINOOK survival
estimates scaled by distance (per 100 km) 2004 is
');disp(mean(V_CR_Ch_Entire_dist_2004))
S=sort(V_CR_Ch_Entire_dist_2004);
disp('Lower 95% CI is ');disp(S(250))
disp('Upper 95% CI is ');disp(S(9750))
axis([0 1 0 1.05*max(hist(V_CR_Ch_Entire_dist_2004,20))]);
```

```
The mean value for COLUMBIA-SNAKE ENTIRE CHINOOK survival estimates
scaled by distance (per 100 km) 2004 is
    0.8111

Lower 95% CI is
    0.7801

Upper 95% CI is
    0.8383
```

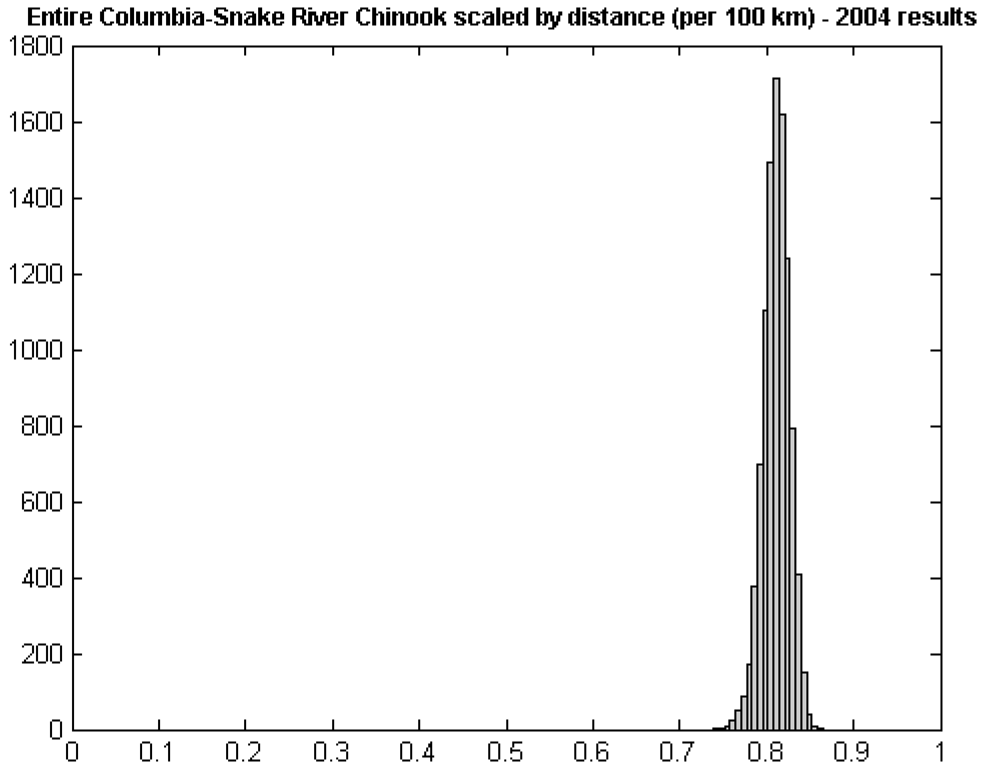

COLUMBIA-SNAKE ENTIRE CHINOOK scaled by distance (per 100 km) (2006 and 2004)

```
V_CR_Ch_Entire_dist = zeros(1,10000);
for kk=1:10000

V_CR_Ch_Entire_dist(kk)=mean([V_CR_Ch_Entire_dist_2006(kk),V_CR_Ch_Enti
re_dist_2004(kk)]);
end
hist(V_CR_Ch_Entire_dist,20);h =
findobj(gca,'Type','patch');set(h,'FaceColor',[.8 .8 .8]);
title('\bfEntire Columbia-Snake River Chinook scaled by distance (per
100 km)','fontsize',9);
disp('The mean value for COLUMBIA-SNAKE ENTIRE CHINOOK survival
estimates scaled by distance (per 100 km) is
');disp(mean(V_CR_Ch_Entire_dist))
S=sort(V_CR_Ch_Entire_dist);
disp('Lower 95% CI is ');disp(S(250))
disp('Upper 95% CI is ');disp(S(9750))
axis([0 1 0 1.05*max(hist(V_CR_Ch_Entire_dist,20))]);
The mean value for COLUMBIA-SNAKE ENTIRE CHINOOK survival estimates
scaled by distance (per 100 km) is
    0.8380

Lower 95% CI is
    0.8073

Upper 95% CI is
    0.8639
```

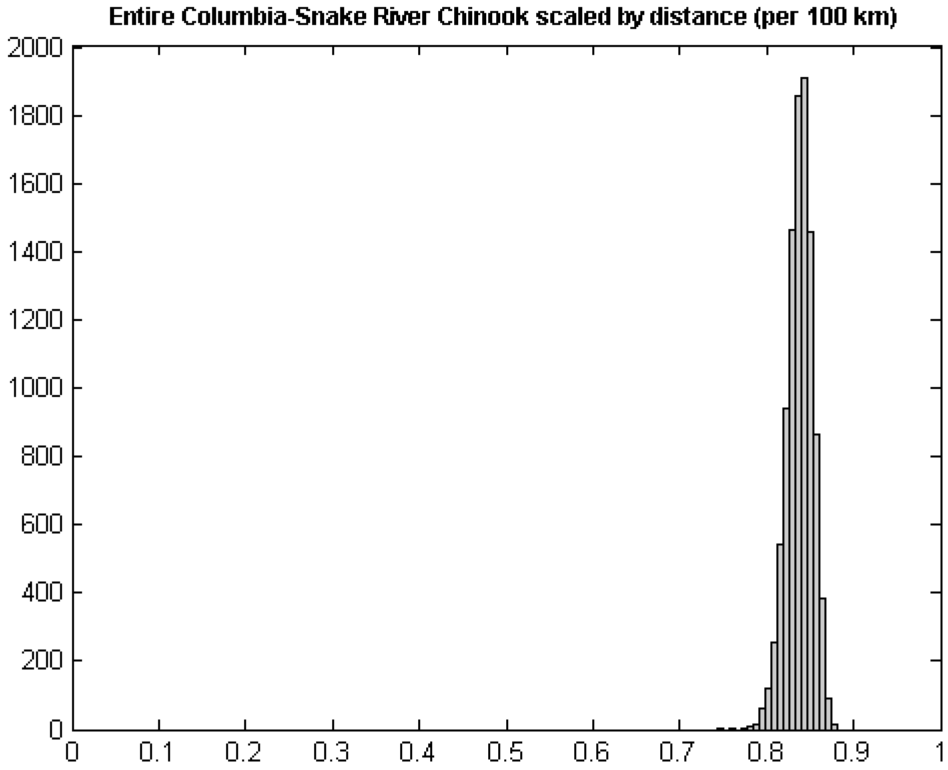

#6COLUMBIA-SNAKE ENTIRE CHINOOK comparison with FRASER CHINOOK scaled by distance (per 100 km)

```
%differences between pairs (CR avg. surv - FR avg. surv)
V_diff_6 = V_CR_Ch_Entire_dist_2006-V_FR_Ch_dist;
hist(V_diff_6,50);h = findobj(gca,'Type','patch');set(h,'FaceColor',[.8
.8 .8]);
title('\bfDifferences between Entire Columbia-Snake R and FR scaled by
distance (per 100 km)','fontsize',9);
S=sort(V_diff_6);
disp('Lower 95% CI is ');disp(S(250))
disp('Upper 95% CI is ');disp(S(9750))
if min(S)>0; axis([-0.1  max(S)+0.1  0
1.05*max(hist(V_diff_6,50))]);end
if max(S)<0; axis([min(S)-0.1  0.1  0
1.05*max(hist(V_diff_6,50))]);end
Lower 95% CI is
    0.1670

Upper 95% CI is
    0.5179
```

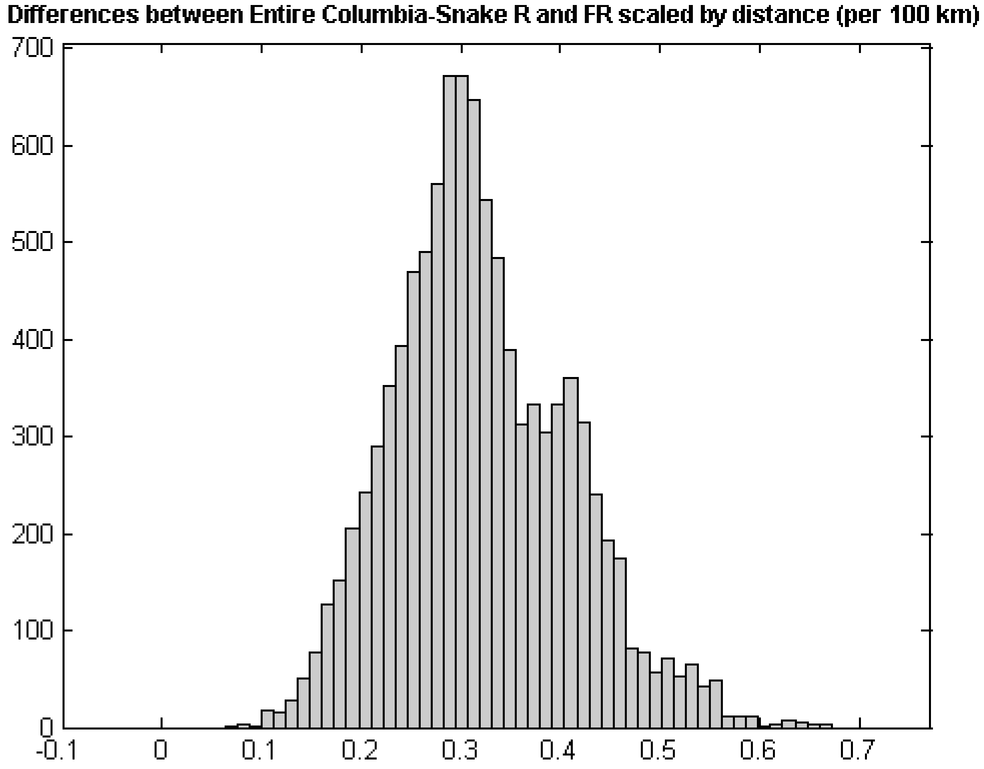

FRASER RIVER CHINOOK scaled by time (per day)

```
%median travel time
FR_Ch_time = [4.13,3.4,13.31,12.23,5.04,19.2];%Kintama results
V_FR_Ch_time = zeros(1,10000);
y_FR_Ch_time = zeros(10000,6);
for kk=1:10000
```

```
    for ii=1:length(FR_Ch_surv)
        y_FR_Ch_time(kk,ii) = (y_FR_Ch(kk,ii))^(1/FR_Ch_time(ii));
        V_FR_Ch_time(kk)=mean(y_FR_Ch_time(kk,:));
    end
end
hist(V_FR_Ch_time,20); h =
findobj(gca,'Type','patch');set(h,'FaceColor',[.8 .8 .8]);
title('\bfFraser River Chinook scaled by time (per day)','fontsize',9);
disp('The mean value for FRASER RIVER CHINOOK survival estimates scaled
by time (per day) is ');disp(mean(V_FR_Ch_time))
S=sort(V_FR_Ch_time);
disp('Lower 95% CI is ');disp(S(250))
disp('Upper 95% CI is ');disp(S(9750))
axis([0 1 0 1.05*max(hist(V_FR_Ch_time,20))]);
The mean value for FRASER RIVER CHINOOK survival estimates scaled by
time (per day) is
    0.6504

Lower 95% CI is
    0.4279

Upper 95% CI is
    0.8296
```

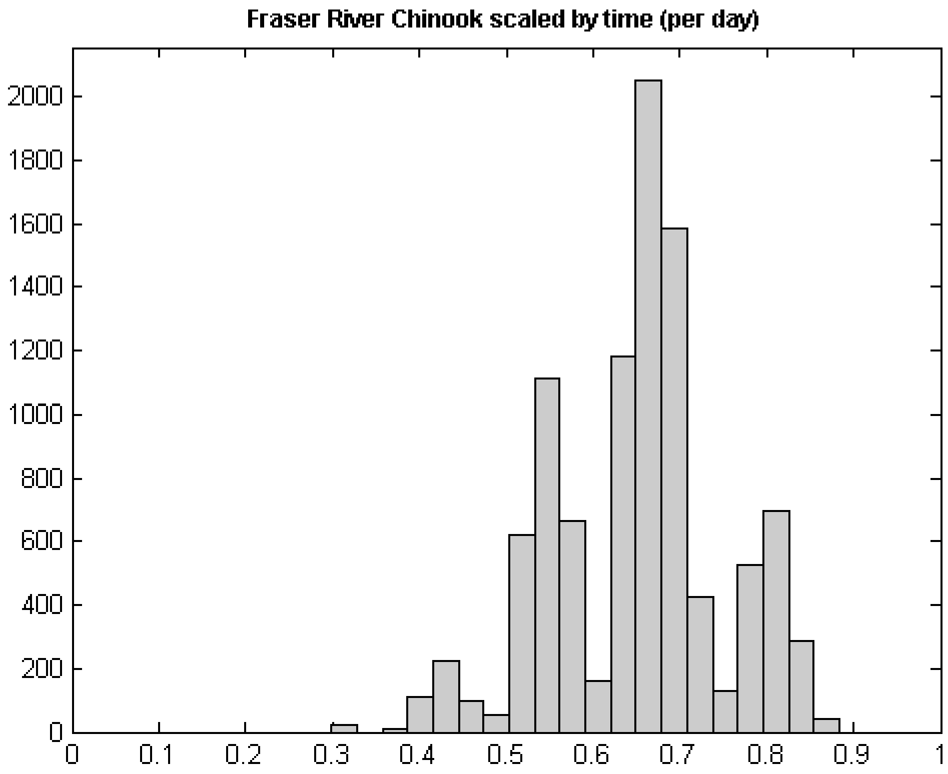

SNAKE RIVER CHINOOK IMPOUNDED scaled by time (per day)

```
%median travel time
CR_Ch_Upper_time = [21.71, 22.88, 40.01, 31.19, 18.24, 21.36, 22.43,
20.31];%PIT results
V_CR_Ch_Upper_time = zeros(1,10000);
y_CR_Ch_Upper_time = zeros(10000,8);
for kk=1:10000
    for ii=1:length(CR_Ch_Upper_surv)
        y_CR_Ch_Upper_time(kk,ii) =
(y_CR_Ch_Upper(kk,ii))^(1/CR_Ch_Upper_time(ii));
        V_CR_Ch_Upper_time(kk)=mean(y_CR_Ch_Upper_time(kk,:));
    end
end
hist(V_CR_Ch_Upper_time,20);h =
findobj(gca,'Type','patch');set(h,'FaceColor',[.8 .8 .8]);
title('\bfSnake River Chinook Impounded scaled by time (per
day)','fontsize',9);
disp('The mean value for SNAKE RIVER CHINOOK IMPOUNDED survival
estimates scaled by time (per day) is ');disp(mean(V_CR_Ch_Upper_time))
S=sort(V_CR_Ch_Upper_time);
disp('Lower 95% CI is ');disp(S(250))
disp('Upper 95% CI is ');disp(S(9750))
axis([0 1 0 1.05*max(hist(V_CR_Ch_Upper_time,20))]);
The mean value for SNAKE RIVER CHINOOK IMPOUNDED survival estimates
scaled by time (per day) is
    0.9687

Lower 95% CI is
    0.9654

Upper 95% CI is
    0.9716
```

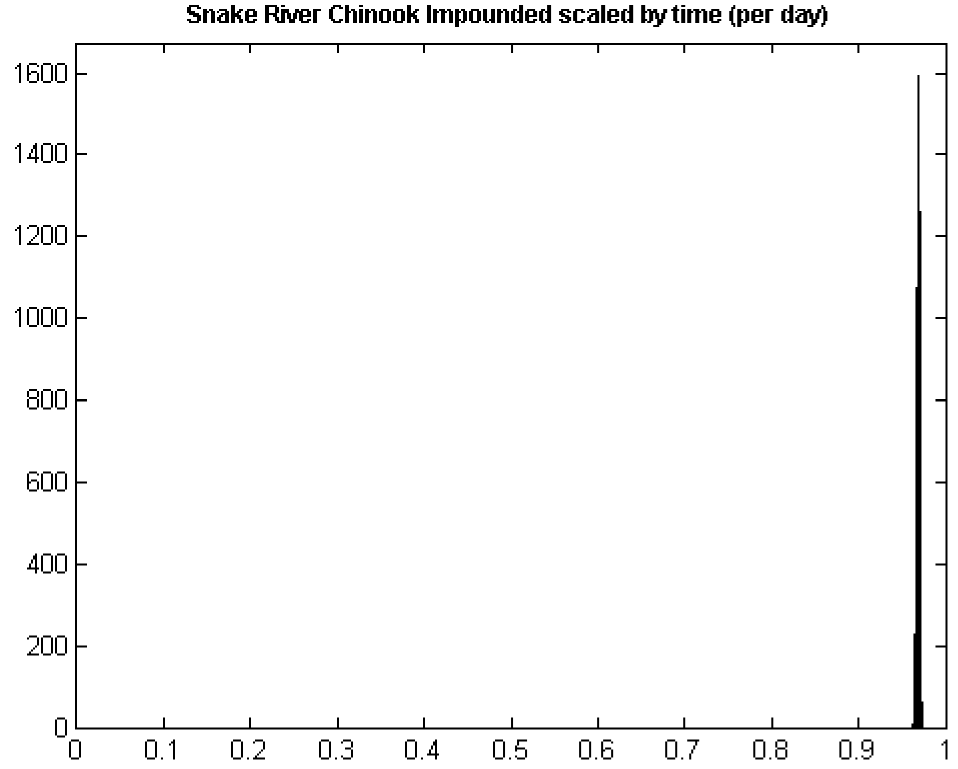

#7SNAKE RIVER CHINOOK IMPOUNDED comparison with FRASER CHINOOK scaled by time (per day)

```
%differences between pairs (CR avg. surv - FR avg. surv)
V_diff_7 = V_CR_Ch_Upper_time-V_FR_Ch_time;
hist(V_diff_7,50);h = findobj(gca,'Type','patch');set(h,'FaceColor',[.8
.8 .8]);
title('\bfDifferences between Snake R Chinook Impounded and FR scaled
by time (per day)','fontsize',9);
S=sort(V_diff_7);
disp('Lower 95% CI is ');disp(S(250))
disp('Upper 95% CI is ');disp(S(9750))
if min(S)>0; axis([-0.1  max(S)+0.1  0
1.05*max(hist(V_diff_7,50))]);end
if max(S)<0; axis([min(S)-0.1  0.1  0
1.05*max(hist(V_diff_7,50))]);end
Lower 95% CI is
    0.1393
```

```
Upper 95% CI is
    0.5411
```

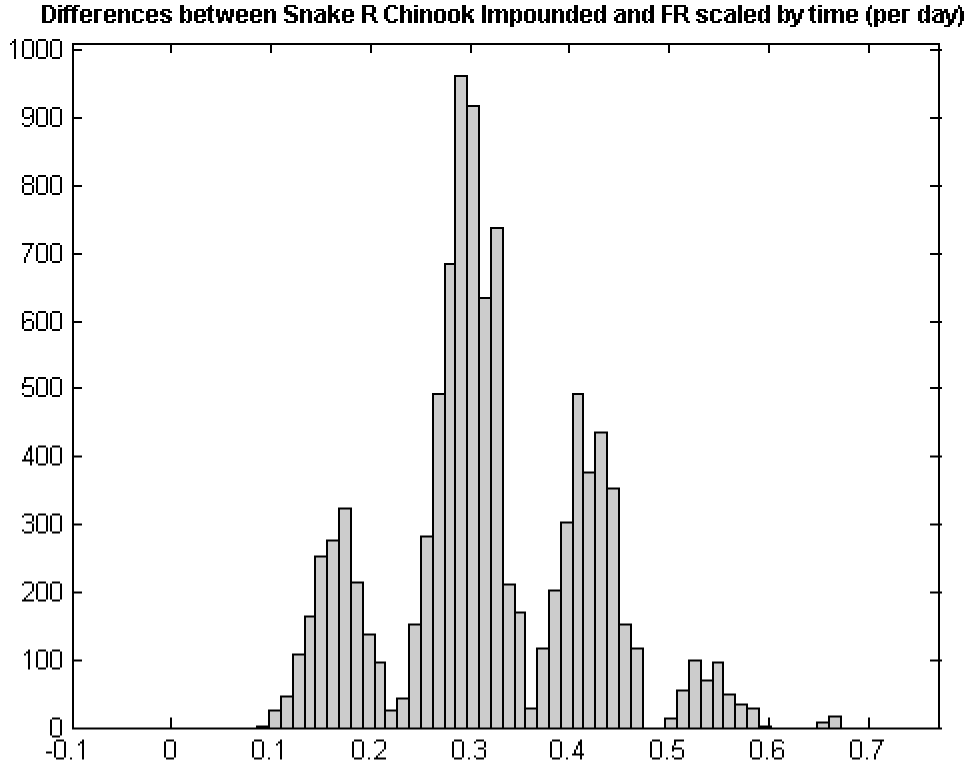

SNAKE RIVER CHINOOK UNIMPOUNDED scaled by time (per day)

```
%median travel time
CR_Ch_Lower_time = [3.42];%Schreck's results
V_CR_Ch_Lower_time = zeros(1,10000);
y_CR_Ch_Lower_time = zeros(10000,1);
for kk=1:10000
    for ii=1:length(CR_Ch_Lower_surv)
        y_CR_Ch_Lower_time(kk,ii) =
(y_CR_Ch_Lower(kk,ii))^(1/CR_Ch_Lower_time(ii));
        V_CR_Ch_Lower_time(kk)=mean(y_CR_Ch_Lower_time(kk,:));
    end
end
hist(V_CR_Ch_Lower_time,20);h =
findobj(gca,'Type','patch');set(h,'FaceColor',[.8 .8 .8]);
title('\bfSnake River Chinook Unimpounded scaled by time (per
day)','fontsize',9);
disp('The mean value for SNAKE RIVER CHINOOK UNIMPOUNDED survival
estimates scaled by time (per day) is ');disp(mean(V_CR_Ch_Lower_time))
S=sort(V_CR_Ch_Lower_time);
disp('Lower 95% CI is ');disp(S(250))
disp('Upper 95% CI is ');disp(S(9750))
axis([0 1 0 1.05*max(hist(V_CR_Ch_Lower_time,20))]);
The mean value for SNAKE RIVER CHINOOK UNIMPOUNDED survival estimates
scaled by time (per day) is
    0.8761

Lower 95% CI is
    0.8619

Upper 95% CI is
    0.8894
```

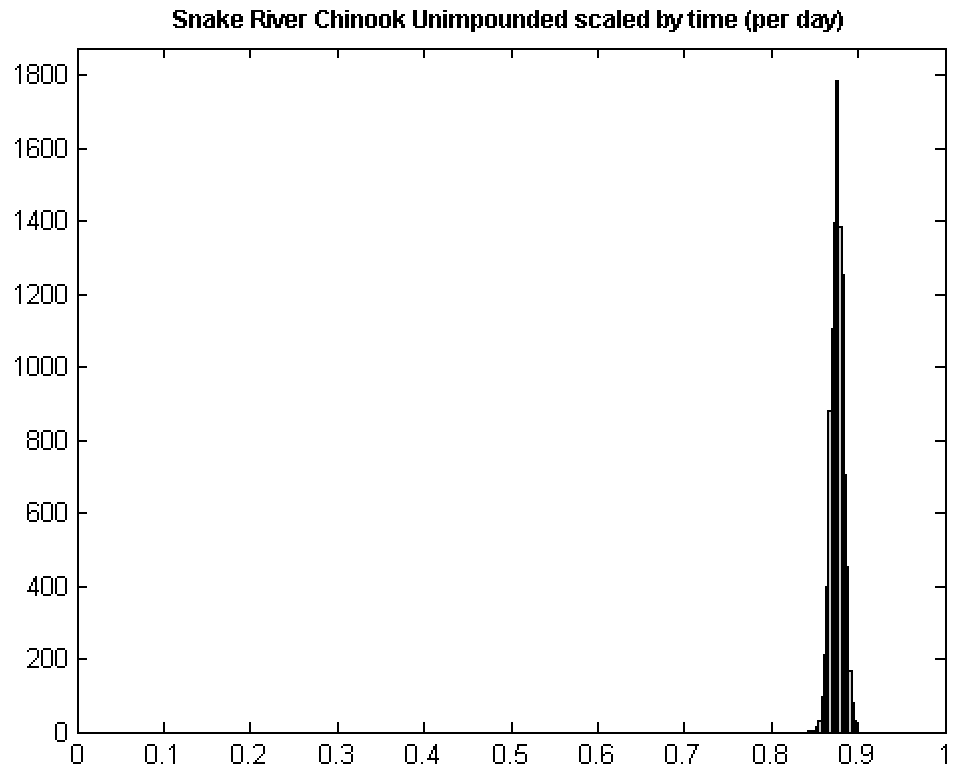

#8SNAKE RIVER CHINOOK UNIMPOUNDED comparison with FRASER CHINOOK scaled by time (per day)

```
%differences between pairs (CR avg. surv - FR avg. surv)
V_diff_8 = V_CR_Ch_Lower_time-V_FR_Ch_time;
hist(V_diff_8,50);h = findobj(gca,'Type','patch');set(h,'FaceColor',[.8
.8 .8]);
title('\bfDifferences between Snake R Chinook Unimpounded and FR scaled
by time (per day)','fontsize',9);
S=sort(V_diff_8);
disp('Lower 95% CI is ');disp(S(250))
disp('Upper 95% CI is ');disp(S(9750))
axis([-0.1  max(S)+0.1  0  1.05*max(hist(V_diff_8,50))]);
Lower 95% CI is
    0.0447

Upper 95% CI is
    0.4471
```

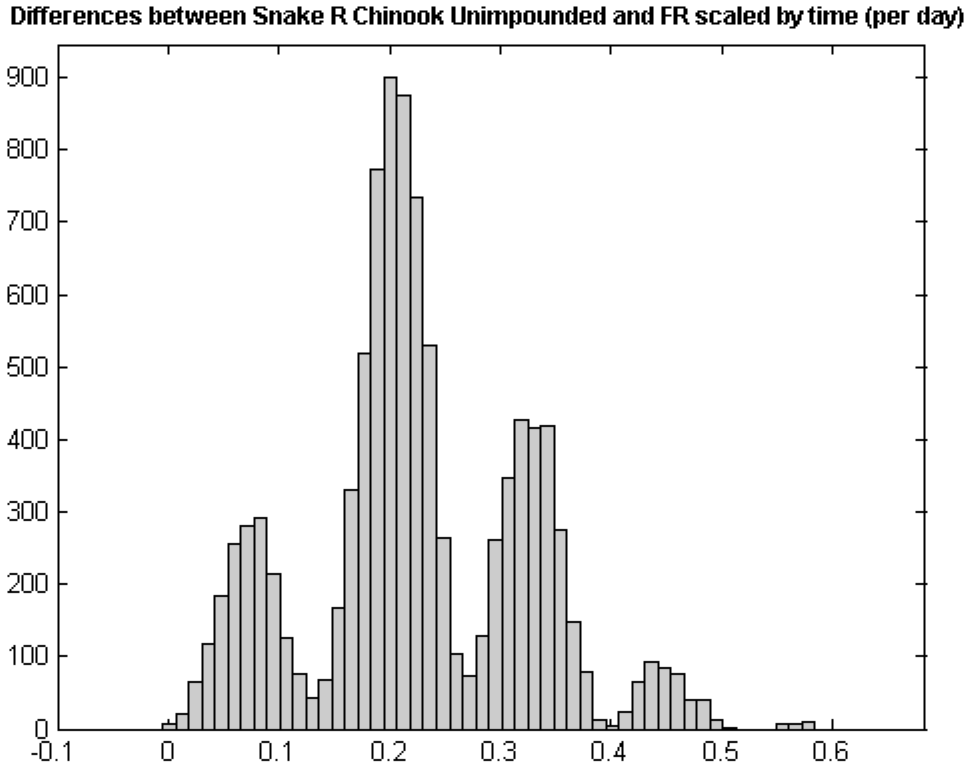

COLUMBIA-SNAKE ENTIRE CHINOOK scaled by time (per day) 2006

```
%median travel time
CR_Ch_Entire_time = [23.24];%Kintama results
```

```
V_CR_Ch_Entire_time_2006 = zeros(1,10000);
y_CR_Ch_Entire_time = zeros(10000,1);
for kk=1:10000
    for ii=1:length(CR_Ch_Entire_surv)
        y_CR_Ch_Entire_time(kk,ii) =
(y_CR_Ch_Entire_2006(kk,ii))^(1/CR_Ch_Entire_time);
        V_CR_Ch_Entire_time_2006(kk)=mean(y_CR_Ch_Entire_time(kk,:));
    end
end
hist(V_CR_Ch_Entire_time_2006,20);h =
findobj(gca,'Type','patch');set(h,'FaceColor',[.8 .8 .8]);
title('\bfEntire Columbia-Snake River Chinook scaled by time (per day)
- 2006 results','fontsize',9);
disp('The mean value for COLUMBIA-SNAKE ENTIRE CHINOOK survival
estimates scaled by time (per day) 2006 is ')
disp(mean(V_CR_Ch_Entire_time_2006))
S=sort(V_CR_Ch_Entire_time_2006);
disp('Lower 95% CI is ');disp(S(250))
disp('Upper 95% CI is ');disp(S(9750))
axis([0 1 0 1.05*max(hist(V_CR_Ch_Entire_time_2006,20))]);
The mean value for COLUMBIA-SNAKE ENTIRE CHINOOK survival estimates
scaled by time (per day) 2006 is
    0.9446

Lower 95% CI is
    0.9197

Upper 95% CI is
    0.9618
```

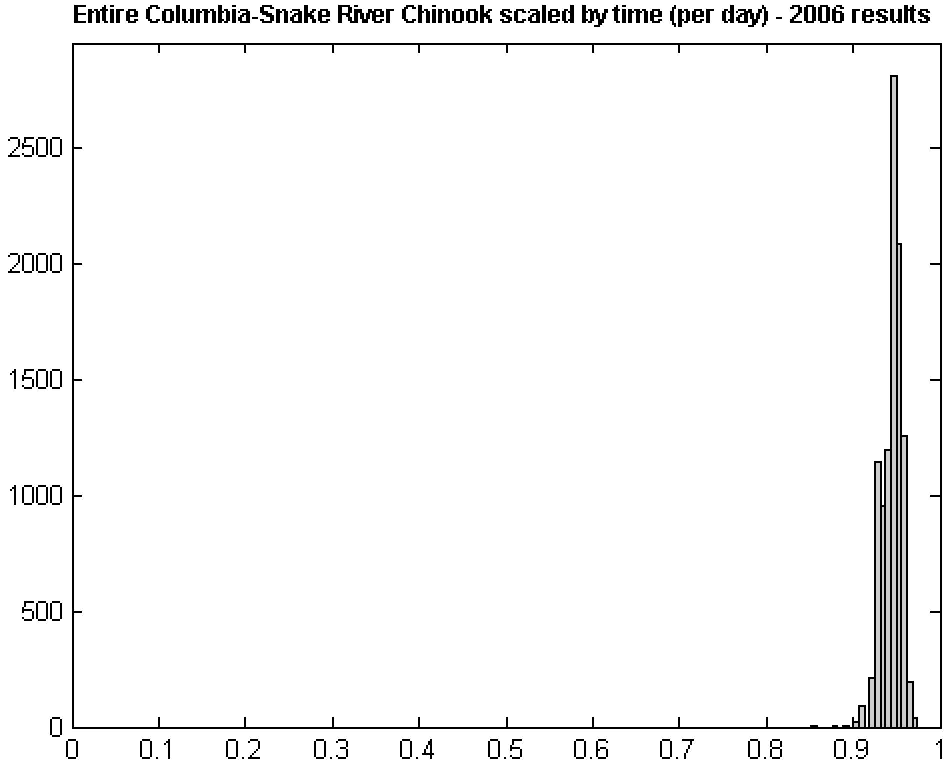

COLUMBIA-SNAKE ENTIRE CHINOOK scaled by time (per day) 2004

```
%the 2004 synthetic results
V_CR_Ch_Entire_time_2004=(y_CR_Ch_Lower.*y_CR_Ch_Upper(:,6)).^(1/(21.36
+3.42));
hist(V_CR_Ch_Entire_time_2004,20);h =
findobj(gca,'Type','patch');set(h,'FaceColor',[.8 .8 .8]);
title('\bfEntire Columbia-Snake River Chinook scaled by time (per day)
- 2004 results','fontsize',9);
disp('The mean value for COLUMBIA-SNAKE ENTIRE CHINOOK survival
estimates scaled by time (per day) 2004 is ')
disp(mean(V_CR_Ch_Entire_time_2004))
S=sort(V_CR_Ch_Entire_time_2004);
disp('Lower 95% CI is ');disp(S(250))
disp('Upper 95% CI is ');disp(S(9750))
axis([0 1 0 1.05*max(hist(V_CR_Ch_Entire_time_2004,20))]);
The mean value for COLUMBIA-SNAKE ENTIRE CHINOOK survival estimates
scaled by time (per day) 2004 is
    0.9411
```

```
Lower 95% CI is
    0.9306

Upper 95% CI is
    0.9502
```

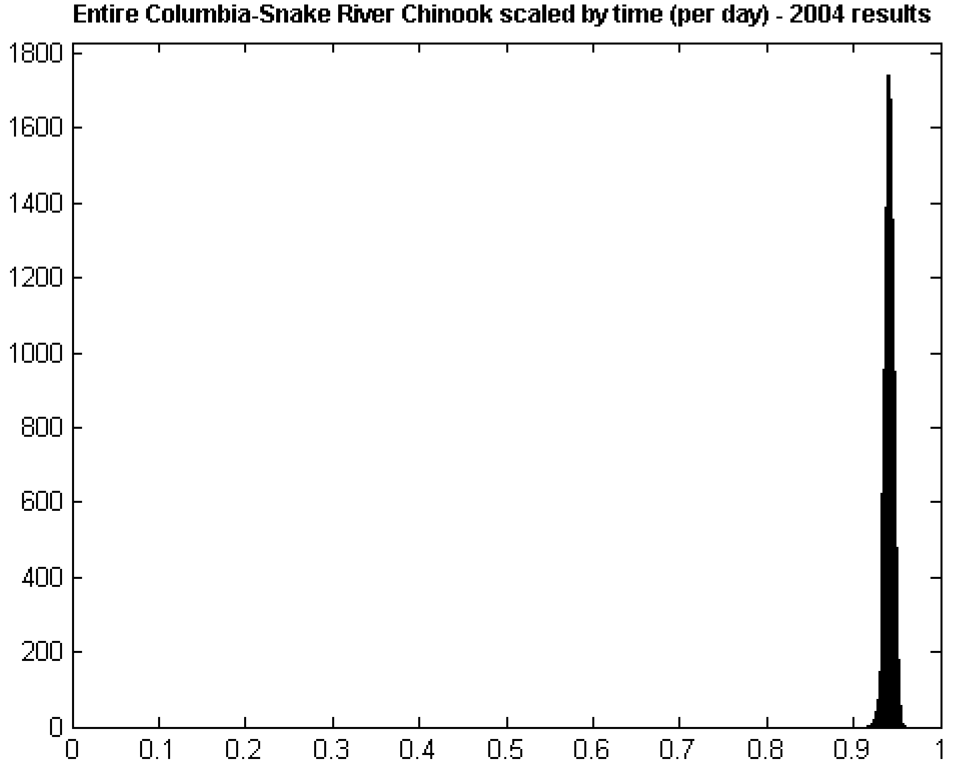

COLUMBIA-SNAKE ENTIRE CHINOOK scaled by time (per day) (2006 and 2004 combined)

```
V_CR_Ch_Entire_time = zeros(1,10000);
for kk=1:10000
```

```
V_CR_Ch_Entire_time(kk)=mean([V_CR_Ch_Entire_time_2006(kk),V_CR_Ch_Enti
re_time_2004(kk)]);
end
hist(V_CR_Ch_Entire_time,20);h =
findobj(gca,'Type','patch');set(h,'FaceColor',[.8 .8 .8]);
title('\bfEntire Columbia-Snake River Chinook scaled by time (per
day)','fontsize',9);
disp('The median value for COLUMBIA-SNAKE ENTIRE CHINOOK survival
estimates scaled by time (per day) is
');disp(median(V_CR_Ch_Entire_time))
S=sort(V_CR_Ch_Entire_time);
disp('Lower 95% CI is ');disp(S(250))
disp('Upper 95% CI is ');disp(S(9750))
axis([0 1 0 1.05*max(hist(V_CR_Ch_Entire_time,20))]);
The median value for COLUMBIA-SNAKE ENTIRE CHINOOK survival estimates
scaled by time (per day) is
    0.9433

Lower 95% CI is
    0.9300

Upper 95% CI is
    0.9532
```

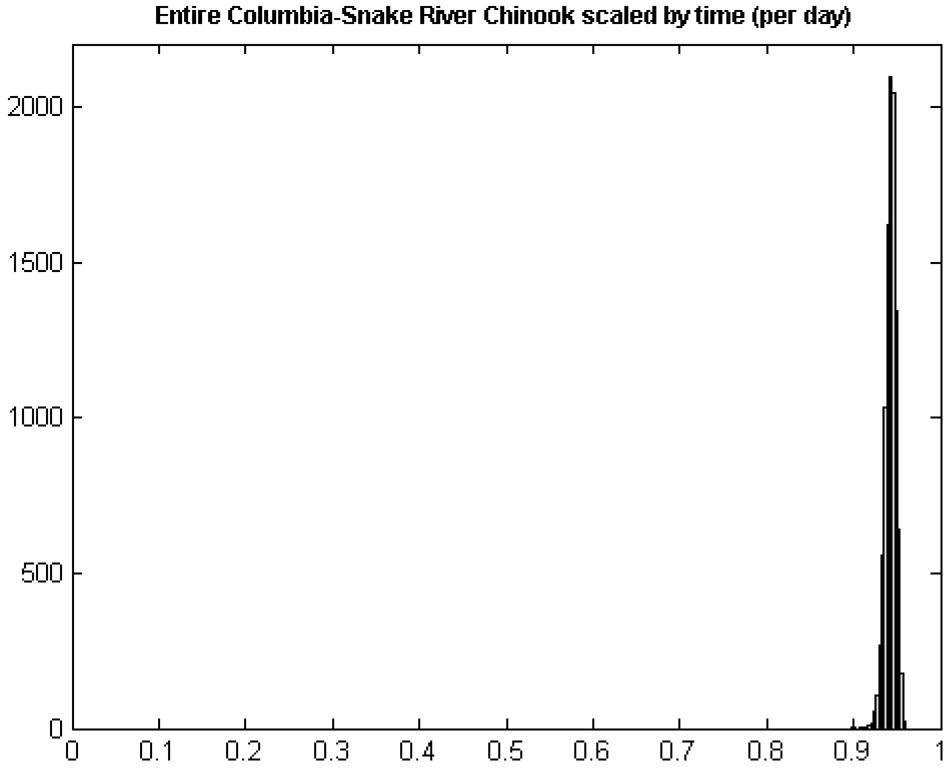

#9COLUMBIA-SNAKE ENTIRE CHINOOK comparison with FRASER CHINOOK scaled by time (per day)

```
%differences between pairs (CR avg. surv - FR avg. surv)
V_diff_9 = V_CR_Ch_Entire_time-V_FR_Ch_time;
hist(V_diff_9,50);h = findobj(gca,'Type','patch');set(h,'FaceColor',[.8
.8 .8]);
t=title('\bfDifferences between Entire Columbia-Snake R and FR scaled
by time (per day)','fontsize',9);
S=sort(V_diff_9);
disp('Lower 95% CI is ');disp(S(250))
disp('Upper 95% CI is ');disp(S(9750))
axis([-0.1  max(S)+0.1  0  1.05*max(hist(V_diff_9,50))]);
Lower 95% CI is
    0.1124

Upper 95% CI is
    0.5145
```

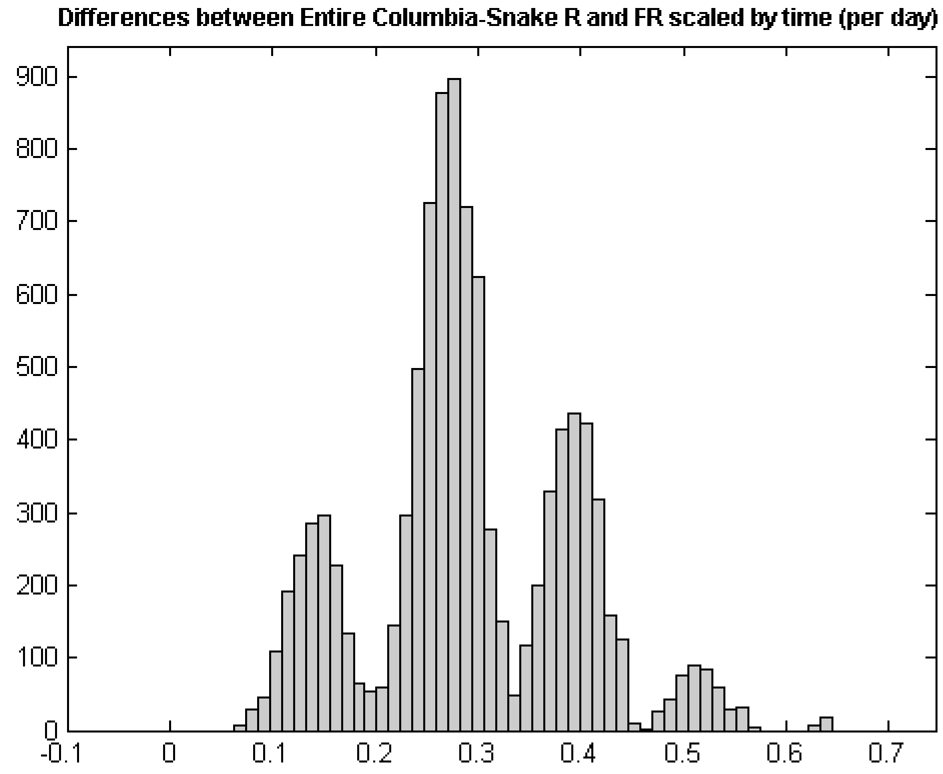

Published with MATLAB® 7.4

Steelhead

```
%This script will test the hypothesis that the estimated survivals for
%Fraser River and Columbia-Snake River are equal
%We will use the randraw.m script ('EFFICIENT RANDOM VARIATES
GENERATOR'
%from a binomial distribution)
%For the Fraser River Steelhead - there are 7 survival estimates
available
%For the Columbia River Steelhead - there are 8 PIT tag survival
estimates
%available for the Upper Columbia River, and 2 acoustic tag survival
%estimates for the Lower Columbia River (below Bonneville Dam)
%We assume a binomial distribution for the survival estimates,
%defined as B(S,N*), where S is the estimated survival proportion,
%and N* is the number of fish released (decreased from N to give the
%variance on the survival estimate reported)

%MATLAB Version 7.4.0.287 (R2007a)
```

FRASER RIVER STEELHEAD

```
clear all
%survival estimates calculated using th CJS method using Program MARK
FR_St_surv = [ .419, .251, .699, .223, .19, .201, .115];%Table 1
FR_St_SE = [ .157, .105, .169, .115, .082, .108, .078];%Table 1
%N_star is a calculated number of fish released that results in the
variance
%calculated by the CJS method
FR_St_N_star = round((FR_St_surv.*(1-FR_St_surv))./FR_St_SE.^2);
V_FR_St = zeros(1,10000);
y_FR_St = zeros(10000,7);
for kk=1:10000
    for ii=1:length(FR_St_surv)
        %for each individual group (ii), generate a random survival
estimate
        %from a binomial distribution B(FR_St_surv(ii),FR_St_star(ii))
        y_FR_St(kk,ii) = randraw('binom', [FR_St_N_star(ii),
FR_St_surv(ii)], 1)/FR_St_N_star(ii);
        %for a set (kk) of seven random generated survival estimates,
calculate
        %the average across the seven stocks
        V_FR_St(kk)=mean(y_FR_St(kk,:));
```

```
    end
end
hist(V_FR_St,20);h = findobj(gca,'Type','patch');set(h,'FaceColor',[.8
.8 .8]);
title('\bfFraser River Steelhead','fontsize',9);
disp('The mean value for FRASER RIVER STEELHEAD survival estimates is
');disp(mean(V_FR_St))
S=sort(V_FR_St);
disp('Lower 95% CI is ');disp(S(250))
disp('Upper 95% CI is ');disp(S(9750))
axis([0 1 0 1.05*max(hist(V_FR_St,20))]);
The mean value for FRASER RIVER STEELHEAD survival estimates is
    0.3000
Lower 95% CI is
    0.2118

Upper 95% CI is
    0.3904
```

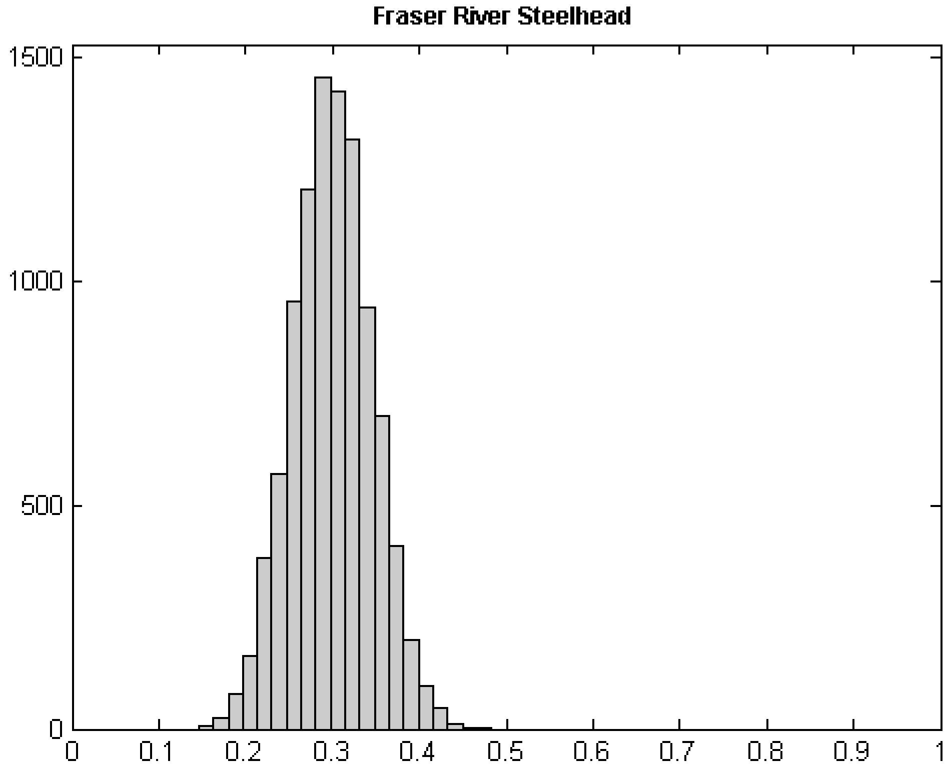

SNAKE RIVER STEELHEAD IMPOUNDED

```
%PIT tags survival estimates (NOAA)
CR_St_Upper_surv = [.457, .462, .4, .379, .038, .234, .288, .418];
%standard errors for the PIT tags survival estimates (NOAA)
```

```
CR_Upper_SE = [.067, .050, .016, .032, .003, .045, .011, .052];
%N_star is calculated to reflect the reported standard errors
CR_St_Upper_N_star = round((CR_St_Upper_surv.*(1-
CR_St_Upper_surv))./CR_Upper_SE.^2);
V_CR_St_Upper = zeros(1,10000);
y_CR_St_Upper = zeros(10000,8);
for kk=1:10000
    for ii=1:length(CR_St_Upper_surv)
        y_CR_St_Upper(kk,ii) = randraw('binom',
[CR_St_Upper_N_star(ii), CR_St_Upper_surv(ii)],
1)/CR_St_Upper_N_star(ii);
        V_CR_St_Upper(kk)=mean(y_CR_St_Upper(kk,:));
    end
end
hist(V_CR_St_Upper,20);h =
findobj(gca,'Type','patch');set(h,'FaceColor',[.8 .8 .8]);
title('\bfUpper Columbia River Steelhead','fontsize',9);
disp('The mean value for SNAKE RIVER STEELHEAD IMPOUNDED survival
estimates is ');disp(mean(V_CR_St_Upper))
S=sort(V_CR_St_Upper);
disp('Lower 95% CI is ');disp(S(250))
disp('Upper 95% CI is ');disp(S(9750))
axis([0 1 0 1.05*max(hist(V_CR_St_Upper,20))]);
The mean value for SNAKE RIVER STEELHEAD IMPOUNDED survival estimates
is
    0.3345

Lower 95% CI is
    0.3067

Upper 95% CI is
    0.3630
```

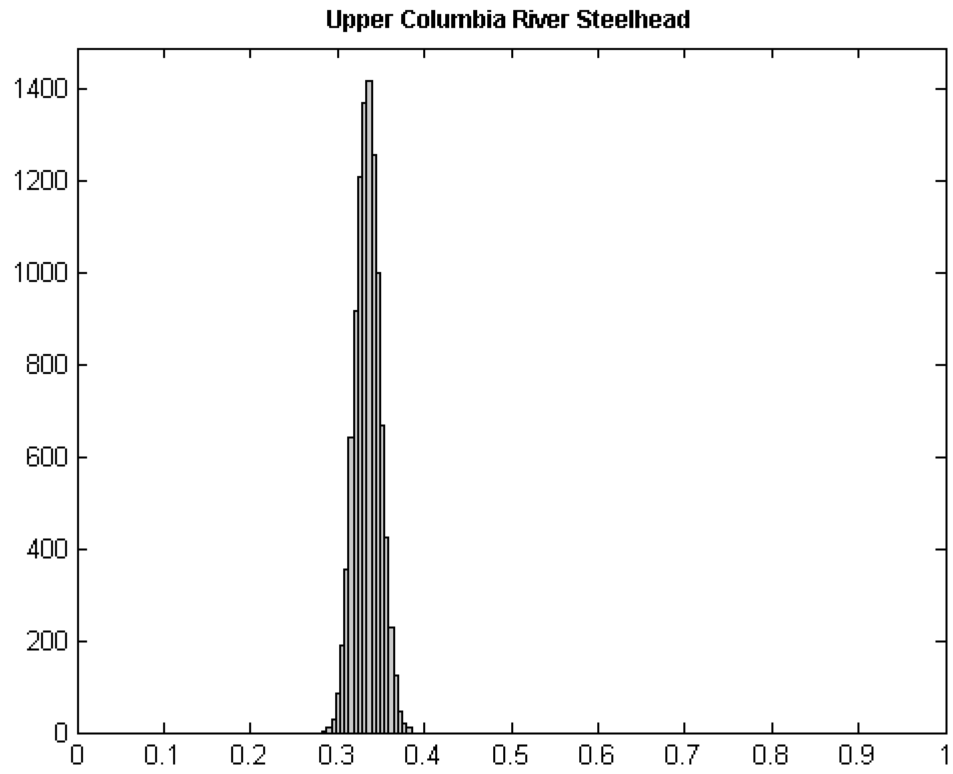

#1SNAKE RIVER STEELHEAD IMPOUNDED comparison with FRASER RIVER STEELHEAD

```
%differences between pairs (CR avg. surv - FR avg. surv)
V_diff_1 = V_CR_St_Upper-V_FR_St;
hist(V_diff_1,50);h = findobj(gca,'Type','patch');set(h,'FaceColor',[.8
.8 .8]);
title('\bfDifferences between Upper CR and FR','fontsize',9);
S=sort(V_diff_1);
disp('Lower 95% CI is ');disp(S(250))
disp('Upper 95% CI is ');disp(S(9750))
axis([min(S)-0.1  max(S)+0.1  0  1.05*max(hist(V_diff_1,50))]);
Lower 95% CI is
   -0.0593

Upper 95% CI is
    0.1284
```

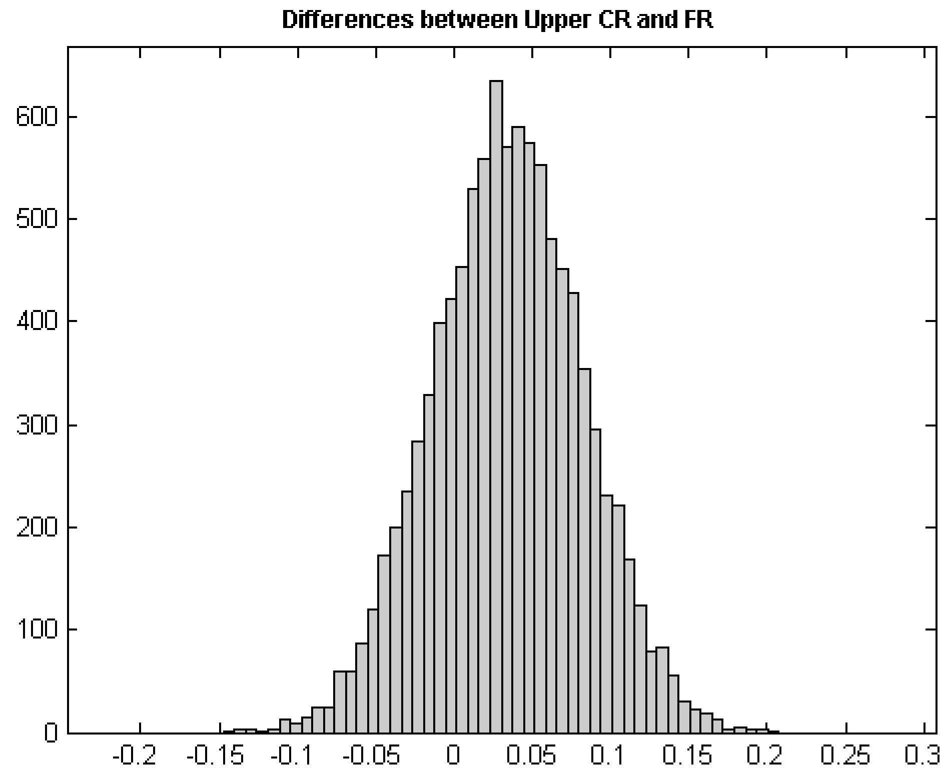

SNAKE RIVER STEELHEAD UNIMPOUNDED

```
%Ben Clemmens (2002,2003)
CR_St_Lower_surv = [.76, .63];
```

```
CR_Lower_SE = [.047, .019];
%N_star is calculated to reflect the reported standard errors
CR_St_Lower_N_star = round((CR_St_Lower_surv.*(1-
CR_St_Lower_surv))./CR_Lower_SE.^2);
V_CR_St_Lower = zeros(1,10000);
y_CR_St_Lower = zeros(10000,2);
for kk=1:10000
    for ii=1:length(CR_St_Lower_surv)
        y_CR_St_Lower(kk,ii) = randraw('binom',
[CR_St_Lower_N_star(ii), CR_St_Lower_surv(ii)],
1)/CR_St_Lower_N_star(ii);
        V_CR_St_Lower(kk)=mean(y_CR_St_Lower(kk,:));
    end
end
hist(V_CR_St_Lower,20);h =
findobj(gca,'Type','patch');set(h,'FaceColor',[.8 .8 .8]);
title('\bfLower Columbia River Steelhead','fontsize',9);
disp('The mean value for SNAKE RIVER STEELHEAD UNIMPOUNDED survival
estimates is ');disp(mean(V_CR_St_Lower))
S=sort(V_CR_St_Lower);
disp('Lower 95% CI is ');disp(S(250))
disp('Lower 95% CI is ');disp(S(9750))
axis([0 1 0 1.05*max(hist(V_CR_St_Lower,20))]);
The mean value for SNAKE RIVER STEELHEAD UNIMPOUNDED survival estimates
is
    0.6948

Lower 95% CI is
    0.6450

Lower 95% CI is
    0.7437
```

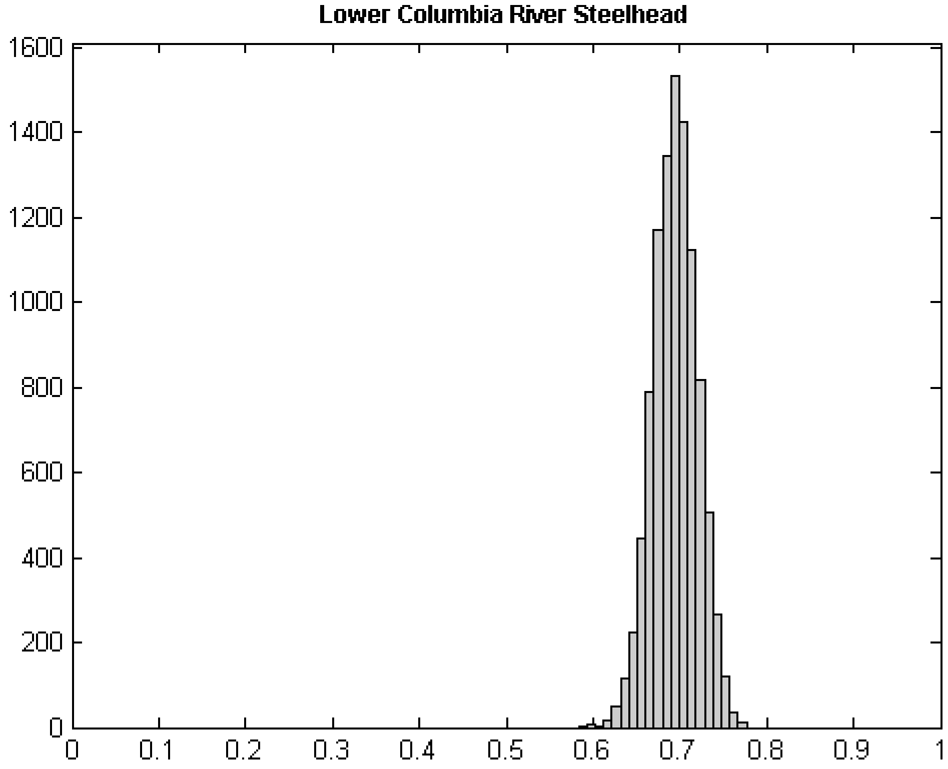

#2SNAKE RIVER STEELHEAD UNIMPOUNDED comparison with FRASER RIVER STEELHEAD

```
%differences between pairs (CR avg. surv - FR avg. surv)
V_diff_2 = V_CR_St_Lower-V_FR_St;
hist(V_diff_2,50);h = findobj(gca,'Type','patch');set(h,'FaceColor',[.8
.8 .8]);
title('\bfDifferences between Lower CR and FR','fontsize',9);
S=sort(V_diff_2);
disp('Lower 95% CI is ');disp(S(250))
disp('Upper 95% CI is ');disp(S(9750))
if min(S)>0; axis([-0.1  max(S)+0.1  0
1.05*max(hist(V_diff_2,50))]);end
if max(S)<0; axis([min(S)-0.1  0.1  0
1.05*max(hist(V_diff_2,50))]);end
Lower 95% CI is
    0.2920

Upper 95% CI is
    0.4945
```

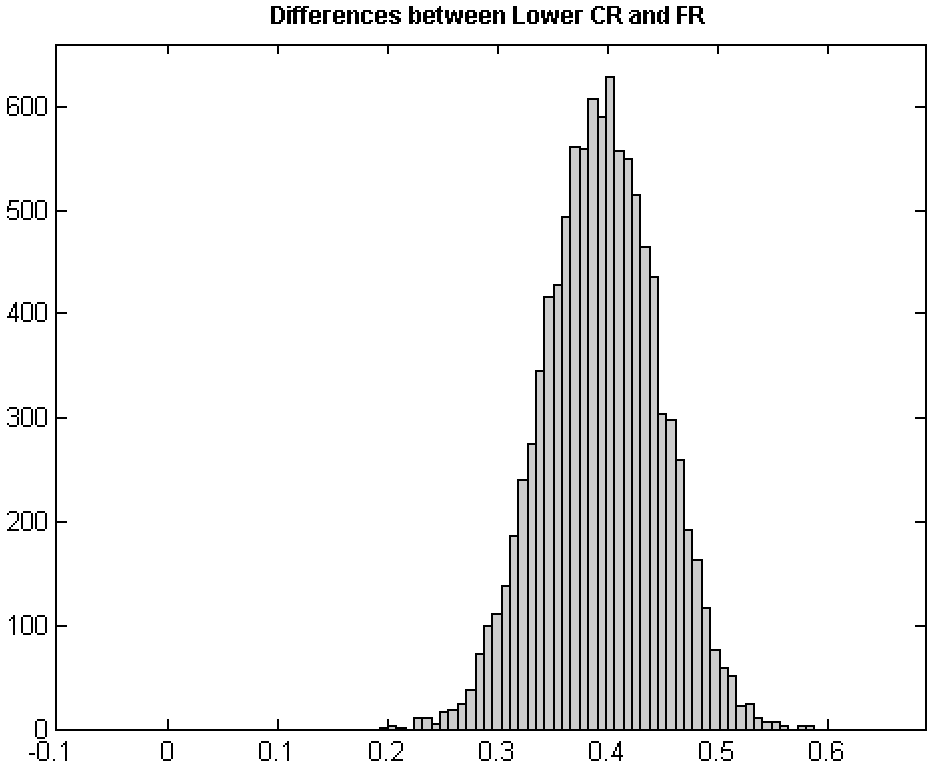

COLUMBIA-SNAKE RIVER ENTIRE STEELHEAD 2002

```
%the 2002 synthetic results
V_CR_St_Entire_2002=y_CR_St_Lower(:,1).*y_CR_St_Upper(:,6);
hist(V_CR_St_Entire_2002,20);h =
findobj(gca,'Type','patch');set(h,'FaceColor',[.8 .8 .8]);
```

```
title('\bfEntire Columbia-Snake River Steelhead - 2002
results','fontsize',9);
disp('The mean value for COLUMBIA-SNAKE RIVER ENTIRE STEELHEAD 2002
survival estimates is ');disp(mean(V_CR_St_Entire_2002))
S=sort(V_CR_St_Entire_2002);
disp('Lower 95% CI is ');disp(S(250))
disp('Upper 95% CI is ');disp(S(9750))
axis([0 1 0 1.05*max(hist(V_CR_St_Entire_2002,20))]);
The mean value for COLUMBIA-SNAKE RIVER ENTIRE STEELHEAD 2002 survival
estimates is
    0.1780

Lower 95% CI is
    0.1118

Upper 95% CI is
    0.2502
```

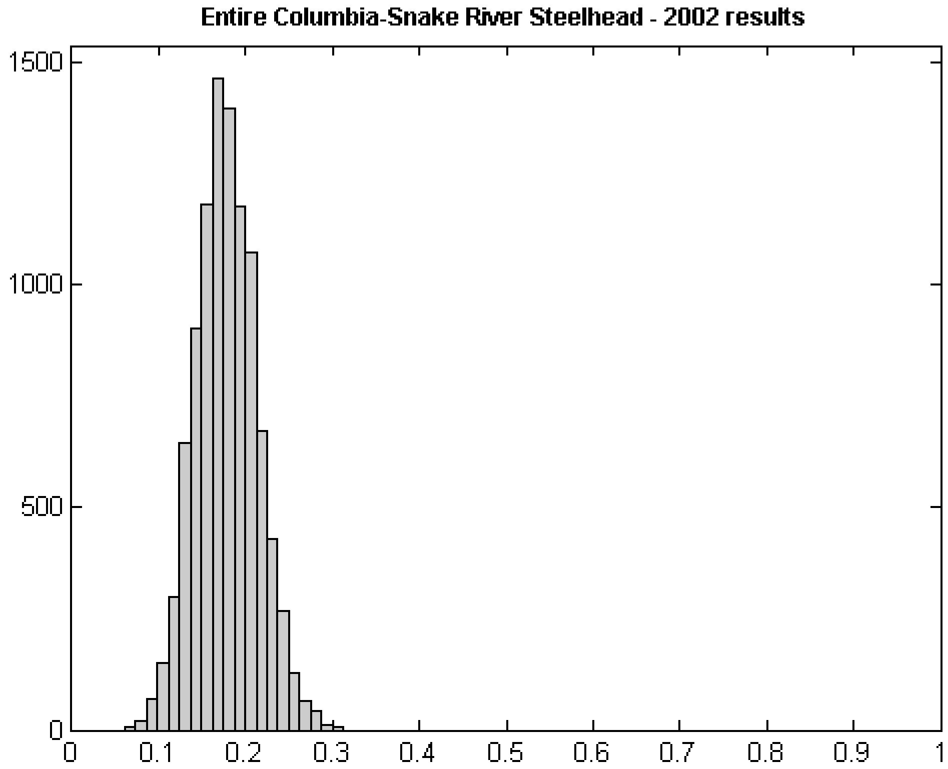

COLUMBIA-SNAKE RIVER-ENTIRE STEELHEAD 2003

```
%the 2003 synthetic results
V_CR_St_Entire_2003=y_CR_St_Lower(:,2).*y_CR_St_Upper(:,7);
hist(V_CR_St_Entire_2003,20);h =
findobj(gca,'Type','patch');set(h,'FaceColor',[.8 .8 .8]);
title('\bfEntire Columbia-Snake River Steelhead - 2003
results','fontsize',9);
disp('The mean value for COLUMBIA-SNAKE RIVER ENTIRE STEELHEAD 2003
survival estimates is ');disp(mean(V_CR_St_Entire_2003))
S=sort(V_CR_St_Entire_2003);
disp('Lower 95% CI is ');disp(S(250))
disp('Upper 95% CI is ');disp(S(9750))
axis([0 1 0 1.05*max(hist(V_CR_St_Entire_2003,20))]);
The mean value for COLUMBIA-SNAKE RIVER ENTIRE STEELHEAD 2003 survival
estimates is
    0.1815

Lower 95% CI is
    0.1646

Upper 95% CI is
    0.1990
```

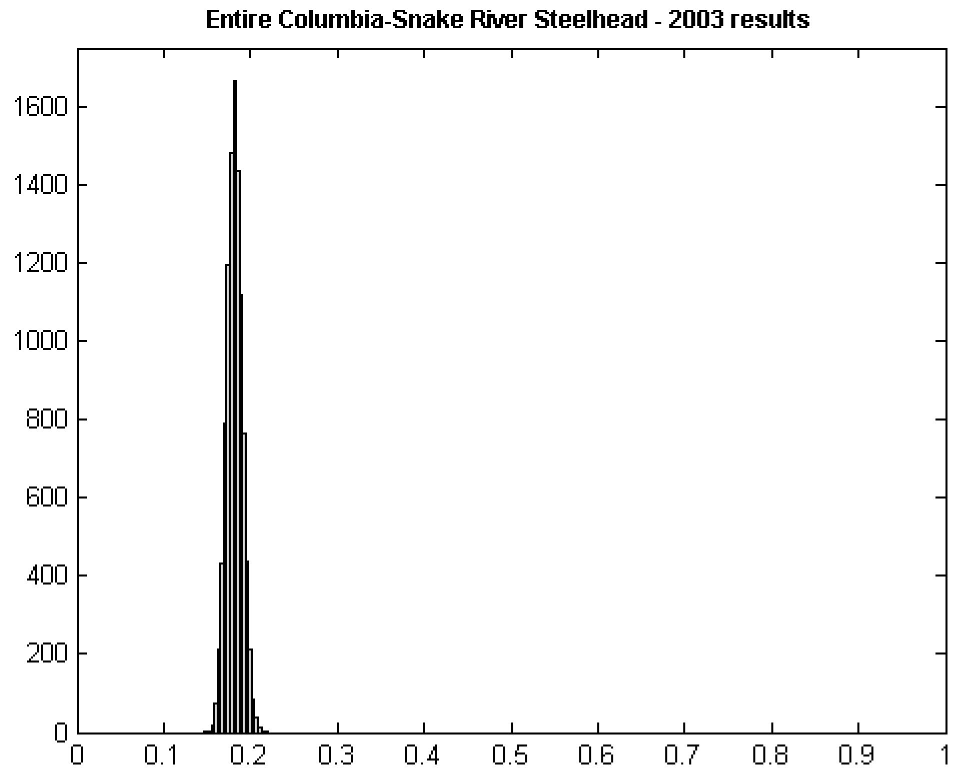

#3COLUMBIA-SNAKE RIVER ENTIRE STEELHEAD comparison with FRASER RIVER STEELHEAD

```
V_CR_St_Entire=zeros(1,10000);
for kk=1:10000

V_CR_St_Entire(kk)=mean([V_CR_St_Entire_2002(kk),V_CR_St_Entire_2003(kk
)]);
end
```

```
V_diff_3 = V_CR_St_Entire-V_FR_St; %between Entire CR Ch and FR
Steelhead
hist(V_diff_3,50);h = findobj(gca,'Type','patch');set(h,'FaceColor',[.8
.8 .8]);
title('\bfDifferences between Entire Columbia-Snake R and FR (2006
only)','fontsize',9);
S=sort(V_diff_3);
disp('Lower 95% CI is ');disp(S(250))
disp('Upper 95% CI is ');disp(S(9750))
if min(S)>0; axis([-0.1  max(S)+0.1  0
1.05*max(hist(V_diff_3,50))]);end
if max(S)<0; axis([min(S)-0.1  0.1  0
1.05*max(hist(V_diff_3,50))]);end
Lower 95% CI is
   -0.2174

Upper 95% CI is
   -0.0224
```

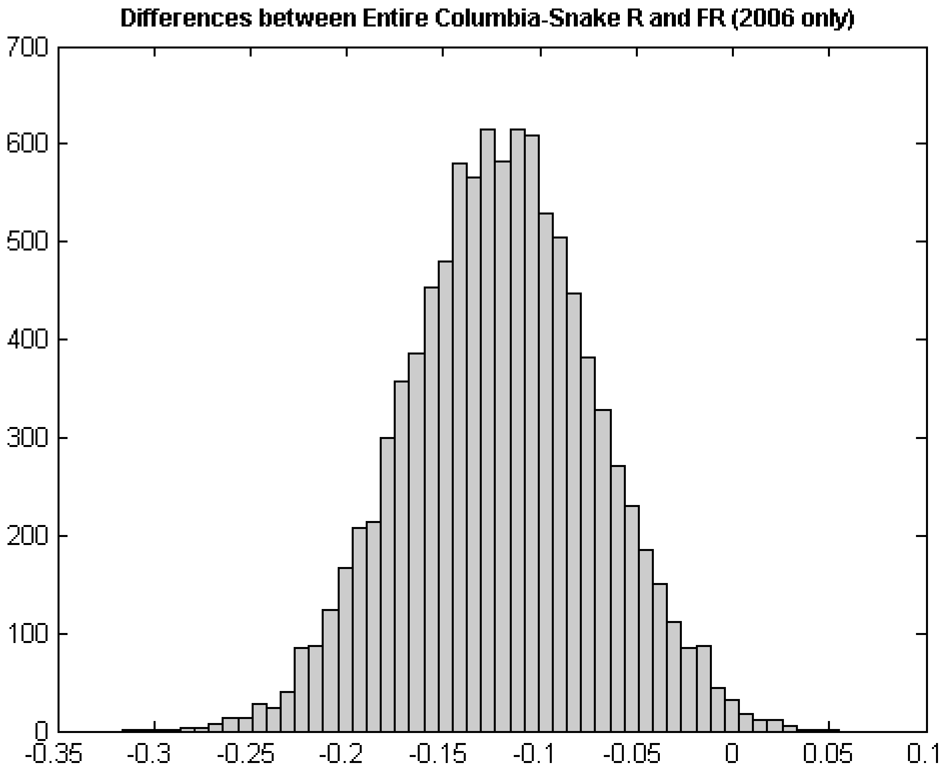

FRASER RIVER STEELHEAD scaled by distance (per 100 km)

```
FR_St_dist = [324.25,330.8,351.53,351.53,342.05,362.78,362.78];
V_FR_St_dist = zeros(1,10000);
y_FR_St_dist = zeros(10000,7);
for kk=1:10000
    for ii=1:length(FR_St_surv)
        y_FR_St_dist(kk,ii) = (y_FR_St(kk,ii))^(100/FR_St_dist(ii));
        V_FR_St_dist(kk)=mean(y_FR_St_dist(kk,:));
    end
end
hist(V_FR_St_dist,20);h =
findobj(gca,'Type','patch');set(h,'FaceColor',[.8 .8 .8]);
title('\bfFraser River Steelhead scaled by distance (per 100
km)','fontsize',9);
disp('The mean value for FRASER RIVER STEELHEAD survival estimates
scaled by distance (per 100 km) is ');disp(mean(V_FR_St_dist))
S=sort(V_FR_St_dist);
disp('Lower 95% CI is ');disp(S(250))
disp('Upper 95% CI is ');disp(S(9750))
axis([0 1 0 1.05*max(hist(V_FR_St_dist,20))]);
The mean value for FRASER RIVER STEELHEAD survival estimates scaled by
distance (per 100 km) is
    0.6573

Lower 95% CI is
    0.5424

Upper 95% CI is
    0.7351
```

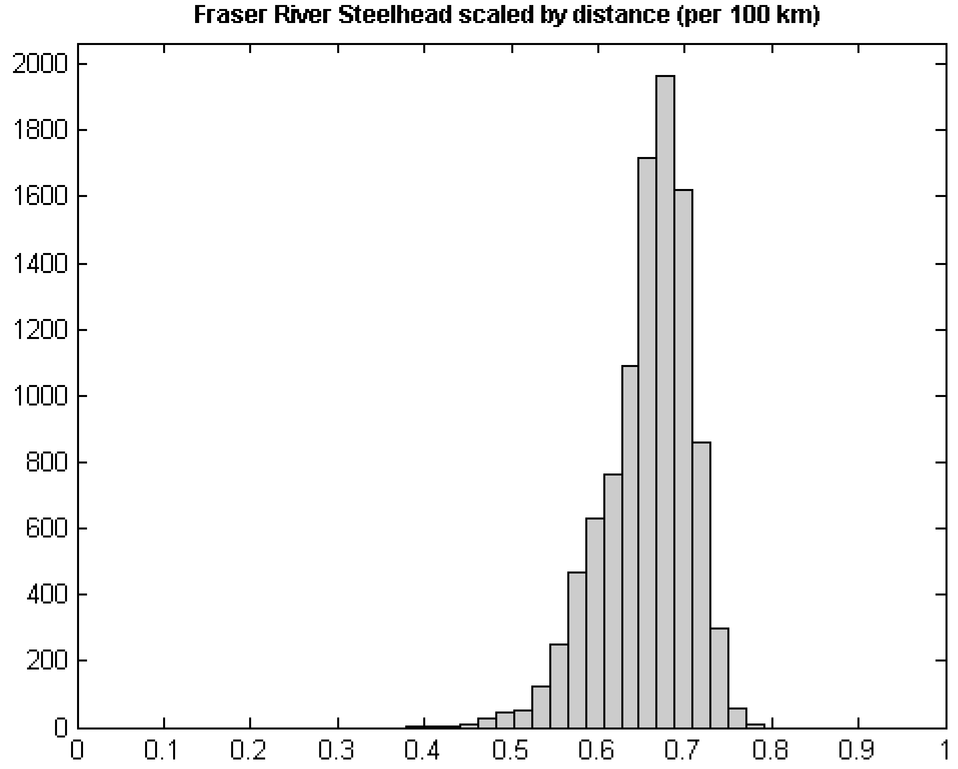

SNAKE RIVER STEELHEAD IMPOUNDED scaled by distance (per 100 km)

```
CR_St_Upper_dist = [506];%PIT results
V_CR_St_Upper_dist = zeros(1,10000);
y_CR_St_Upper_dist = zeros(10000,8);
for kk=1:10000
    for ii=1:length(CR_St_Upper_surv)
        y_CR_St_Upper_dist(kk,ii) =
(y_CR_St_Upper(kk,ii))^(100/CR_St_Upper_dist);
        V_CR_St_Upper_dist(kk)=mean(y_CR_St_Upper_dist(kk,:));
    end
end
hist(V_CR_St_Upper_dist,20); h =
findobj(gca,'Type','patch');set(h,'FaceColor',[.8 .8 .8]);
```

```
title('\bfSnake River Steelhead Impounded scaled by distance (per 100 
km)','fontsize',9);
disp('The mean value for SNAKE RIVER STEELHEAD IMPOUNDED survival 
estimates scaled by distance (per 100 km) is 
');disp(mean(V_CR_St_Upper_dist))
S=sort(V_CR_St_Upper_dist);
disp('Lower 95% CI is ');disp(S(250))
disp('Upper 95% CI is ');disp(S(9750))
axis([0 1 0 1.05*max(hist(V_CR_St_Upper_dist,20))]);
The mean value for SNAKE RIVER STEELHEAD IMPOUNDED survival estimates 
scaled by distance (per 100 km) is 
    0.7833

Lower 95% CI is 
    0.7705

Upper 95% CI is 
    0.7954
```

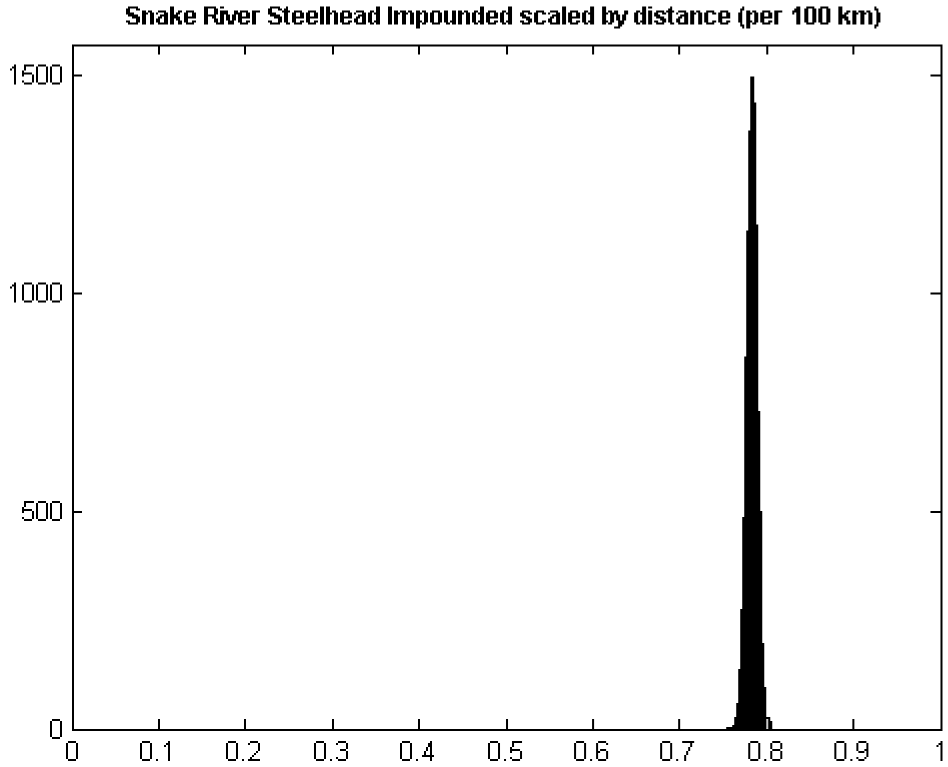

#4SNAKE RIVER STEELHEAD IMPOUNDED comparison with FRASER STEELHEAD scaled by distance (per 100 km)

```
%differences between pairs (CR avg. surv - FR avg. surv)
V_diff_4 = V_CR_St_Upper_dist-V_FR_St_dist;
hist(V_diff_4,50);h = findobj(gca,'Type','patch');set(h,'FaceColor',[.8
.8 .8]);
title('\bfDifferences between Snake R Steelhead Impounded and FR scaled
by distance (per 100 km)','fontsize',9);
S=sort(V_diff_4);
disp('Lower 95% CI is ');disp(S(250))
disp('Upper 95% CI is ');disp(S(9750))
axis([-0.1  max(S)+0.1  0  1.05*max(hist(V_diff_4,50))]);
Lower 95% CI is
    0.0474

Upper 95% CI is
    0.2418
```

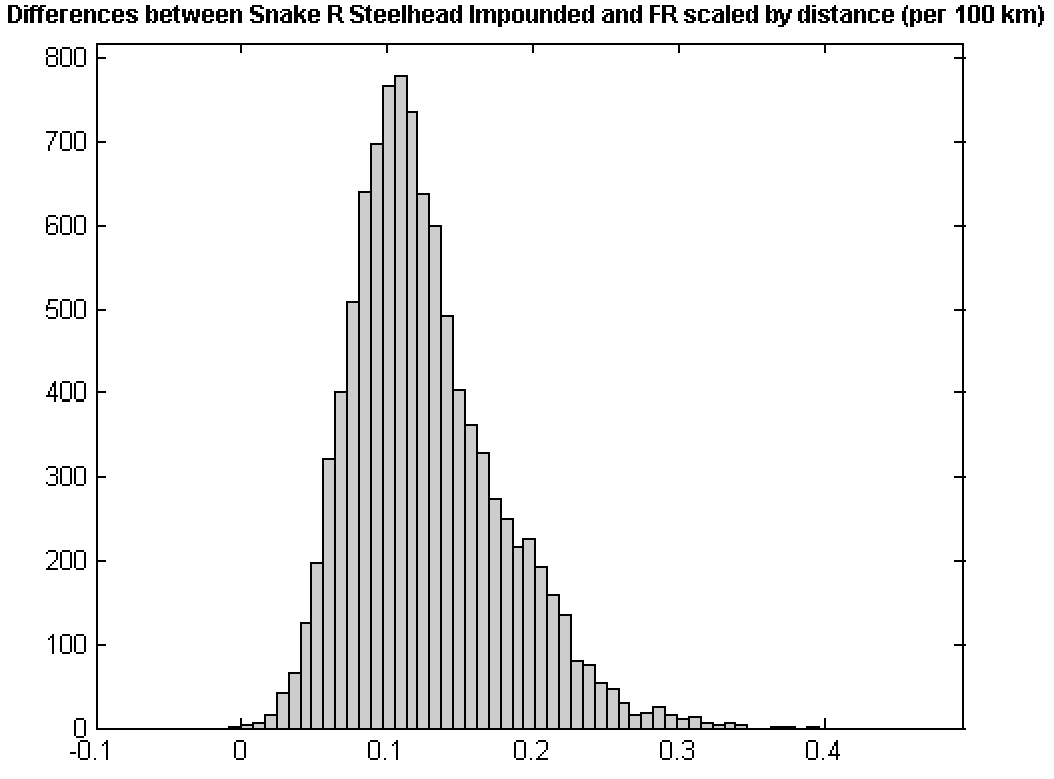

SNAKE RIVER STEELHEAD UNIMPOUNDED scaled by distance (per 100 km)

```
CR_St_Lower_dist = [212];
V_CR_St_Lower_dist = zeros(1,10000);
y_CR_St_Lower_dist = zeros(10000,2);
for kk=1:10000
    for ii=1:length(CR_St_Lower_surv)
        y_CR_St_Lower_dist(kk,ii) =
(y_CR_St_Lower(kk,ii))^(100/CR_St_Lower_dist);
        V_CR_St_Lower_dist(kk)=mean(y_CR_St_Lower_dist(kk,:));
    end
end
hist(V_CR_St_Lower_dist,20); h =
findobj(gca,'Type','patch');set(h,'FaceColor',[.8 .8 .8]);
title('\bfSnake River Steelhead Unimpounded scaled by distance (per 100
km)','fontsize',9);
disp('The mean value for SNAKE RIVER STEELHEAD UNIMPOUNDED survival
estimates scaled by distance (per 100 km) is
');disp(mean(V_CR_St_Lower_dist))
S=sort(V_CR_St_Lower_dist);
disp('Lower 95% CI is ');disp(S(250))
disp('Upper 95% CI is ');disp(S(9750))
axis([0 1 0 1.05*max(hist(V_CR_St_Lower_dist,20))]);
The mean value for SNAKE RIVER STEELHEAD UNIMPOUNDED survival estimates
scaled by distance (per 100 km) is
    0.8410

Lower 95% CI is
    0.8127

Upper 95% CI is
    0.8678
```

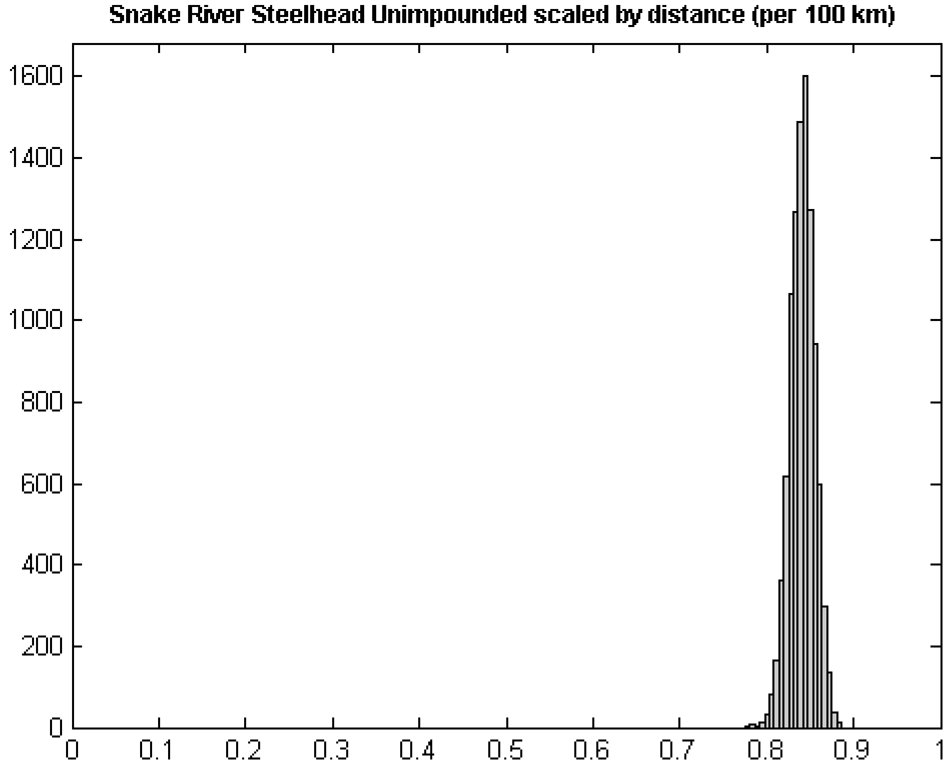

#5SNAKE RIVER STEELHEAD UNIMPOUNDED comparison with FRASER STEELHEAD scaled by distance (per 100 km)

```
%differences between pairs (CR avg. surv - FR avg. surv)
V_diff_5 = V_CR_St_Lower_dist-V_FR_St_dist;
hist(V_diff_5,50);h = findobj(gca,'Type','patch');set(h,'FaceColor',[.8
.8 .8]);
title('\bfDifferences between Snake R Steelhead Unimpounded and FR
scaled by distance (per 100 km)','fontsize',9);
S=sort(V_diff_5);
disp('Lower 95% CI is ');disp(S(250))
disp('Upper 95% CI is ');disp(S(9750))
Lo_V_diff_5 = S(250);Hi_V_diff_5 = S(9750);%axis([-0.3 0.3 0 2500]);
if min(S)>0; axis([-0.1  max(S)+0.1  0
1.05*max(hist(V_diff_5,50))]);end
```

```
if max(S)<0; axis([min(S)-0.1  0.1  0
1.05*max(hist(V_diff_5,50))]);end
Lower 95% CI is
    0.1010

Upper 95% CI is
    0.3007
```

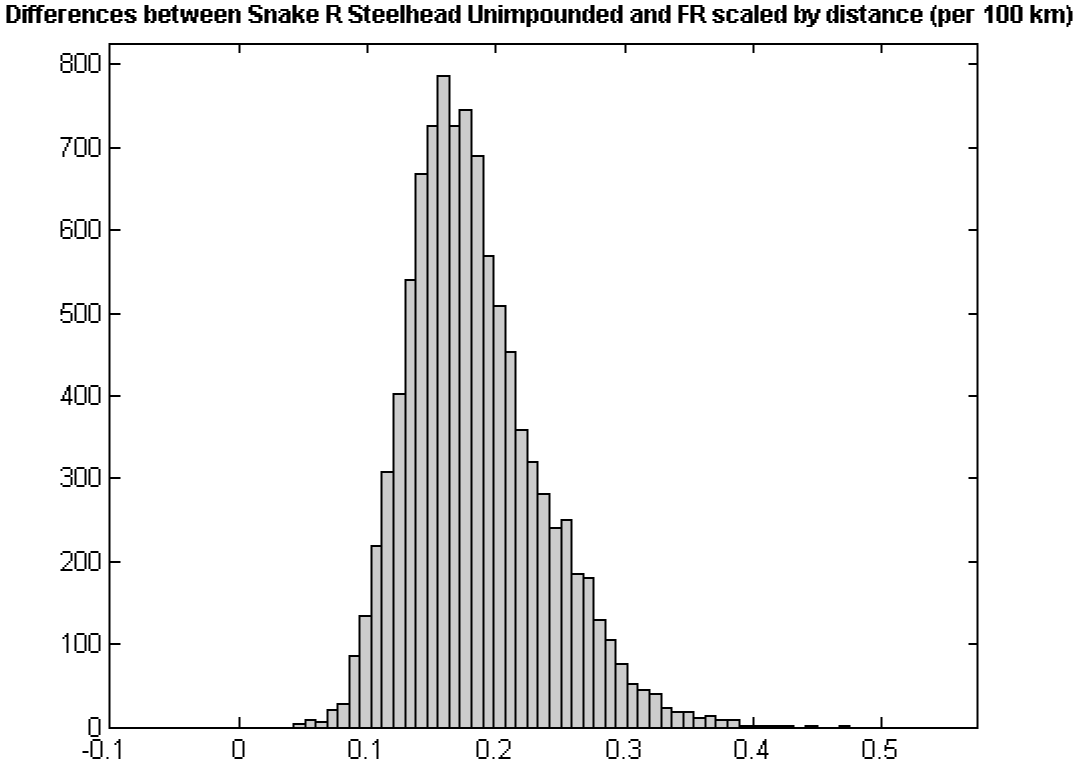

COLUMBIA-SNAKE ENTIRE STEELHEAD scaled by distance (per 100 km) 2002

```
%the 2002 synthetic results
V_CR_St_Entire_dist_2002=(y_CR_St_Lower(:,1).*y_CR_St_Upper(:,6)).^(100
/(506+212));
hist(V_CR_St_Entire_dist_2002,20);h =
findobj(gca,'Type','patch');set(h,'FaceColor',[.8 .8 .8]);
title('\bfEntire Columbia-Snake River Steelhead scaled by distance (per
100 km) - 2002 results','fontsize',9);
disp('The mean value for COLUMBIA-SNAKE ENTIRE STEELHEAD survival
estimates scaled by distance (per 100 km) 2002 is
');disp(mean(V_CR_St_Entire_dist_2002))
S=sort(V_CR_St_Entire_dist_2002);
disp('Lower 95% CI is ');disp(S(250))
disp('Upper 95% CI is ');disp(S(9750))
axis([0 1 0 1.05*max(hist(V_CR_St_Entire_dist_2002,20))]);
The mean value for COLUMBIA-SNAKE ENTIRE STEELHEAD survival estimates
scaled by distance (per 100 km) 2002 is
    0.7844

Lower 95% CI is
    0.7370

Upper 95% CI is
    0.8245
```

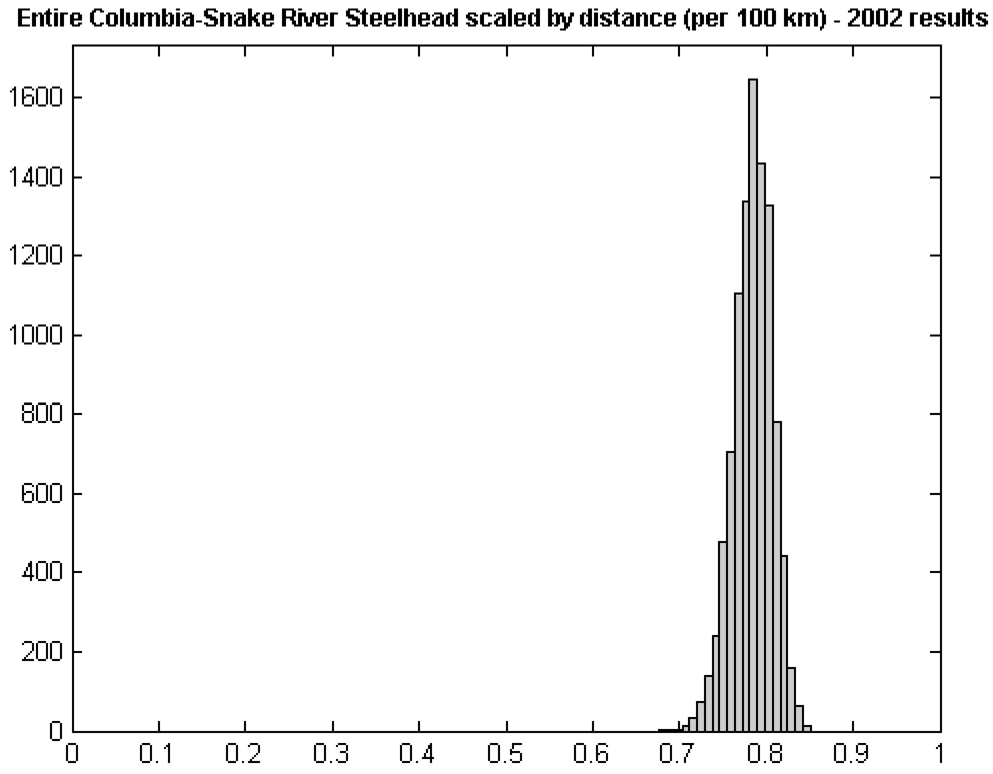

COLUMBIA-SNAKE ENTIRE STEELHEAD scaled by distance (per 100 km) 2003

```
%the 2003 synthetic results
V_CR_St_Entire_dist_2003=(y_CR_St_Lower(:,2).*y_CR_St_Upper(:,7)).^(100
/(506+212));
hist(V_CR_St_Entire_dist_2003,20);h =
findobj(gca,'Type','patch');set(h,'FaceColor',[.8 .8 .8]);
title('\bfEntire Columbia-Snake River Steelhead scaled by distance (per
100 km) - 2003 results','fontsize',9);
disp('The mean value for COLUMBIA-SNAKE ENTIRE STEELHEAD survival
estimates scaled by distance (per 100 km) 2003 is
');disp(mean(V_CR_St_Entire_dist_2003))
S=sort(V_CR_St_Entire_dist_2003);
disp('Lower 95% CI is ');disp(S(250))
disp('Upper 95% CI is ');disp(S(9750))
axis([0 1 0 1.05*max(hist(V_CR_St_Entire_dist_2003,20))]);
The mean value for COLUMBIA-SNAKE ENTIRE STEELHEAD survival estimates
scaled by distance (per 100 km) 2003 is
    0.7884

Lower 95% CI is
    0.7778

Upper 95% CI is
    0.7986
```

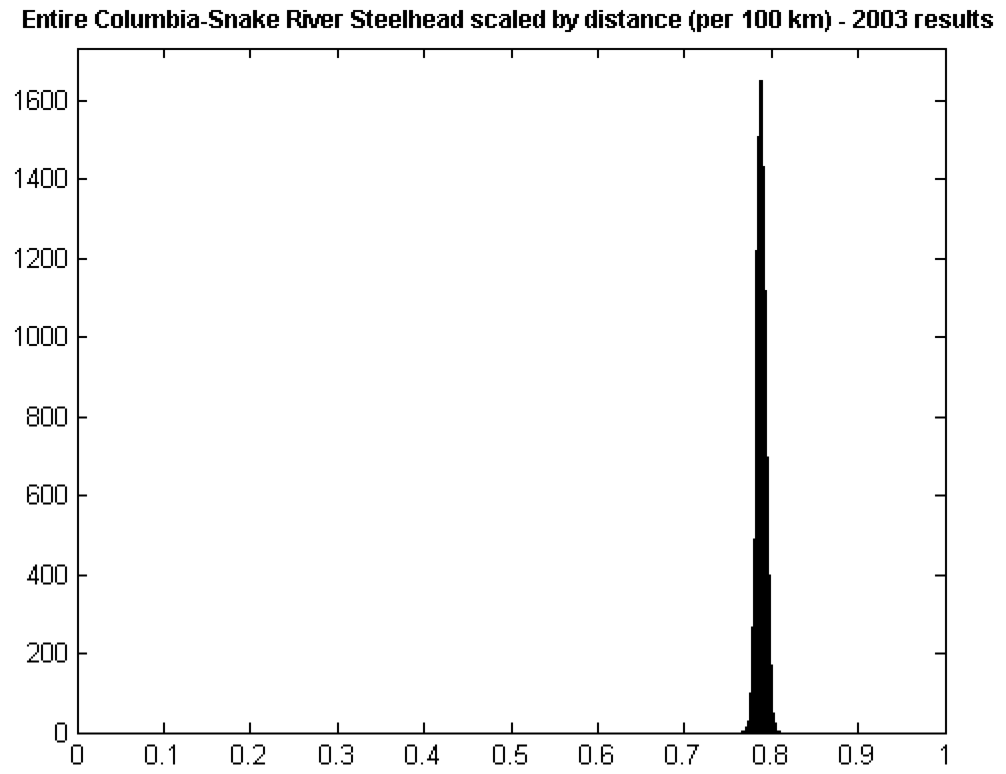

#6COLUMBIA-SNAKE ENTIRE STEELHEAD comparison with FRASER STEELHEAD scaled by distance (per 100 km)

```
%differences between pairs (CR avg. surv - FR avg. surv)
V_CR_St_Entire_dist=zeros(1,10000);
for kk=1:10000

V_CR_St_Entire_dist(kk)=mean([V_CR_St_Entire_dist_2002(kk),V_CR_St_Enti
re_dist_2003(kk)]);
```

```
end
V_diff_6 = V_CR_St_Entire_dist-V_FR_St_dist;
hist(V_diff_6,50);h = findobj(gca,'Type','patch');set(h,'FaceColor',[.8
.8 .8]);
title('\bfDifferences between Entire COlumbia-Snake R and FR scaled by
distance (per 100 km)','fontsize',9);
S=sort(V_diff_6);
disp('Lower 95% CI is ');disp(S(250))
disp('Upper 95% CI is ');disp(S(9750))
axis([-0.1  max(S)+0.1  0  1.05*max(hist(V_diff_6,50))]);
Lower 95% CI is
    0.0482

Upper 95% CI is
    0.2455
```

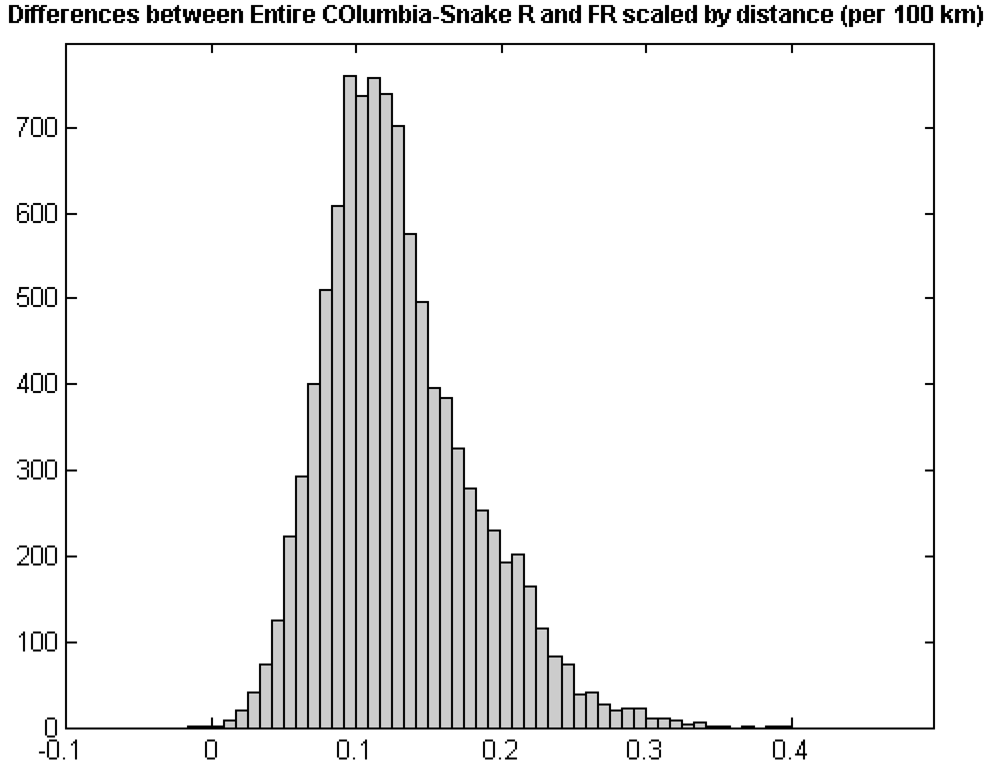

FRASER RIVER STEELHEAD scaled by time (per day)

```
FR_St_time = [5.71,6.17,8.5,10.52,3.89,2.9,10.39];%Kintama results
V_FR_St_time = zeros(1,10000);
y_FR_St_time = zeros(10000,7);
for kk=1:10000
    for ii=1:length(FR_St_surv)
        y_FR_St_time(kk,ii) = (y_FR_St(kk,ii))^(1/FR_St_time(ii));
        V_FR_St_time(kk)=mean(y_FR_St_time(kk,:));
    end
end
hist(V_FR_St_time,20); h =
findobj(gca,'Type','patch');set(h,'FaceColor',[.8 .8 .8]);
title('\bfFraser River Steelhead scaled by time (per
day)','fontsize',9);
disp('The mean value for FRASER RIVER STEELHEAD survival estimates
scaled by time (per day) is ');disp(mean(V_FR_St_time))
S=sort(V_FR_St_time);
disp('Lower 95% CI is ');disp(S(250))
disp('Upper 95% CI is ');disp(S(9750))
axis([0 1 0 1.05*max(hist(V_FR_St_time,20))]);
The mean value for FRASER RIVER STEELHEAD survival estimates scaled by
time (per day) is
    0.7575

Lower 95% CI is
    0.6294

Upper 95% CI is
    0.8251
```

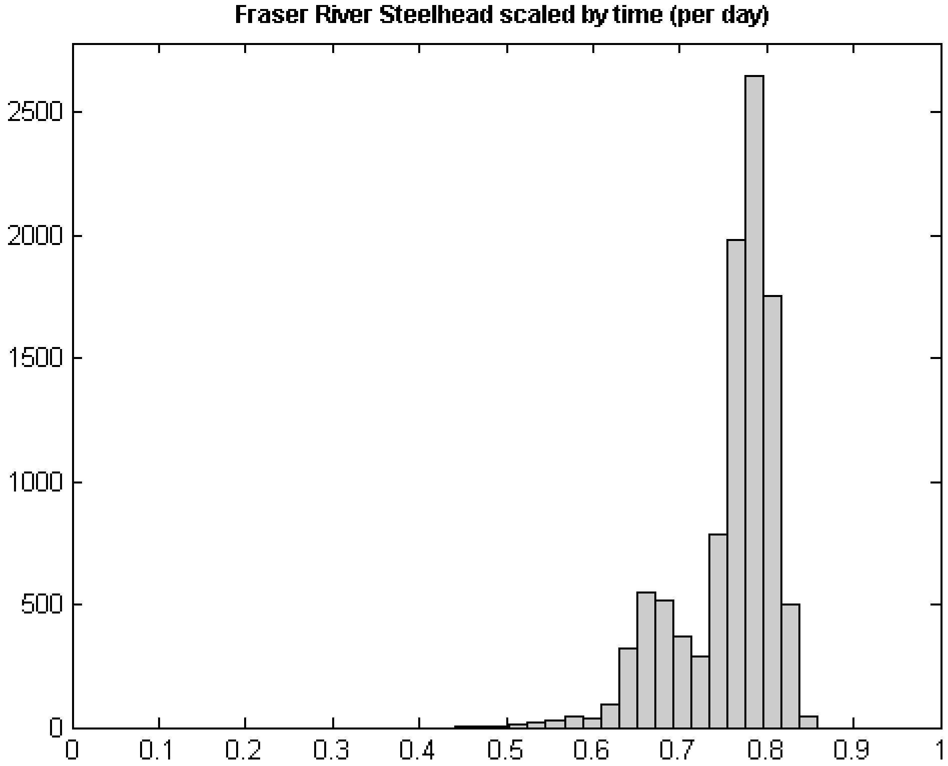

SNAKE RIVER STEELHEAD IMPOUNDED scaled by time (per day)

```
CR_St_Upper_time = [14.38, 18.63, 15.63, 17.05, 32.19, 20.37, 19.91,
12.78];%PIT results
V_CR_St_Upper_time = zeros(1,10000);
y_CR_St_Upper_time = zeros(10000,8);
for kk=1:10000
    for ii=1:length(CR_St_Upper_surv)
        y_CR_St_Upper_time(kk,ii) =
(y_CR_St_Upper(kk,ii))^(1/CR_St_Upper_time(ii));
        V_CR_St_Upper_time(kk)=mean(y_CR_St_Upper_time(kk,:));
    end
end
```

```
hist(V_CR_St_Upper_time,20);h =
findobj(gca,'Type','patch');set(h,'FaceColor',[.8 .8 .8]);
title('\bfSnake River Steelhead Impounded scaled by time (per
day)','fontsize',9);
disp('The mean value for SNAKE RIVER STEELHEAD IMPOUNDED survival
estimates scaled by time (per day) is ');disp(mean(V_CR_St_Upper_time))
S=sort(V_CR_St_Upper_time);
disp('Lower 95% CI is ');disp(S(250))
disp('Upper 95% CI is ');disp(S(9750))
axis([0 1 0 1.05*max(hist(V_CR_St_Upper_time,20))]);
The mean value for SNAKE RIVER STEELHEAD IMPOUNDED survival estimates
scaled by time (per day) is
    0.9374

Lower 95% CI is
    0.9328

Upper 95% CI is
    0.9417
```

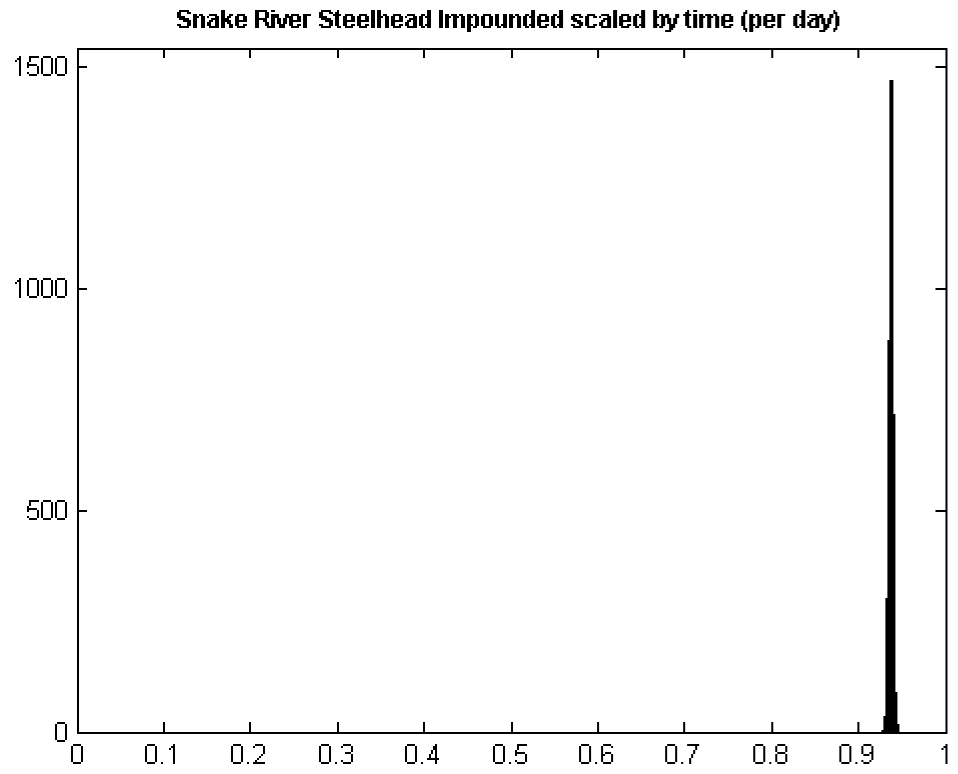

#7SNAKE RIVER STEELHEAD IMPOUNDED comparison with FRASER STEELHEAD scaled by time (per day)

```
%differences between pairs (CR avg. surv - FR avg. surv)
V_diff_7 = V_CR_St_Upper_time-V_FR_St_time;
hist(V_diff_7,50);h = findobj(gca,'Type','patch');set(h,'FaceColor',[.8
.8 .8]);
title('\bfDifferences between Snake R Steelhead Impounded and FR scaled
by time (per day)','fontsize',9);
S=sort(V_diff_7);
disp('Lower 95% CI is ');disp(S(250))
disp('Upper 95% CI is ');disp(S(9750))
axis([-0.1  max(S)+0.1  0  1.05*max(hist(V_diff_7,50))]);
Lower 95% CI is
    0.1121

Upper 95% CI is
    0.3089
```

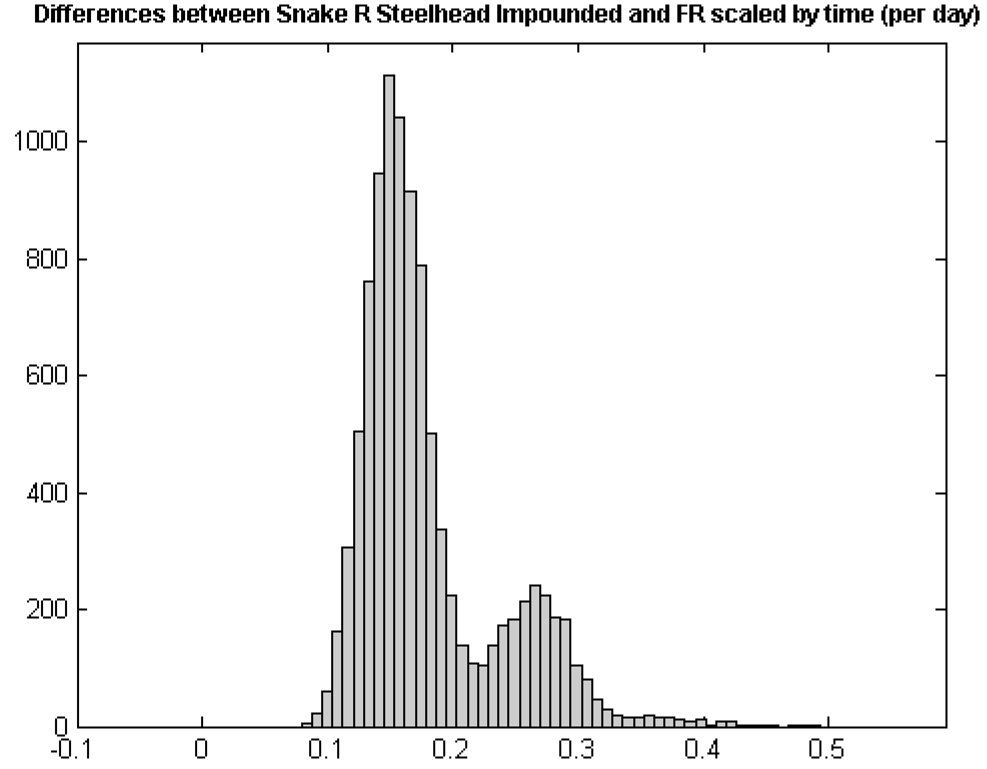

SNAKE RIVER STEELHEAD UNIMPOUNDED scaled by time (per day)

```
CR_St_Lower_time = [2.77, 2.66];
V_CR_St_Lower_time = zeros(1,10000);
y_CR_St_Lower_time = zeros(10000,2);
for kk=1:10000
    for ii=1:length(CR_St_Lower_surv)
        y_CR_St_Lower_time(kk,ii) =
(y_CR_St_Lower(kk,ii))^(1/CR_St_Lower_time(ii));
        V_CR_St_Lower_time(kk)=mean(y_CR_St_Lower_time(kk,:));
    end
end
hist(V_CR_St_Lower_time,20);h =
findobj(gca,'Type','patch');set(h,'FaceColor',[.8 .8 .8]);
title('\bfSnake River Steelhead Unimpounded scaled by time (per
day)','fontsize',9);
disp('The mean value for SNAKE RIVER STEELHEAD UNIMPOUNDED survival
estimates scaled by time (per day) is ');disp(mean(V_CR_St_Lower_time))
S=sort(V_CR_St_Lower_time);
disp('Lower 95% CI is ');disp(S(250))
disp('Upper 95% CI is ');disp(S(9750))
axis([0 1 0 1.05*max(hist(V_CR_St_Lower_time,20))]);
The mean value for SNAKE RIVER STEELHEAD UNIMPOUNDED survival estimates
scaled by time (per day) is
    0.8728

Lower 95% CI is
    0.8501

Upper 95% CI is
    0.8940
```

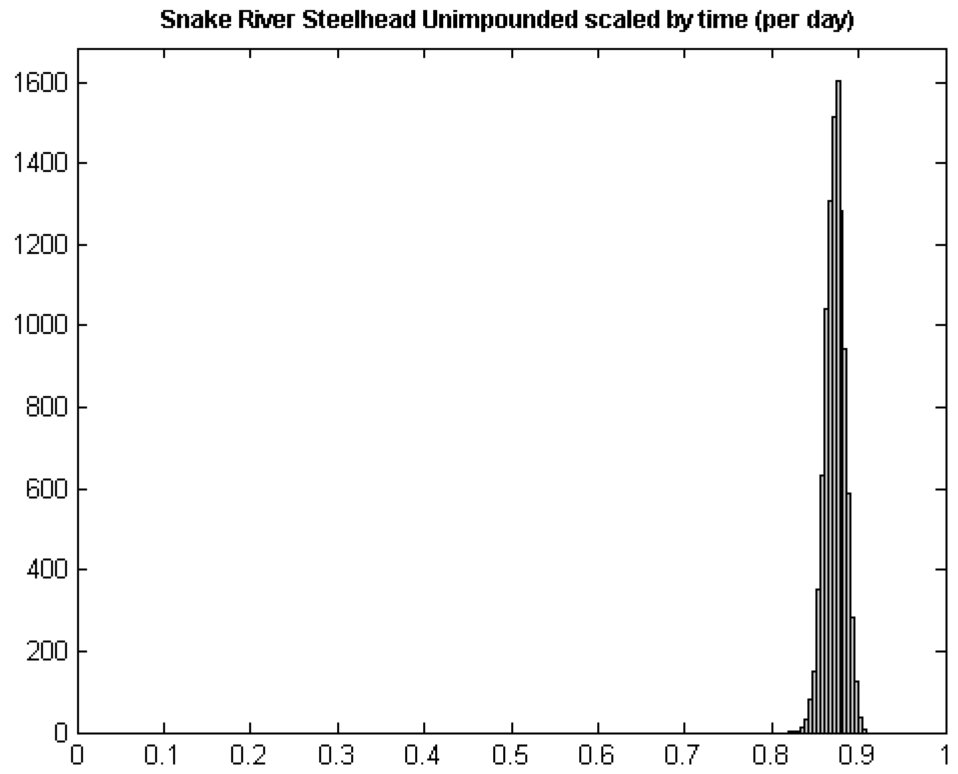

#8SNAKE RIVER STEELHEAD UNIMPOUNDED comparison with FRASER STEELHEAD scaled by time (per day)

```
%differences between pairs (CR avg. surv - FR avg. surv)
V_diff_8 = V_CR_St_Lower_time-V_FR_St_time;
hist(V_diff_8,50);h = findobj(gca,'Type','patch');set(h,'FaceColor',[.8
.8 .8]);
title('\bfDifferences between Columbia-Snake R Unimpounded and FR
scaled by time (per day)','fontsize',9);
S=sort(V_diff_8);
disp('Lower 95% CI is ');disp(S(250))
disp('Upper 95% CI is ');disp(S(9750))
axis([-0.1  max(S)+0.1  0  1.05*max(hist(V_diff_8,50))]);
```

```
Lower 95% CI is
    0.0429

Upper 95% CI is
    0.2485
```

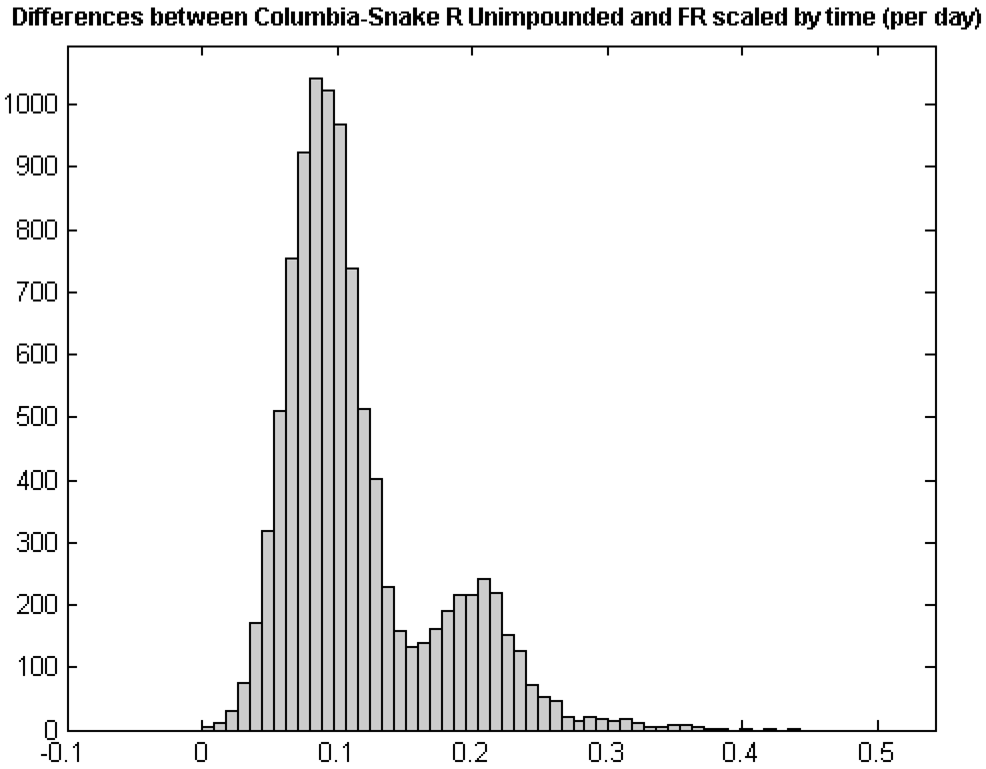

COLUMBIA-SNAKE ENTIRE STEELHEAD scaled by time (per day) 2002

```
%the 2002 synthetic results
V_CR_St_Entire_time_2002=(y_CR_St_Lower(:,1).*y_CR_St_Upper(:,6)).^(1/(
20.37+2.77));
hist(V_CR_St_Entire_time_2002,20);h =
findobj(gca,'Type','patch');set(h,'FaceColor',[.8 .8 .8]);
title('\bfEntire Columbia-Snake River Steelhead scaled by time (per
day) - 2002 results','fontsize',9);
disp('The mean value for COLUMBIA-SNAKE ENTIRE STEELHEAD survival
estimates scaled by time (per day) 2002 is ')
disp(mean(V_CR_St_Entire_time_2002))
S=sort(V_CR_St_Entire_time_2002);
disp('Lower 95% CI is ');disp(S(250))
disp('Upper 95% CI is ');disp(S(9750))
axis([0 1 0 1.05*max(hist(V_CR_St_Entire_time_2002,20))]);
The mean value for COLUMBIA-SNAKE ENTIRE STEELHEAD survival estimates
scaled by time (per day) 2002 is
    0.9273

Lower 95% CI is
    0.9097

Upper 95% CI is
    0.9419
```

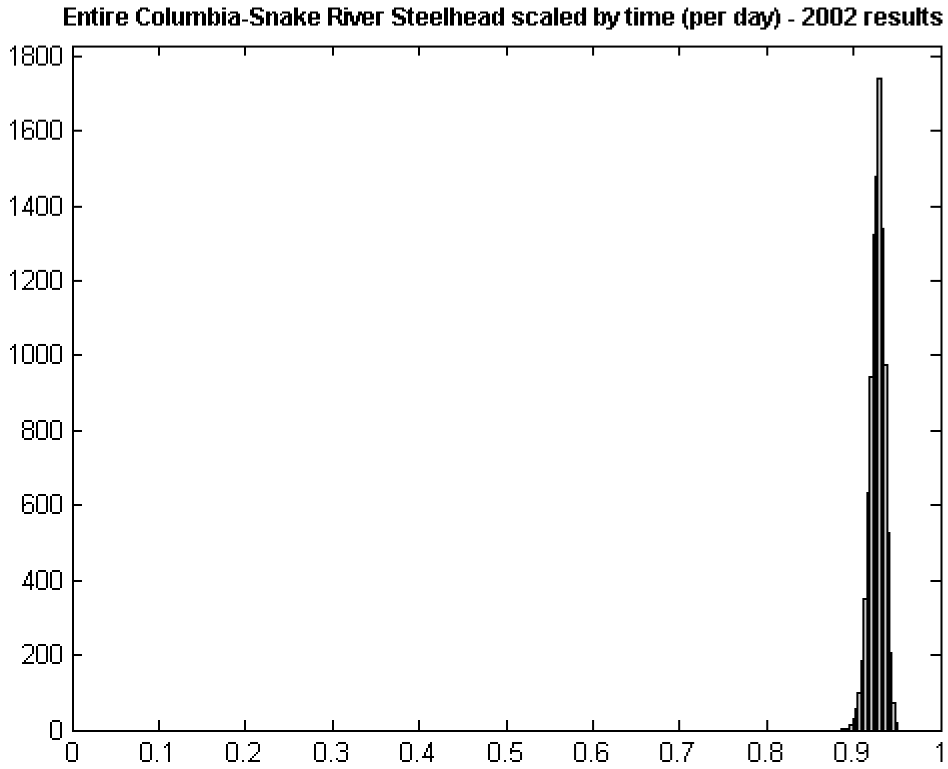

COLUMBIA-SNAKE ENTIRE STEELHEAD scaled by time (per day) 2003

```
%the 2003 synthetic results
V_CR_St_Entire_time_2003=(y_CR_St_Lower(:,2).*y_CR_St_Upper(:,7)).^(1/(
19.91+2.66));
hist(V_CR_St_Entire_time_2003,20);h =
findobj(gca,'Type','patch');set(h,'FaceColor',[.8 .8 .8]);
title('\bfEntire Columbia-Snake River Steelhead scalea by time (per
day) - 2003 results','fontsize',9);
disp('The mean value for COLUMBIA-SNAKE ENTIRE STEELHEAD survival
estimates scaled by time (per day) 2003 is ')
disp(mean(V_CR_St_Entire_time_2003))
S=sort(V_CR_St_Entire_time_2003);
disp('Lower 95% CI is ');disp(S(250))
disp('Upper 95% CI is ');disp(S(9750))
axis([0 1 0 1.05*max(hist(V_CR_St_Entire_time_2003,20))]);
The mean value for COLUMBIA-SNAKE ENTIRE STEELHEAD survival estimates
scaled by time (per day) 2003 is
    0.9271

Lower 95% CI is
    0.9232

Upper 95% CI is
    0.9310
```

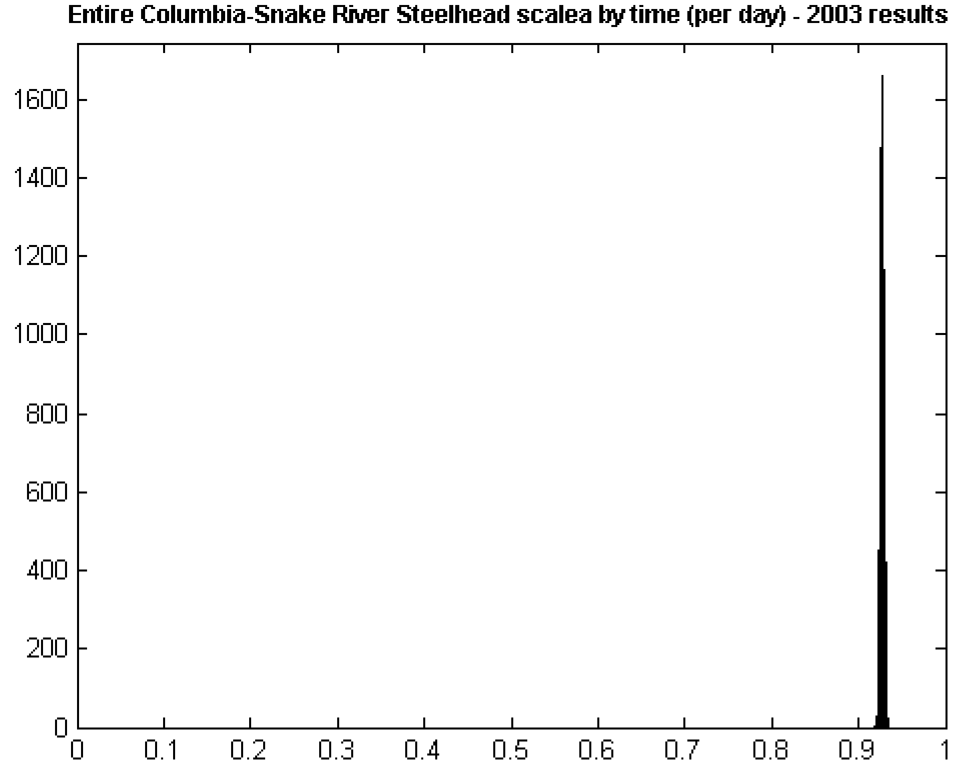

#9COLUMBIA-SNAKE ENTIRE STEELHEAD comparison with FRASER STEELHEAD scaled by time (per day)

```
%differences between pairs (CR avg. surv - FR avg. surv)
V_CR_St_Entire_time=zeros(1,10000);
for kk=1:10000

V_CR_St_Entire_time(kk)=mean([V_CR_St_Entire_time_2002(kk),V_CR_St_Enti
re_time_2003(kk)]);
end
V_diff_9 = V_CR_St_Entire_time-V_FR_St_time;
```

```
hist(V_diff_9,50);h = findobj(gca,'Type','patch');set(h,'FaceColor',[.8
.8 .8]);
t=title('\bfDifferences between Entire Columbia-Snake R and FR scaled
by time (per day)','fontsize',9);
S=sort(V_diff_9);
disp('Lower 95% CI is ');disp(S(250))
disp('Upper 95% CI is ');disp(S(9750))
axis([-0.1  max(S)+0.1  0  1.05*max(hist(V_diff_9,50))]);
Lower 95% CI is
    0.1015

Upper 95% CI is
    0.2993
```

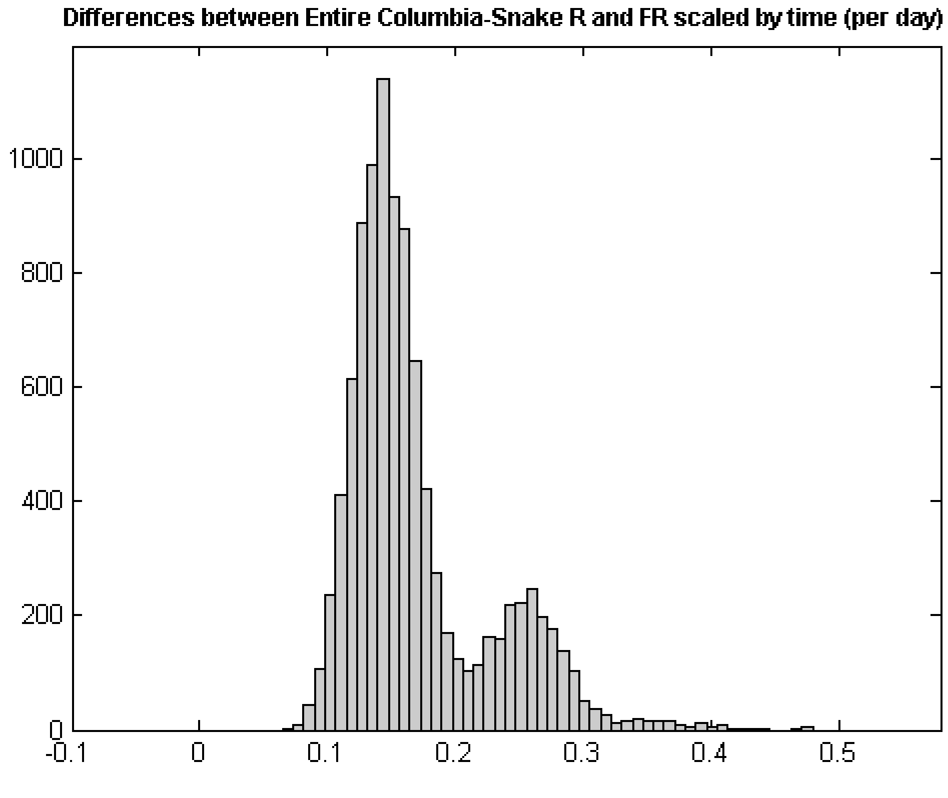

Published with MATLAB® 7.4
